# Peptidomimetic Small-Molecule Inhibitors of 3CLPro Activity and Spike–ACE2 Interaction: Toward Dual-Action Molecules against Coronavirus Infections

**DOI:** 10.1021/acs.joc.2c01047

**Published:** 2022-08-30

**Authors:** Filomena Tedesco, Lorenzo Calugi, Elena Lenci, Andrea Trabocchi

**Affiliations:** Department of Chemistry “Ugo Schiff”, University of Florence, via della Lastruccia 13, Sesto Fiorentino, 50019 Florence, Italy

## Abstract

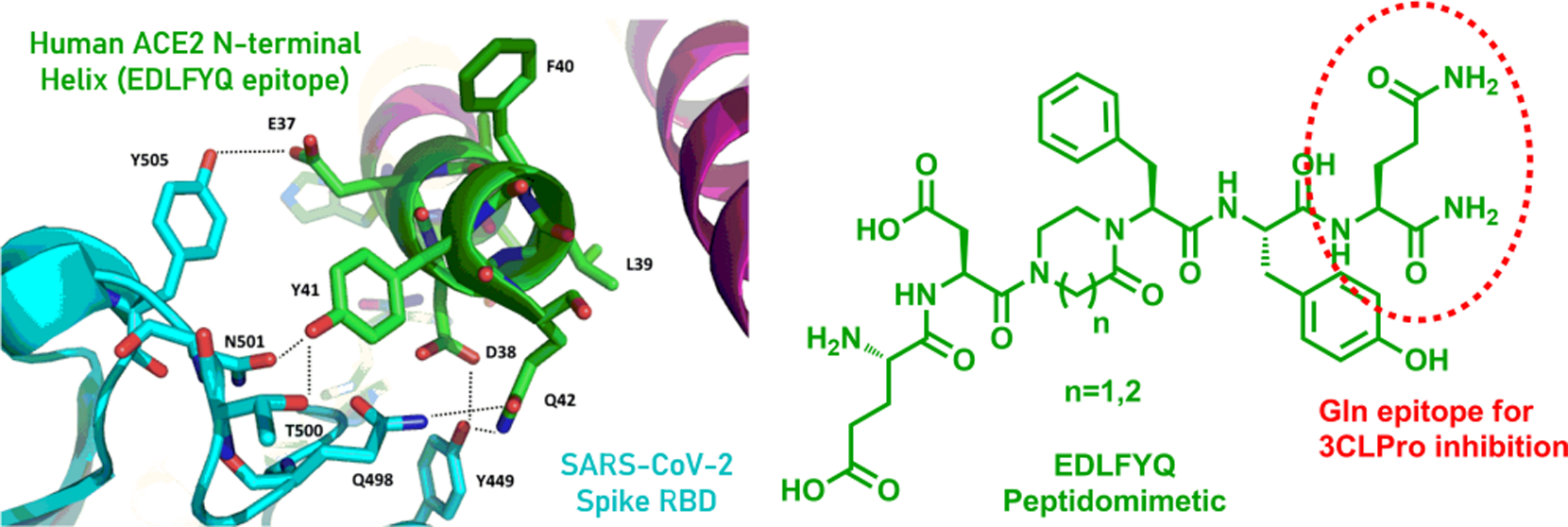

The development of molecules able to target protein–protein interactions (PPIs) is of interest for the development of novel therapeutic agents. Since a high percentage of PPIs are mediated by α-helical structure at the interacting surface, peptidomimetics that reproduce the essential conformational components of helices are useful templates for the development of PPIs inhibitors. In this work, the synthesis of a constrained dipeptide isostere and insertion in the short peptide epitope EDLFYQ of the angiotensin-converting enzyme 2 (ACE2) α1 helix domain resulted in the identification of a molecule capable of inhibiting the SARS-CoV-2 ACE2/spike interaction in the micromolar range. Moreover, inhibition of SARS-CoV-2 3CLPro main protease activity was assessed as an additional inhibitory property of the synthesized peptidomimetics, taking advantage of the C-terminal Q amino acid present in both the ACE2 epitope and the Mpro recognizing motif (APSTVxLQ), thus paving the way to the development of multitarget therapeutics toward coronavirus infections.

## Introduction

**Figure 1:**
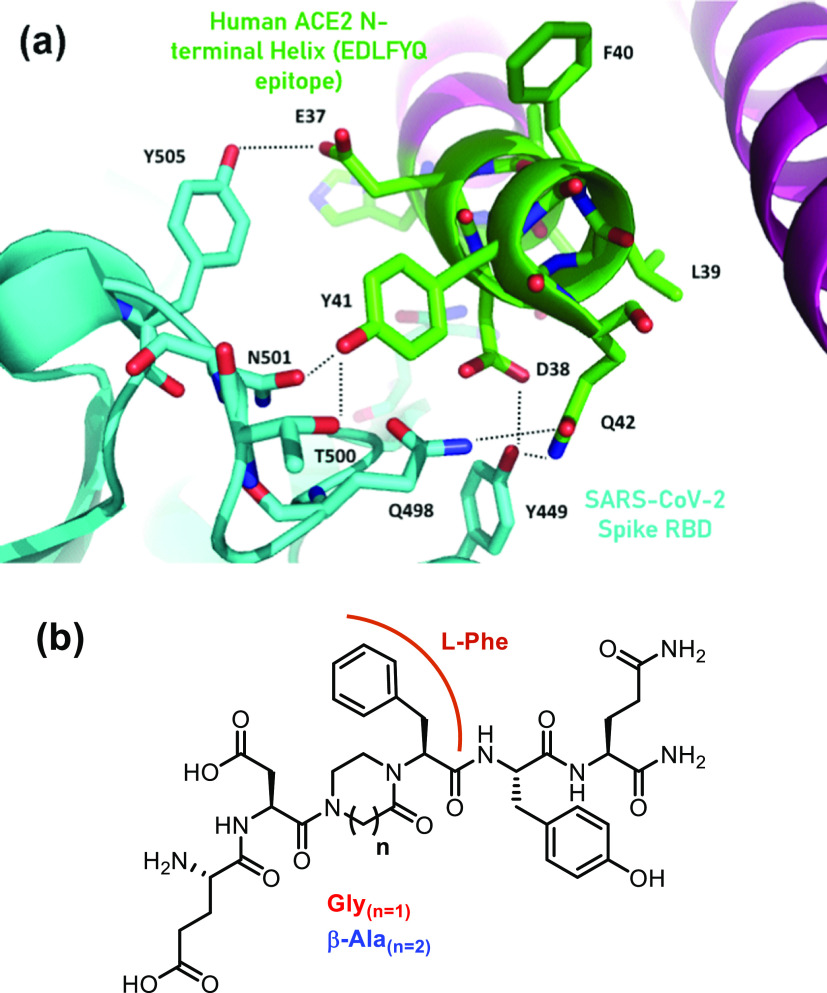
(a) Interaction between host angiotensin-converting enzyme 2 (ACE2) receptor and SARS-CoV-2 spike through its receptor-binding domains (RBDs). (b) Peptidomimetic of the 6-amino-acid ACE2 motif (37-EDLFYQ-42) identified as a key epitope for SARS-CoV-2 inhibition.

The COVID-19^1,2^ pandemic disease caused by severe acute respiratory syndrome coronavirus 2 (SARS-CoV-2) has been responsible for over 500 million infections and 6 million confirmed deaths worldwide, as of April 2022.^3^ Although the introduction of vaccines^3^ has made it possible to reduce serious and critical cases to 0.1% among actually infected people,^4^ immunosuppressed or unvaccinated subjects still represent a vulnerable target, and there is concern for new genetic variants of SARS-CoV-2 that continue to emerge.^5^ Thus, the implementation of therapeutic approaches is needed to complement vaccine development. To date, a series of therapies are being employed for the treatment of COVID-19^6^ that include antivirals, inflammation inhibitors, antirheumatic drugs, plasma, and therapeutic antibodies. The greatest efforts toward blocking viral replication are focused on key events linked to the infection, specifically oriented to the blockade of the spike–angiotensin-converting enzyme 2 (ACE2) interaction^7^ and the inhibition of the viral proteins necessary for replication, such as the 3CLPro protease^8,9^ and the RNA-dependent RNA polymerase (RdRp).^10^ Accordingly, several small-molecule inhibitors of both 3CLPro and RdRP proteins have been identified, and three direct-acting antivirals have been already approved, such as Paxlovid containing nirmatrelvir (PF-07321332) as a 3CLPro inhibitor, and both Molnupiravir and Remdesivir as RdRp inhibitors.^6^ The critical step during the infection of human cells is driven by the binding of the viral spike protein of SARS-CoV-2 to the human cell surface receptor ACE2, making such interaction a valid target to develop cell entry inhibitors of SARS-CoV-2 infection. In the work by Zhou,^11^ the structure and characterization of the complex ACE2-fragment S1 of the SARS-CoV-2 spike protein, obtained with the cryo-EM technique, was reported. This protein–protein interaction (PPI) is characterized by SARS-CoV-2 spike protein region binding domain (RBD) interacting with the exposed domain of ACE2 with the N- and C-terms of the α1 helix. From these data, it was possible to identify an epitope on which to lay the basis for the development of inhibitors of the ACE2-spike protein S interaction of SARS-CoV-2, as similarly seen previously for anti-SARS-n-CoV epitopes.^12^ From the analysis of initial works as reported in the literature identifying short epitopes of the ACE2 α1 helix,^13^ it was decided to design a peptidomimetic of the sequence H-^37^EDLFYQ^42^-NH_2_, as shown in Figure 1a. Specifically, Larue and collaborators^13^ rationally designed and tested a series of small peptide inhibitors of the spike-ACE2 interaction, based on the sequence of ACE2 α1 helix, resulting in the identification of this N-terminal epitope possessing inhibition potency toward SARS-CoV-2 infection in the millimolar range. With the aim to constraining this sequence and improving the druggability of such epitope, we selected the Leu-Phe dipeptide sequence for replacement with dipeptide isosteres, as this sequence was found not directly interacting with the RBD of spike S1 domain (Figure 1).

**Figure 2:**
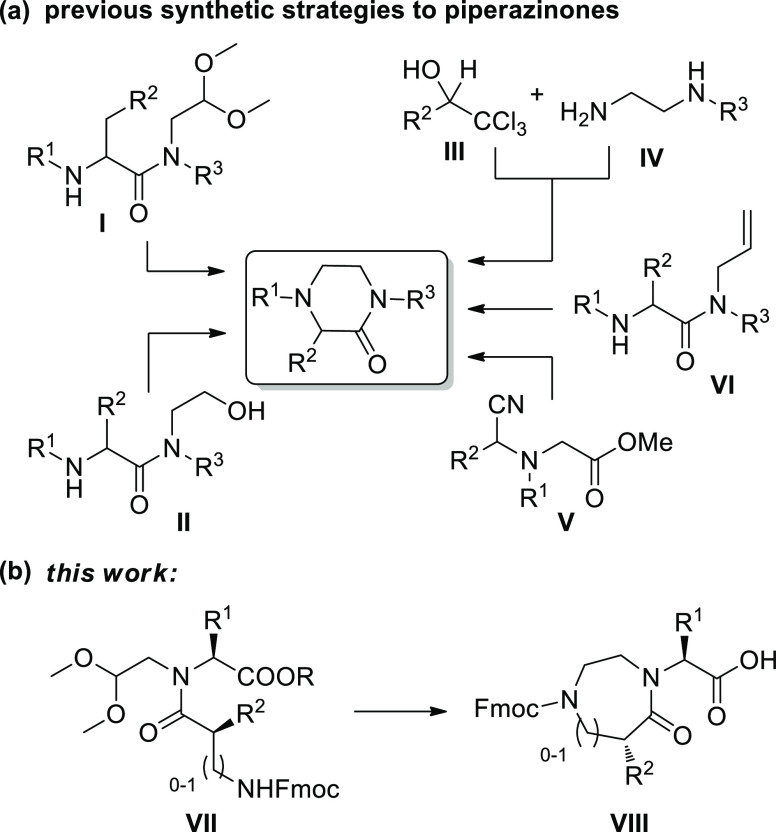
(a) Previously reported synthetic strategies for the achievement of piperazinones. (b) Synthesis of differently substituted piperazinones and diazepanones starting from *N*-(2,2-dimethoxyethyl)acetamides.

Over the last years, several dipeptide isosteres have been proposed as peptidomimetic building blocks, some of them being used also in a repetitive manner to reproduce the conformational components of helices.^14−16^ Starting from the terphenyl scaffold developed by Hamilton,^17^ other scaffolds with rodlike structure have been developed to improve their water solubility and to reduce their synthetic complexity, including pyridazine derivatives,^18^ terpyridyls,^19^ benzoylureas,^20^ and piperazinones.^21^ The latter, first reported by Arora and co-workers,^21^ possess a highly structured rodlike arrangement able to reproduce the spatial orientation of *i*, *i* + 4, and *i* + 7 key side chains on an α-helix. Several synthetic approaches for the achievement of piperazinone scaffolds have been reported in the literature (Figure 2a), such as those involving the 2-amino-*N*-(2,2-dimethoxyethyl)acetamides **I** through the use of cyclic iminium intermediates, followed by hydrogenation,^22^ including in a solid-phase synthetic strategy,^23^ the Mitsunobu alkylation between amide and alcohol functions of derivative **II**,^24,25^ Jocic-type reactions of enantiomerically enriched trichloromethyl-substituted alcohol **III** (95% enantiomeric excess (ee)) with unsymmetrical mixed-primary-secondary 1,2-diamine **IV**,^26^ a reductive cyclization of cyanomethylamino pseudopeptide **V**,^27^ and reductive amination of allyl-containing peptide **VI** with ozone.^21^

Thus, starting from our expertise in the preparation of dipeptide isosteres,^28−30^ we envisioned to develop a short and versatile synthetic strategy for the formation of differently substituted piperazinones and diazepanones **VIII** starting from *N*-(2,2-dimethoxyethyl)acetamides **VII** (Figure 2b). These constrained dipeptide isosteres were then inserted in the selected ACE2 epitope EDLFYQ to evaluate their potential activity as inhibitor of the ACE2–spike S1 interaction and as inhibitor of the activity of 3CLPro viral enzyme, based on a terminal glutamine in the peptide sequence essential for molecular recognition.^a^

## Results and Discussion

**Scheme 1:**
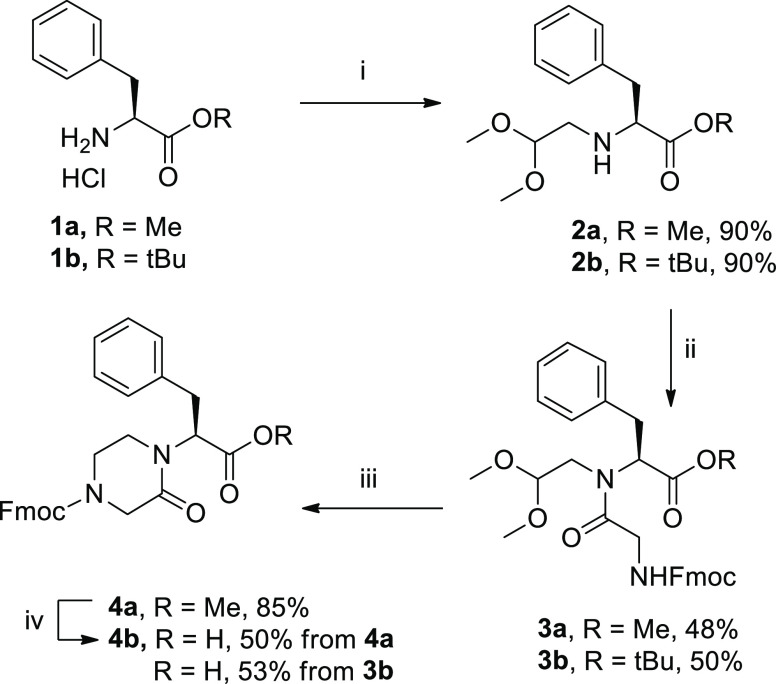
Synthesis of Piperazinone Skeleton 4 Reagents and conditions: (i) dimethoxyacetaldehyde, H_2_, Pd/C 10%, MeOH, r.t., 16 h; (ii) Fmoc-Gly-OH, HATU, DIPEA, dry dimethylformamide (DMF), r.t., 16 h; (iii) TFA, TIPS, 1,2-DCE, r.t., 16 h; (iv) 5 M HCl solution in dioxane, reflux, 16 h.

As a starting point of the synthesis, we reasoned to modulate the reaction conditions to drive the selectivity of the cyclization toward the piperazinone heterocycle. As a case study, we prepared the glycyl-*N*-(2,2-dimethoxyethyl)-l-phenylalaninate **3a**, containing both phenylalanine and glycine residues. The synthesis of compound **3a** was achieved by a reductive amination of phenylalanine methyl ester (**1a**) with dimethoxyacetaldehyde using hydrogen and Pd/C as a catalyst, followed by a coupling reaction of **2a** with Fmoc-Gly-OH, using 1-[bis(dimethylamino)methylene]-1*H*-1,2,3-triazolo[4,5-*b*]pyridinium 3-oxide hexafluorophosphate (HATU) and *N*,*N*-diisopropylethylamine (DIPEA) at room temperature (r.t.) (Scheme 1). The yield of the coupling step (48%) could not be improved, even using high temperature and microwave irradiation, or other coupling agent, such as (1-cyano-2-ethoxy-2-oxoethylidenaminooxy)dimethylamino-morpholino-carbenium hexafluorophosphate (COMU) or diisopropylcarbodiimide (DIC)–ethyl (hydroxyimino)cyanoacetate (Oxyma). Then, different reaction conditions for the cyclization of compound **3a** were studied, to obtain in a single step the condensation of the newly released aldehyde with the *N*-Fmoc amine functionality, the rearrangement of the cyclic iminium intermediate, and the reduction of the internal double bond. Thus, various mixtures of trifluoroacetic acid (TFA)/triethylsilane (TES) or triisopropylsilane (TIPS)/CH_2_Cl_2_ were tested, under different times and temperatures, to maximize the formation of the target piperazinone **4a**, versus the dihydropirazinone or the nonrearranged amino alcohol (Table 1). The application of the same reaction conditions reported by Krchňák and co-workers,^23^ gave compound **4a** in 50% yield, together with 19% of dihydropirazinone **5a** (entry 1), whereas the substitution of TES with TIPS resulted in a slight improvement of the selectivity toward compound **4a** (entry 2). The increase of this reductive agent from 10 to 40% (entry 3) and of the reaction time from 4 to 16 h (entry 4) allowed us to obtain selectively compound **4a** in 70 and 75% yields, respectively. Finally, the use of 1,2-dichloroethane in place of dichloromethane was selected for better solubilizing the intermediate **3a**, increasing the yield from 75 to 85% (entry 5).

**Table 1:**
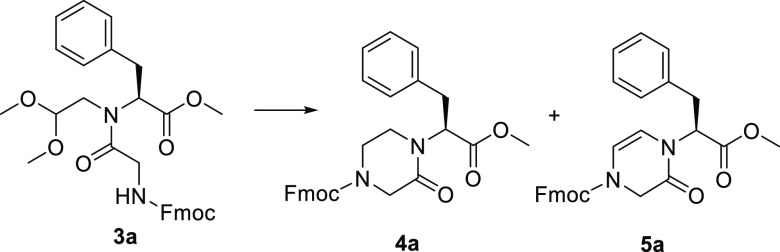
Study of the Cyclization Reaction of **3a** for the Achievement of Piperazinone **4a**

entry	reagents			time (h)	yield (**4a**) (%)	yield (**5a**) (%)
1	TFA 50%	TES 10%	CH_2_Cl_2_ 40%	4	50	19
2	TFA 50%	TIPS 10%	CH_2_Cl_2_ 40%	4	66	17
3	TFA 50%	TIPS 40%	CH_2_Cl_2_ 10%	4	70	0
4	TFA 50%	TIPS 40%	CH_2_Cl_2_ 10%	16	75	0
5	TFA 50%	TIPS 40%	1,2-DCE 10%	16	85	0

Compound **4a** was then transformed into the corresponding acid using 5 M HCl in dioxane (Scheme 1), which was found to be applicable in solid-phase peptide synthesis. The variation of this short synthetic strategy starting from Fmoc-Phe-O*t*Bu **1b** allowed us to obtain directly the oxopiperazine skeleton **4b** in just three synthetic steps (with an overall yield of 24%), even though this compound was found to be difficult to purify from TIPS by-products and not sufficiently pure to be applied in solid-phase peptide synthesis.

**Scheme 2:**
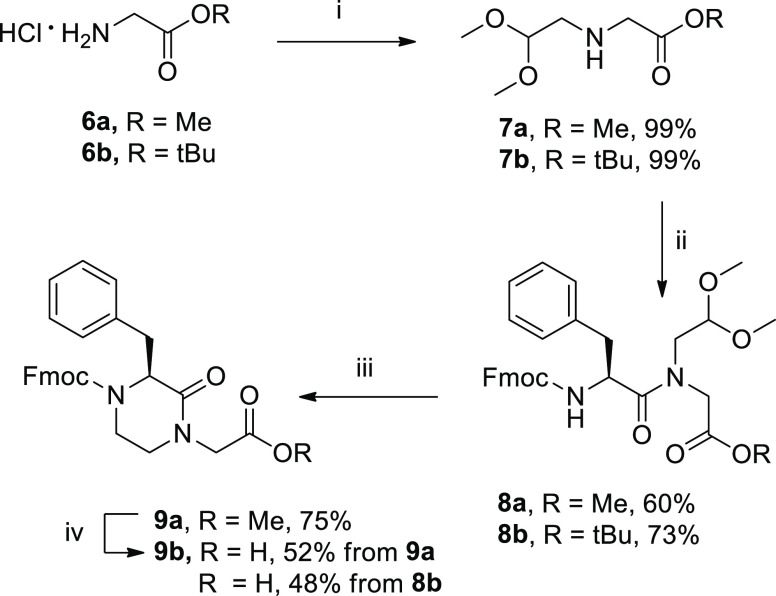
Synthesis of (*S*)-3-Benzylpiperazin-2-one Skeleton **9** Reagents and conditions: (i) dimethoxyacetaldehyde, TEA, H_2_, Pd/C 10%, MeOH, r.t., 16 h; (ii) Fmoc-l-Phe-Cl, DIPEA, dry CH_2_Cl_2_, r.t., 4 h; (iii) TFA, TIPS, 1,2-DCE, r.t., 16 h; (iv) 5 M HCl solution in dioxane, reflux, 16 h.

With this protocol in our hands, we turned the attention on coupling different amino acids in place of glycine to compound **2**, to obtain a piperazinone skeleton with a substituent in position 1. The high steric hindrance between the aromatic moiety of phenylalanine did not allow us to obtain any coupling product between **2** and different amino acids, including those with a short hydrophobic chain, such as Fmoc-Ala-OH, Fmoc-Val-OH, or Fmoc-Leu-OH. However, the reversal of the synthetic approach, such as combining (2,2-dimethoxyethyl)glycinate to phenylalanine, was found to be a successful route for the synthesis of 3-substituted piperazin-2-one skeleton. As shown in Scheme 2, compound **7a** (prepared by reductive amination of glycine methyl ester **6a** with dimethoxyacetaldehyde) was left reacting with Fmoc-Phe-Cl to give the intermediate **8a** in 70% yield. Amide bond formation via acyl chloride proved to work better than the coupling reaction with Fmoc-Phe-OH using HATU as described above, which resulted in the corresponding product in only 16% yield. The application of the cyclization protocol and of the acid-mediated methyl ester deprotection as described above gave the final (*S*)-3-benzylpiperazin-2-one skeleton **9b** in 39% yield over two steps. The synthetic pathway for achieving (*S*)-3-benzylpiperazin-2-one **9** was also repeated starting from glycine *tert*-butyl ester **6b**. In this way, intermediate **8b**, obtained by acylation of intermediate **7b** with Fmoc-Phe-Cl in 73% yield, was treated with TFA/TIPS mixture to give compound **9b** in a single step in 48% yield (Scheme 2).

**Scheme 3:**
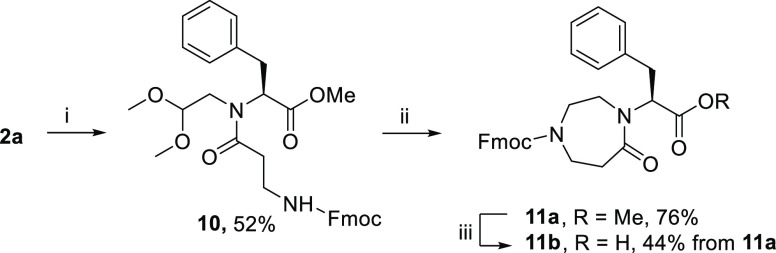
Synthesis of Diazepan-5-one Skeleton **11** Reagents and conditions: (i) Fmoc-β-Ala-OH, HATU, DIPEA, dry DMF, r.t., 16 h; (ii) TFA, TIPS, 1,2-DCE, r.t.; 16 h; (iii) 5 M HCl solution in dioxane, reflux, 16 h, 28 h.

Then, with the aim of obtaining larger heterocycles as dipeptide isosteres, we combined compound **2a** with Fmoc-β-alanine using HATU and DIPEA at room temperature (Scheme 3). Intermediate **10**, obtained in 52% yield, was then cyclized using the same optimized reaction conditions to give the diazepan-5-one **11a** in 76% yield. This scaffold was then transformed into the corresponding carboxylic acid **11b** using a 5 M HCl solution in dioxane in 44% yield. The synthetic strategy previously shown starting from *tert*-butyl ester **2b** was found not to be applicable for the synthesis of this scaffold, as treatment with TFA/TIPS mixture gave **11b** and 1,4,6,7-tetrahydro-5*H*-1,4-diazepin-5-one in an inseparable mixture.

**Figure 3:**
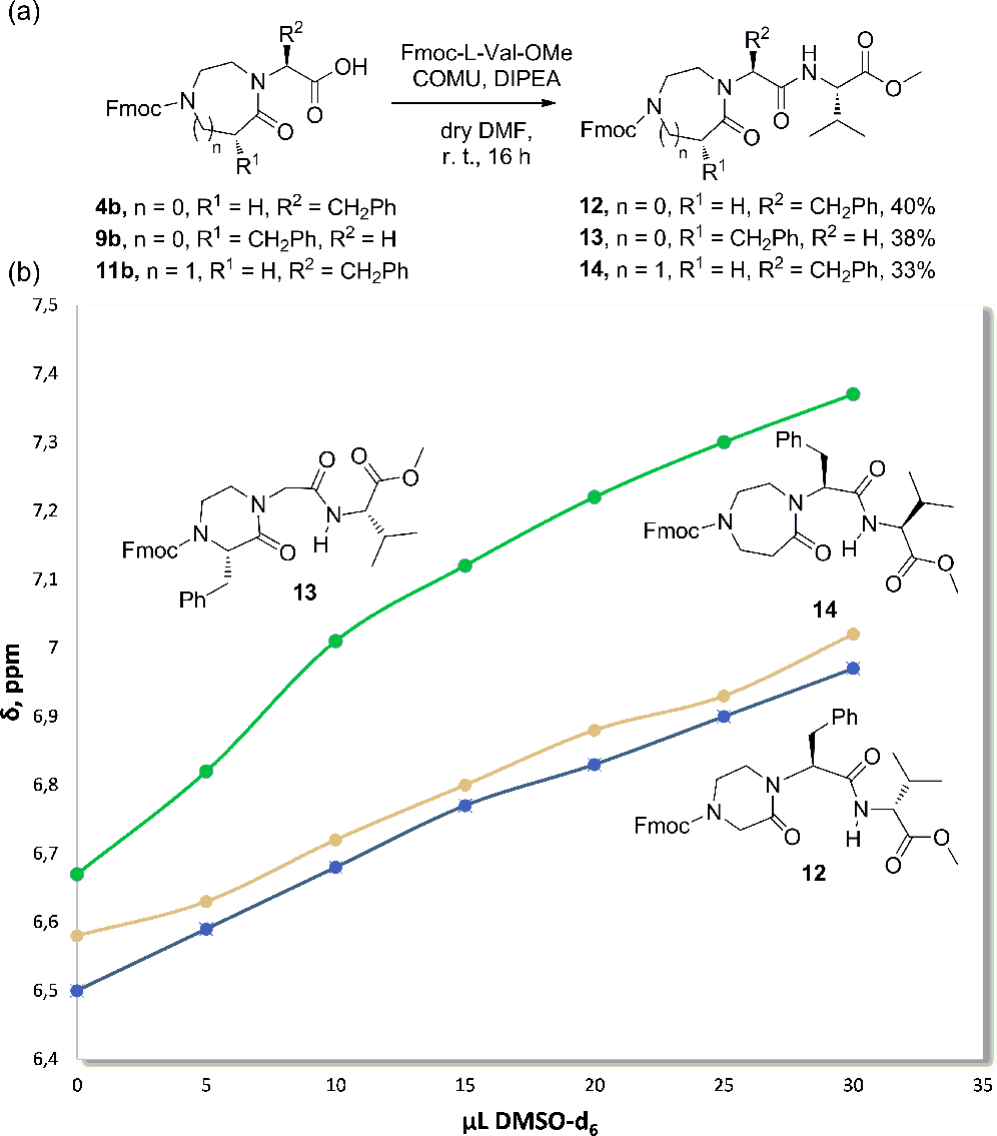
(a) Synthesis of compounds **12**–**14** containing, respectively, the piperazin-2-one, the (*S*)-3-benzylpiperazin-2-one, and the diazepan-5-one skeleton coupled to l-valine. (b) Diagram of ^1^H NMR chemical shifts upon DMSO-*d*_6_ addition to a CDCl_3_ solution (4 mM) of **12** (blue line), **13** (green line), and **14** (orange line).

With the aim of studying the existence of a preferred conformation adopted by the newly synthesized dipeptide isosteres when coupled to another amino acid at the C-terminus side, specifically investigating the possibility to form an internal hydrogen-bonding interaction between the carbonyl moiety of the scaffold and the NH group of the adjacent amino acid, we prepared three novel compounds as model tripeptide mimetics (Figure 3a). The carboxylic moieties of **4b**, **9b**, and **11b** were coupled to Fmoc-Val-OH to obtain compounds **12**–**14** in 33–40% yields. An aliquot of 1 mg each of these compounds was dissolved in 500 μL of deuterated chloroform to give diluted solutions, and ^1^H NMR spectra were recorded after sequential addition of 5 μL of dimethyl sulfoxide (DMSO)-*d*_6_, as a hydrogen-bond competitor. The ^1^H NMR signals attributable to the NH, appearing in the region between 6.50 and 6.80 ppm, were found to be downfield shifted with every DMSO-*d*_6_ addition (Figure 3b). These data suggested that amide protons of compounds **12**–**14** were solvent-exposed and did not show any tendency to form an internal hydrogen-bonding interaction with the carbonyl moiety. In particular, the amide proton of (*S*)-3-benzylpiperazin-2-one in **13** interacted more strongly with the competing solvent in the generation of intermolecular hydrogen bonds, thus resulting in a higher deshielding of their chemical shifts.

**Figure 4:**
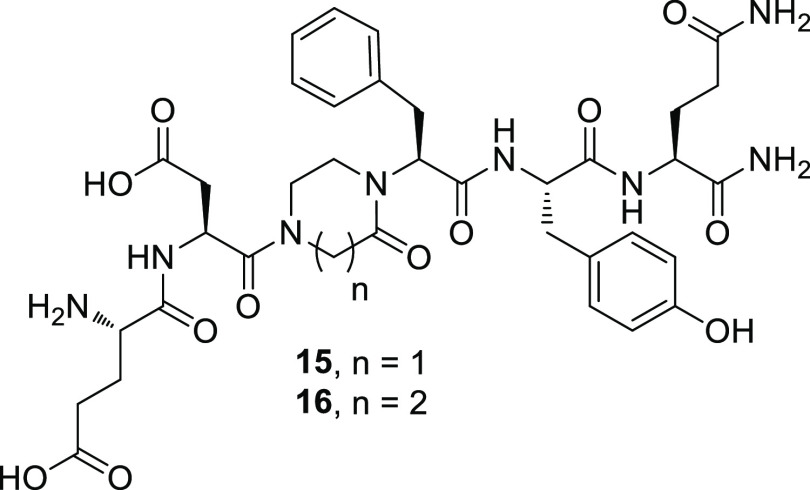
Structure of peptidomimetics **15** and **16**.

With these compounds in our hands, we reasoned to insert the two dipeptide isosteres mimicking the Gly–Phe sequence (**4b** and **11b**) in the sequence H-ED**LF**YQ-NH_2_, a key peptide epitope that was found capable of inhibiting the interaction between the binding region domain (RBD) of SARS-CoV-2 spike protein S1 and ACE2 receptor.^13^ The absence of leucine side chain in the peptidomimetics was not considered an issue, as in the ACE2 α1 helix, it was found pointing outward with respect to the protein interacting surface. Peptidomimetics **15** and **16**, containing **4b** and **11b**, respectively, were obtained by solid-phase peptide synthesis in 24–30% yields (Figure 4).

Finally, peptidomimetics **15** and **16**, together with parent peptide epitope EDLFYQ, were assayed in an enzyme-linked immunosorbent (ELISA)-like solid-phase assay for evaluating the ability of these compounds to interfere with the ACE2–spike S1 RBD protein–protein interaction. The parent epitope and compound **15** showed limited activity in blocking with such interaction at 100 μM concentration, whereas for compound **16**, a significant effect was observed even at a lower concentration, possibly due to increased rigidity of the epitope induced by the constrained dipeptide isostere (Table 2). For this compound, a dose–response measurement using an inhibitor range of concentrations (1 μM to 1 mM) was obtained, with a resulting IC_50_ value of 20 ± 5 μM.

**Table 2:** Inhibitory Activity Data of Peptidomimetics **15** and **16** in Comparison of SAP-6 on the Interaction spike-ACE2 (Determined Using a Solid-Phase ELISA-like Assay) and on 3CLpro (Determined Using a Fluorogenic Peptide Substrate)^a^

compound	% In(spike–ACE2) at 100 μM	% In(3CLPro) at 100 μM
ED**LF**YQ (SAP-6)	25%	0%
ED(**4b)**YQ **15**	0%	90–91%
		IC_50_ = 15 ± 6 μM
ED(**11b)**YQ **16**	66–76%	45–57%
	IC_50_ = 20 ± 5 μM	IC_50_ = 127 ± 76 μM

a Mean from three different assays, errors were in the range of 5–10% of the reported values; IC_50_ values were retrieved from dose–response assays as the concentration of compound required for 50% inhibition, as estimated by nonlinear correlation using GraphPad Prism software.

In view of evaluating a potential dual effect of the peptidomimetics toward ACE2–spike s1 RBD interaction and the viral 3CLPro protease, we also evaluated these compounds for their ability to inhibit 3CLPro enzyme, the main protease of SARS-CoV-2, through a fluorimetric assay using Hilyte Fluor-488-ESATLQSGLRKAK-(QXL-520)-NH_2_ as substrate. The rationale behind such a hypothesis was driven by the existence of structural analogies between the N-terminal α1 helix of ACE2 and protease substrate epitopes, and in particular the concomitant presence of a C-terminal Q amino acid in both the key ACE2 epitope EDLFYQ and the Mpro recognizing motif APSTVxLQ.^31^ Accordingly, a significant inhibition activity was observed for the two peptidomimetics **15** and **16**, with IC_50_ values of 15 ± 6 and 127 ± 76 μM, respectively, whereas no inhibition was observed for the parent peptide epitope, suggesting a key role of the constrained dipeptide isostere in promoting the inhibition activity.

## Conclusions

Following the occurrence of COVID-19 pandemic, the introduction of efficient vaccine strategies resulted in significant reduction in mortality and hospitalization. Nevertheless, implementing therapeutic approaches with direct-acting antivirals to complement vaccine development has become a medical need. In this view, the identification of small molecules able to target protein–protein interactions (PPIs) is of interest for the development of novel therapeutic agents, including the blockade of SARS-CoV-2 viral entry through ACE2–spike RBD interaction. Herein, the synthesis of a pool of Fmoc-protected constrained dipeptide isosteres was achieved in a few steps using iminium chemistry, and the successful insertion in the short peptide epitope EDLFYQ of the ACE2 α-helix 1 domain proved to inhibit the SARS-CoV-2 ACE2/spike interaction in the micromolar range. Moreover, taking advantage of the C-terminal Q amino acid present in both the ACE2 epitope and the Mpro recognizing motif, the inhibition of SARS-CoV-2 3CLPro main protease activity was assessed as an additional inhibitory property of the synthesized peptidomimetics, thus paving the way to the development of multitarget therapeutics towards coronavirus infections.

## Experimental Section

### Synthesis

#### General

Analytical-grade solvents and commercially available reagents were used without further purification. Reactions requiring an inert atmosphere were carried out under a nitrogen atmosphere. ^1^H NMR and ^13^C NMR spectra were recorded on a Varian Mercury 400 (^1^H: 400 MHz, ^13^C: 100 MHz) or a Varian Mercury 200 (^1^H: 200 MHz, ^13^C: 50 MHz). The chemical shifts (δ) and coupling constants (*J*) are expressed in parts per million (ppm) and hertz (Hz), respectively. NMR spectra were collected on a Varian Inova 400 spectrometer operating at 400 MHz for 1H. The spectra of 15 were obtained in 4 mM 90% HDO where aggregation was not significant. TOCSY-ES spectra were recorded with 80 ms mixing time, 2048 points in t1, 256 points in t2, and 32 scans per t2 increment. Two-dimensional (1D) ^1^H NMR spectra of compounds **12**–**14** for determining intramolecular hydrogen bond were obtained at 25 °C as around 3–4 mM CDCl_3_ solution and adding increasing quantities of DMSO-*d*_6_ until 10%. Flash column chromatography (FCC) purifications were performed manually using glass columns with Merck silica gel (40–63 μm) or using the Biotage Isolera automatic system and SNAP silica cartridges. Thin-layer chromatography (TLC) analyses were performed on Merck silica gel 60 F_254_ plates. Optical rotations were measured with JASCO DIP-360 digital polarimeter. Electrospray ionization mass spectrometry (ESI-MS) spectra were recorded on a Thermo Scientific LCQ fleet ion-trap double-quadrupole mass spectrometer using electrospray (ES+) ionization techniques. Dry dichloromethane was obtained through distillation over CaH_2_. Dry tetrahydrofuran (THF) was obtained through distillation over Na/benzophenone. Reactions conducted under microwave irradiation, including solid-phase peptide synthesis, were performed with an automatic single-mode Biotage Initiator Sixty microwave synthesizer equipped with temperature- and pressure-monitoring sensors, using sealed reaction tubes. High-performance liquid chromatography (HPLC) analyses and purifications were performed on synthesized peptides using Dionex Ultimate 3000 system.

#### General Procedure (A) for the Synthesis of Compounds **2a**, **2b** and **7a**, **7b**

A methanolic solution of commercially available amino acid hydrochloride **1a**, **1b**, **6a**, or **6b** (1 equiv) for **2a**, **2b**, **7a**, or **7b**, respectively, was filtered on Amberlist A21 dry ion-exchange resin, and the solvent was removed under reduced pressure. Then, in a double-neck flask, the amino acid was dissolved in MeOH (3 mL/mmol), and a 60% aqueous solution of dimethoxyacetaldehyde (1 equiv) and 10% Pd/C were successively added. The resulting mixture was stirred for 16 h at room temperature under a hydrogen atmosphere. Next, the suspension was filtered over Celite, rinsed with MeOH, and the organic solvent was removed under reduced pressure. The product was used for the following step without further purification.

#### General Procedure (B) for the Synthesis of Compounds **3a**, **3b** and **10a**

To a solution of Fmoc-Gly-OH or Fmoc-β-Ala-OH (1.1 equiv) in dry DMF (2.67 mL/mmol), HATU (1.5 equiv), and DIPEA dry (2 equiv), a solution of compound **2a** or **2b** in dry DMF (2.67 mL/mmol) was added. The resulting mixture was stirred for 16 h at room temperature under a nitrogen atmosphere. Then, the crude was dissolved in Et_2_O and washed with a saturated solution of NaHCO_3_ (3 × 50 mL), 1 M HCl (3 × 50 mL), and brine (1 × 25 mL). The organic phase was dried over Na_2_SO_4_ and concentrated under reduced pressure, then the crude product was purified by flash chromatography.

#### General Procedure (C) for the Synthesis of Compounds **8a** and **8b**

To a solution of compound **7a** or **7b** (1 equiv) in CH_2_Cl_2_ (5 mL/mmol), DIPEA (2 equiv) was added, and the mixture was cooled to 0 °C with an ice bath. Then, a solution of Fmoc-l-phenylalanine hydrochloride (1 equiv) in CH_2_Cl_2_ (5 mL/mmol) was added dropwise over 30 min. The ice bath was removed, and the reaction mixture was left under stirring for 4 h at room temperature. Then, the crude was washed with a saturated solution of NaHCO_3_ (3 × 50 mL), 1 M HCl (3 × 50 mL), and brine (1 × 25). The organic phase was dried over Na_2_SO_4_, concentrated under reduced pressure, and the crude product was purified by flash chromatography.

#### General Procedure (D) for the Synthesis of Compounds **4a**, **9a**, and **11a**

The mixture of TFA, TIPS, and 1,2-DCE (50:40:10, 5.5 mL/mmol) was added to compounds **3a**, **8a**, and **10**, and the resulting mixture was stirred for 16 h at room temperature. The mixture was concentrated under a nitrogen atmosphere, and the crude product was purified by flash chromatography to obtain **4a**, **9a**, and **11a**.

#### General Procedure (E) for the Synthesis of Compounds **4b**, **9b**, and **11b**

Compound **4a**, **9a**, or **11a** (1 equiv) was dissolved in dioxane (2.5 mL/mmol), and 5 M HCl (2.5 mL/mmol) was added. The reaction was refluxed for 16 h using an oil bath and then diluted with 5% Na_2_CO_3_ (50 mL).^32^ The resulting solution was washed with diethyl ether, then the aqueous layer was acidified to pH 1 with 3 M HCl, and the organic phase was extracted with EtOAc. The organic extracts were combined, dried over Na_*2*_SO_4_, and concentrated under reduced pressure. The product was used in the following step without further purification.

#### General Procedure (F) for the Synthesis of Compounds **12**–**14**

To a solution of **4b, 9b**, or **11b** (1 equiv) in dry DMF (20 mL/mmol), COMU (1.5 equiv) and a solution of l-valine methyl ester hydrochloride (1 equiv) and dry DIPEA (6 equiv) in dry DMF (20 mL/mmol) were added. The resulting mixture was stirred for 16 h at room temperature under a nitrogen atmosphere. Successively, the mixture was diluted with EtOAc and water, and the aqueous phase was extracted with EtOAc (3 × 50 mL). Then, the organic phase was washed with NaHCO_3_ (2 × 50 mL), dried over Na_2_SO_4_, concentrated under reduced pressure, and the crude product was purified by flash chromatography.

#### Methyl (2,2-Dimethoxyethyl)-l-phenylalaninate (**2a**)

Compound **2a** (2.69 g) was obtained following the general procedure A as a yellow oil in 90% yield, using **1a** (2.00 g, 11.2 mmol), MeOH (28.9 mL), a 60% aqueous solution of dimethoxyacetaldehyde (1.69 mL, 11.2 mmol) and 10% Pd/C (0.139 g). [α]_D_^24^ = −25.9 (*c* 1.0, CHCl_3_). ^1^H NMR (400 MHz, CDCl_3_) δ 7.27 (d, *J* = 7.2 Hz, 2H), 7.22 (d, *J* = 6.8 Hz, 1H), 7.17 (d, *J* = 7.4 Hz, 2H), 4.40 (t, *J* = 5.5 Hz, 1H), 3.63 (s, 3H), 3.53 (t, *J* = 7.0 Hz, 1H), 3.31 (s, 3H), 3.30 (s, 3H), 2.94 (d, *J* = 7.0 Hz, 2H), 2.74 (dd, *J* = 12.0, 6.0 Hz, 1H), 2.59 (dd, *J* = 12.0, 4.9 Hz, 1H), 1.85 (s, 1H). ^13^C{^1^H} NMR (100 MHz, CDCl_3_) δ 174.7, 137.2, 129.1, 128.4, 126.7, 103.7, 63.0, 53.9, 53.3, 51.6, 49.2, 39.6. ESI-MS (*m*/*z*): 268.15 (100, [M + H]^+^). Anal. Calcd for C_14_H_21_NO_4_: C, 62.90; H, 7.92; N, 5.24. Found: C,63.04; H,8,00; N, 5.19.

#### *tert*-Butyl (2,2-Dimethoxyethyl)-l-phenylalaninate (**2b**)

Compound **2b** (1.26 g) was obtained following the general procedure A as a yellow oil in 90% yield, using **1b** (1.00 g, 4.51 mmol), MeOH (11.6 mL), and a 60% aqueous solution of dimethoxyacetaldehyde (0.68 mL, 4.51 mmol), 10% Pd/C (0.112 g). [α]_D_^24^ = −25.1 (*c* 1.0, CHCl_3_). ^1^H NMR (400 MHz, CDCl_3_) δ 7.21 (dd, *J* = 20.0, 6.6 Hz, 5H), 4.41 (s, 1H), 3.38 (m, 1H), 3.31 (s, 6H), 2.92 (d, *J* = 6.5 Hz, 1H), 2.86 (d, *J* = 7.7 Hz, 1H), 2.73 (d, *J* = 6.2 Hz, 1H), 2.60 (s, 1H), 1.98 (bs, 1H), 1.32 (s, 9H). ^13^C{^1^H} NMR (100 MHz, CDCl_3_) δ 173.5, 137.4, 129.3, 128.2, 126.5, 103.6, 81.1, 63.4, 54.0, 53.1, 49.0, 39.7, 27.9. ESI-MS (*m*/*z*): 310.02 (100, [M + H]^+^). Anal. Calcd for C_17_H_27_NO_4_: C, 65.99; H, 8.80; N, 4.53. Found: C, 66.10; H, 8.93; N, 4.48.

#### Methyl *N*-((((9*H*-Fluoren-9-yl)methoxy)carbonyl)glycyl)-*N*-(2,2-dimethoxyethyl)-l-phenylalaninate (**3a**)

Compound **3a** was obtained following the general procedure B, using **2a** (1.0 g, 3.74 mmol), Fmoc-Gly-OH (1.22 g, 4.11 mmol), HATU (2.13 g, 5.61 mmol), dry DIPEA (1.30 mL, 7.48 mmol), and dry DMF (20 mL). The crude product was purified by flash chromatography (hexane–EtOAc 1:1), to give **3a** (0.98 g) as a white spongy solid in 48% yield. [α]_D_^24^ = −67.9 (*c* 1.0, CHCl_3_). ^1^H NMR (200 MHz, CDCl_3_) mixture of rotamers δ 7.77 (d, *J* = 7.2 Hz, 2H), 7.63 (d, *J* = 7.1 Hz, 2H), 7.48–7.27 (m, 7H), 7.15 (d, *J* = 7.7 Hz, 2H), 5.78 (bs, 1H), 4.39 (d, *J* = 7.0 Hz, 2H), 4.25 (m, 2H), 4.19–4.13 (m, 1H), 4.12–4.04 (m, 1.8H) and 3.97 (s, 0.20H), 3.77 (s, 3H), 3.36 (d, *J* = 3.2 Hz, 1.5H) and 3.42 (s, 0.5H), 3.30 (d, *J* = 12.2 Hz, 5.80H) and 3.48 (s. 0.20), 3.19 (d, *J* = 5.5 Hz, 0.40H) and 3.11 (d, *J* = 5.3 Hz, 0.60H), 2.55 (d, *J* = 4.9 Hz, 0.60H) and 2.47 (d, *J* = 4.7 Hz, 0.40H). ^13^C{^1^H} NMR (50 MHz, CDCl_3_) mixture of rotamers δ 170.4, 169.0, 156.1, 143.9, 141.3, 137.7, 129.0, 128.7, 127.7, 127.0, 126.8, 125.1, 119.9, 103.2, 67.1, 63.8, 55.3, 54.5, 53.6, 51.3, 47.2, 43.0, 34.6. ESI-MS (*m*/*z*): 569.16 (100, [M + Na]^+^). Anal. Calcd for C_31_H_34_N_2_O_7_: C, 68.12; H, 6.27; N, 5.12. Found: C, 68.28; H, 6.30; N, 5.08.

#### *tert*-Butyl *N*-((((9*H*-Fluoren-9-yl)methoxy)carbonyl)glycyl)-*N*-(2,2-dimethoxyethyl)-l-phenylalaninate (**3b**)

Compound **3b** was obtained following the general procedure B, using **2b** (1.0 g, 3.23 mmol), Fmoc-Gly-OH (1.06 g, 3.55 mmol), HATU (1.84 g, 4.85 mmol), dry DIPEA (1.13 mL, 6.46 mmol), and dry DMF (17.3 mL). The crude product was purified by flash chromatography (hexane–EtOAc 2:1), to give **3b** (0.95 g) as a white oil in 50% yield. [α]_D_^24^ = −68.4 (*c* 1.0, CHCl_3_). ^1^H NMR (400 MHz, CDCl_3_) mixture of rotamers δ 7.77 (d, *J* = 7.2 Hz, 2H), 7.62 (t, *J* = 9.6 Hz, 2H), 7.41 (t, *J* = 7.0 Hz, 2H), 7.35–7.27 (m, 4H), 7.23 (d, *J* = 6.4 Hz, 1H), 7.16 (d, *J* = 7.1 Hz, 2H), 5.83 (s, 1H), 4.39 (d, *J* = 7.2 Hz, 2H), 4.28–4.20 (m, 2H), 4.13 (s, 1H), 4.07 (d, *J* = 7.6 Hz, 1.45 H) and 4.01 (s, 0.55 H), 3.32 (d, *J* = 9.1 Hz, 2H), 3.41 (s, 1H) and 3.27 (d, *J* = 9.3 Hz, 5H), 3.15 (d, *J* = 12.6 Hz, 1H), 2.62 (d, *J* = 15.4 Hz, 1H), 1.48 (s, 8H) and 1.43 (s, 1H). ^13^C{^1^H} NMR (100 MHz, CDCl_3_) mixture of rotamers δ 168.9, 168.8, 156.2, 144.0, 141.3, 138.2, 129.1, 128.7, 127.7, 127.1, 126.7, 125.2, 120.0, 103.2, 81.8, 67.2, 64.7, 54.8 and 54.6, 51.5, 47.2, 43.1, 34.8, 28.1 and 28.0. ESI-MS (*m*/*z*): 611.22 (100, [M + Na]^+^). Anal. Calcd for C_34_H_40_N_2_O_7_: C, 69.37; H, 6.85; N, 4.76. Found: 69.18; H, 7.01; N, 5.22.

#### (9*H*-Fluoren-9-yl)methyl (*S*)-4-(1-Methoxy-1-oxo-3-phenylpropan-2-yl)-3-oxopiperazine-1-carboxylate (**4a**)

Compound **4a** was obtained following the general procedure D, using **3a** (0.70 g, 1.28 mmol), and the mixture of TFA (3.50 mL), TIPS (2.80 mL), and 1,2-DCE (0.70 mL). The crude product was purified by flash chromatography (hexane–EtOAc 2:1) to give **4a** (0.53 g) as a white oil in 85% yield. [α]_D_^24^ = −48.6 (*c* 1.0, CHCl_3_). ^1^H NMR (200 MHz, CDCl_3_) mixture of rotamers: δ 7.76 (d, *J* = 7.5 Hz, 2H), 7.52 (d, *J* = 7.2 Hz, 2H), 7.45–7.28 (m, 6H), 7.20 (s, 3H), 5.25 (s, 1H), 4.40 (d, *J* = 5.2 Hz, 2H), 4.23–4.17 (m, *J* = 6.6 Hz, 1H), 4.04 (d, *J* = 2.5 Hz, 2H), 3.76 (s, 3H), 3.42 (d, *J* = 14.3 Hz, 3H), 3.20 (d, *J* = 18.2 Hz, 2H), 3.06 (d, *J* = 14.8 Hz, 1H). ^13^C{^1^H} NMR (50 MHz, CDCl_3_) mixture of rotamers δ 170.6, 165.7, 154.4, 143.7, 141.3, 136.3, 129.3, 128.7 and 128.6, 127.8, 127.1, 124.8, 120.0, 67.9, 58.0, 52.5, 47.3 and 47.1, 44.2, 40.6, 34.3. ESI-MS (*m*/*z*): 507.19 (100, [M + Na]^+^). Anal. Calcd for C_29_H_28_N_2_O_5_: C, 71.88; H, 5.82; N, 5.78. Found: C, 71.64; H, 6.01; N, 5.66.

#### (*S*)-2-(4-(((9*H*-Fluoren-9-yl)methoxy)carbonyl)-2-oxopiperazin-1-yl)-3-phenylpropanoic Acid (**4b**)

Compound **4b** was obtained, as a white spongy solid, following the general procedure D, using **3b** (0.95 g, 1.61 mmol), and the mixture of TFA (4.47 mL), TIPS (3.58 mL), and 1,2-DCE (0.89 mL) in 53% yield (0.40 g). Alternatively, following the general procedure E, using **4a** (0.53 g, 1.09 mmol), dioxane (2.72 mL), and 5 M HCl (2.72 mL), compound **4b** was obtained in 50% yield (0.256 g). [α]_D_^24^ = −49.7 (*c* 1.0, CHCl_3_). ^1^H NMR (200 MHz, CDCl_3_) mixture of rotamers δ 7.76 (d, *J* = 7.4 Hz, 2H), 7.51 (d, *J* = 7.0 Hz, 2H), 7.45–7.29 (m, 6H), 7.20 (s, 3H), 4.99 (s, 1H), 4.42 (s, 2H), 4.20 (s, 1H), 4.09 (s, 2H), 3.62–3.04 (m, 6H). ^13^C{^1^H} NMR (100 MHz, CD_3_OD) mixture of rotamers δ 172.0, 166.5, 154.6, 143.6, 141.1, 137.1, 129.2, 128.5 and 128.3, 127.5 and 127.4, 126.9 and 126.8, 126.5, 124.9 and 124.5, 119.6 and 119.5, 66.8, 59.2, 46.9, 46.6, 44.6, 41.8, 40.6, 33.6. ESI-MS (*m*/*z*): 493.16 (100, [M + Na]^+^). Anal. Calcd for C_28_H_26_N_2_O_5_: C, 71.48; H, 5.57; N, 5.95. Found: C, 71.60; H, 5.61; N, 5.83.

#### (9*H*-Fluoren-9-yl)methyl (*S*)-4-(1-Methoxy-1-oxo-3-phenylpropan-2-yl)-3-oxo-3,4-dihydropyrazine-1(2*H*)-carboxylate (**5a**)

Compound **5a** was obtained as a white oil in 19% yield as a byproduct starting from **3a** when the cyclization reaction was conducted with TES instead of TIPS. [α]_D_^24^ = −42.6 (*c* 1.0, CHCl_3_). ^1^H NMR (400 MHz, CDCl_3_) δ 7.78 (d, *J* = 7.3 Hz, 2H), 7.54 (s, 2H), 7.46–7.30 (m, 4H), 7.21 (dd, *J* = 10.6, 6.7 Hz, 3H), 6.44 (d, *J* = 6.4 Hz, 0.5H) and 6.25 (d, *J* = 6.2 Hz, 0.5H), 5.67–5.56 (m, 1H), 5.36 (dd, *J* = 10.5, 5.5 Hz, 1H), 4.48 (d, *J* = 6.9 Hz, 2H), 4.27 (s, 1H), 4.20–4.13 (m, 2H), 3.77 (s, 3H), 3.39 (dd, *J* = 14.3, 5.5 Hz, 1H), 3.08 (d, *J* = 10.6 Hz, 1H). ^13^C{^1^H} NMR (100 MHz, CDCl_3_) δ 173.0, 167.6, 154.6, 143.3, 141.3, 135.8, 129.8, 128.6, 127.9, 127.2, 124.8, 120.1, 110.0 and 109.4, 96.5, 68.5 and 68.3, 56.4, 52.5, 47.1, 47.0, 46.8, 35.5. ESI-MS (*m*/*z*): 505.32 (100, [M + Na]^+^). Anal. Calcd for C_29_H_26_N_2_O_5_: C, 72.19; H, 5.43; N, 5.81. Found: C, 72.44; H, 5.59; N, 5.69.

#### Methyl (2,2-Dimethoxyethyl)glycinate (**7a**)

Compound **7a** was obtained as a yellow oil in 99% yield, following the general procedure A using glycine methyl ester hydrochloride (1.00 g, 7.96 mmol), MeOH (24 mL), triethylamine (1.11 mL, 7.96 mmol), a 60% aqueous solution of dimethoxyacetaldehyde (1.2 mL, 7.96 mmol), and 10% Pd/C (0.11 g). Spectroscopic data are in agreement with those reported in the literature.^33^

#### *tert*-Butyl (2,2-Dimethoxyethyl)glycinate (**7b**)

Compound **7b** (1.30 g) was obtained as a pale white oil in 99% yield, following the general procedure A using glycine *tert*-butyl ester hydrochloride (1.00 g, 5.97 mmol), MeOH (18 mL), triethylamine (0.90 mL, 5.97 mmol), a 60% aqueous solution of dimethoxyacetaldehyde (0.90 mL, 5.97 mmol), and 10% Pd/C (0.083 g). Spectroscopic data are in agreement with those reported in the literature.^34^

#### Methyl *N*-((((9*H*-Fluoren-9-yl)methoxy)carbonyl)-l-phenylalanyl)-*N*-(2,2-dimethoxyethyl)glycinate (**8a**)

Compound **8a** was obtained following the general procedure C using **7a** (0.500 g, 2.82 mmol), Fmoc-l-phenylalanine hydrochloride (1.15 g, 2.82), DIPEA (0.98 mL, 5.64 mmol), and CH_2_Cl_2_ (14.1 mL). The crude product was purified by flash chromatography (hexane–Et_2_O 1:1) to give **8a** (0.92 g) as a white spongy solid in 60% yield. [α]_D_^24^ = −23.4 (*c* 1.0, CHCl_3_). ^1^H NMR (400 MHz, CDCl_3_) mixture of rotamers δ 7.76 (d, *J* = 7.5 Hz, 2H), 7.55 (dd, *J* = 11.5, 5.3 Hz, 2H), 7.39 (t, *J* = 7.4 Hz, 2H), 7.34–7.28 (m, 4H), 7.26–7.23 (m, 2H), 7.20 (d, *J* = 7.3 Hz, 1H), 5.63 (t, *J* = 8.7 Hz, 1H), 4.99 (dd, *J* = 15.7, 7.0 Hz, 0.6H) and 4.67 (dd, *J* = 15.3, 7.0 Hz, 0.4H), 4.39–4.33 (m, *J* = 10.5, 3.2 Hz, 1.20H) and 4.29–4.25 (m, 0.80H), 4.22 (d, *J* = 10.2 Hz, 0.8H) 4.21–4.14 (m, 2H), 4.04 (d, *J* = 17.2 Hz, 0.6H), 3.89 (dd, *J* = 21.8, 8.4 Hz, 0.4H), 3.72 (d, *J* = 3.1 Hz, 3H), 3.54 (dd, *J* = 14.0, 5.2 Hz, 0.4H), 3.44–3.36 (m, 1.20H), 3.35–3.29 (m, 6H), 3.27 (d, *J* = 4.9 Hz, 0.4H), 3.17–3.06 (m, 1H), 2.98 (dd, *J* = 13.6, 6.5 Hz, 1H). ^13^C{^1^H} NMR (100 MHz, CDCl_3_) mixture of rotamers 172.6, 169.4, 155.5, 143.8, 141.2, 136.2, 129.6 and 129.4, 128.6, 128.5, 127.7, 127.0, 125.2 and 125.1, 119.9, 103.7 and 103.4, 67.0, 55.2 and 54.9, 52.5 and 52.0, 52.2 and 52.1, 51.0 and 50.5, 49.2, 47.1, 39.5. ESI-MS (*m*/*z*): 569.26 (100, [M + Na]^+^). Anal. Calcd for C_31_H_34_N_2_O_7_: C, 68.12; H, 6.27; N, 5.12. Found: C, 68.29; H, 6.31; N, 5.02.

#### *tert*-Butyl *N*-((((9*H*-Fluoren-9-yl)methoxy)carbonyl)-l-phenylalanyl)-*N*-(2,2-dimethoxyethyl)glycinate (**8b**)

Compound **8b** was obtained following the general procedure C using **7b** (0.50 g, 2.28 mmol), Fmoc-l-phenylalanine hydrochloride (0.92 g, 2.28 mmol), DIPEA (0.81 mL, 4.56 mmol), and CH_2_Cl_2_ (22.8 mL). The crude product was purified by flash chromatography (hexane–Et_2_O 1:1) to give **8b** (0.98 g) as a white spongy solid in 73% yield. [α]_D_^24^ = −8.72 (*c* 1.0, CHCl_3_). ^1^H NMR (400 MHz, CDCl_3_) mixture of rotamers δ 7.76 (d, *J* = 7.5 Hz, 2H), 7.54 (dd, *J* = 12.3, 5.6 Hz, 2H), 7.42–7.37 (m, *J* = 7.4 Hz, 2H), 7.29 (dd, *J* = 13.7, 5.7 Hz, 4H), 7.24 (d, *J* = 6.8 Hz, 2H), 7.20 (d, *J* = 7.1 Hz, 1H), 5.60–5.55 (m, 1H), 4.99 (m, 0.6H) and 4.69–4.63 (m, 0.4H), 4.39–4.34 (m, 1.20H) and 4.27–4.22 (m, 0.80H), 4.22–4.18 (m, 1H), 4.17–4.10 (m, 2H), 4.00–3.94 (m, *J* = 17.1 Hz, 0.6H), 3.86 (d, *J* = 18.7 Hz, 0.6H), 3.49–3.40 (m, 1.2H) 3.58–3.52 (m, 0.6H), 3.38–3.30 (m, 6H), 3.20–3.08 (m, 1H), 3.00–2.92 (m, 1H), 1.47 (s, 9H). ^13^C{^1^H} NMR (100 MHz, CDCl_3_) mixture of rotamers δ 172.6, 169.7, 155.5, 143.8, 141.2, 136.3, 129.6 and 129.4, 128.5 and 128.4, 127.7, 127.0 and 126.9, 125.2 and 125.1, 120.1, 103.9 and 103.5, 81.7, 67.0, 55.2 and 55.0, 52.1 and 51.9, 51.4, 50.0 and 49.9, 47.1, 39.3, 28.1 and 28.0. ESI-MS (*m*/*z*): 611.32 (100, [M + Na]^+^). Anal. Calcd for C_34_H_40_N_2_O_7_: C, 69.37; H, 6.85; N, 4.76. Found: C, 69.52; H, 6.91; N, 4.81.

#### (9*H*-Fluoren-9-yl)methyl (*S*)-2-Benzyl-4-(2-methoxy-2-oxoethyl)-3-oxopiperazine-1-carboxylate (**9a**)

Compound **9a** was obtained following the general procedure D using **8a** (0.200 g, 0.36 mmol) and a mixture of TFA (1.0 mL), TIPS (0.80 mL), and 1,2-DCE (0.20 mL). The crude product was purified by flash chromatography (hexane–EtOAc 2:1) to give **9a** (0.13 g) as a white oil in 75% yield. [α]_D_^24^ = −47.6 (*c* 1.0, CHCl_3_). ^1^H NMR (400 MHz, CDCl_3_) mixture of rotamers: δ 7.75 (d, *J* = 7.0 Hz, 2H), 7.57–7.45 (m, 2H), 7.38 (d, *J* = 5.9 Hz, 2H), 7.36–7.21 (m, 4H), 7.12 (d, *J* = 4.4 Hz, 2H), 6.96 (d, *J* = 3.8 Hz, 1H), 5.05–4.79 (m, 0.4H) and 4.53 (s, 0.6H), 4.60 (d, *J* = 5.6 Hz, 0.3H) and 4.35 (t, *J* = 10.8 Hz, 1.70H), 4.22 (d, *J* = 5.4 Hz, 0.6H) and 4.03 (s, 0.4), 4.17 (s, 0.6H) and 4.01 (s, 0.4H), 3.99 (s, 1H) 3.74 (s, 3H), 3.50–3.38 (m, 1H), 3.29 (d, *J* = 14.8 Hz, 1H), 3.17–3.06 (m, 1H), 2.94 (d, *J* = 11.4 Hz, 1H), 2.88–2.76 (m, 1H), 2.72–2.64 (m, 1H). ^13^C{^1^H} NMR (100 MHz, CDCl_3_) mixture of rotamers δ 168.9, 167.7, 154.6, 143.9, 141.3, 136.9, 129.8, 128.4 and 126.8, 127.8, 127.1, 124.8, 124.6, 120.1, 67.2, 58.5 and 58.1, 52.3, 48.6 and 48.4, 47.6 and 47.5, 47.2, 39.1 and 38.1. ESI-MS (*m*/*z*): 507.20 (100, [M + Na]^+^). Anal. Calcd for C_29_H_28_N_2_O_5_: C, 71.88; H, 5.82; N, 5.78. Found: C, 71.52; H, 6.26, N, 5.56.

#### (*S*)-2-(4-(((9*H*-Fluoren-9-yl)methoxy)carbonyl)-3-benzyl-2-oxopiperazin-1-yl)acetic Acid (**9b**)

Compound **9b** was obtained, as a white spongy solid, following the general procedure D, using **8b** (0.500 g, 0.85 mmol), and a mixture of TFA (2.36 mL), TIPS (1.11 mL), and 1,2-DCE (0.47 mL) in 48% yield (0.67 g). Alternatively, following the general procedure E, compound **9b** was obtained from **9a** (0.13 g, 0.27 mmol), dioxane (0.68 mL), and 5 M HCl (0.68 mL) in 52% yield (0.066 g). [α]_D_^24^ = −42.9 (*c* 1.0, CHCl_3_). ^1^H NMR (400 MHz, CDCl_3_) mixture of rotamers δ 7.74 (d, *J* = 6.6 Hz, 2H), 7.49 (dd, *J* = 32.8, 6.9 Hz, 2H), 7.39 (d, *J* = 6.9 Hz, 2H), 7.32 (d, *J* = 5.8 Hz, 4H), 7.19–7.05 (m, 2H), 6.95 (s, 1H), 4.92 (s, 0.30H) and 4.55 (s, 0.70H), 4.34 (s, 2H), 4.29–4.19 (m, 1H), 4.20–4.09 (m, 1H), 4.00 (s, 1H), 3.67–3.63 (m, 0.40H) and 3.49–3.45 (m, 0.60H), 3.29–3.12 (m, 1H), 3.07 (dd, *J* = 13.8, 5.9 Hz, 1H), 2.98–2.89 (m, 1H), 2.88–2.82 (m, 1H), 2.71 (d, *J* = 10.6 Hz, 1H), 2.73–2.55 (m, 1H). ^13^C{^1^H} NMR (100 MHz, CDCl_3_) mixture of rotamers δ 171.9, 169.8 and 168.6, 154.8 and 154.3, 143.8 and 143.3, 141.3, 136.6, 129.8, 128.4 and 126.9, 127.8, 127.2, 127.1, 124.8, 124.6, 120.1, 67.5, 58.4, 48.7, 47.7, 47.2, 38.9 and 38.0, 37.1 and 36.84. ESI-MS (*m*/*z*): 503.56 (100, [M + Na]^+^). Anal. Calcd for C_28_H_26_N_2_O_5_: C, 71.48; H, 5.57; N, 5.95. Found: C, 71.61; H, 5.61; N, 5.84.

#### Methyl *N*-(3-((((9*H*-Fluoren-9-yl)methoxy)carbonyl)amino)propanoyl)-*N*-(2,2-dimethoxyethyl)-l-phenylalaninate (**10**)

Compound **10a** was obtained following the general procedure **B** using **2a** (1.00 g, 3.74 mmol), Fmoc-β-ala-OH (1.28 g, 4.11 mmol), HATU (2.13 g, 5.61 mmol), dry DIPEA (0.130 mL, 7.48 mmol), and dry DMF (20 mL). The crude product was purified by flash chromatography (hexane–EtOAc 1:1), to give **10** (1.10 g) as a white oil in 52% yield. [α]_D_^24^ = −76.4 (*c* 1.0, CHCl_3_). ^1^H NMR (400 MHz, CDCl_3_) mixture of rotamers δ 7.76 (d, *J* = 7.5 Hz, 2H), 7.61 (d, *J* = 7.4 Hz, 2H), 7.44–7.35 (m, 2H), 7.34–7.26 (m, 4H), 7.25–7.19 (m, 2H), 7.14 (d, *J* = 7.6 Hz, 2H), 5.60–5.54 (m, 1H), 4.39 (d, *J* = 7.0 Hz, 1.70H) and 4.35 (d, *J* = 6.6 Hz, 0.30H), 4.26–4.23 (m, 1H), 4.23–4.19 (m, 1H), 4.19–4.13 (m, 1H), 3.73 (s, 3H), 3.54–3.47 (m, 2H), 3.41 (d, *J* = 8.1 Hz, 1H) and 3.28 (d, *J* = 13.7 Hz, 5H), 3.38–3.32 (m, 2H), 3.20 (d, *J* = 5.1 Hz, 1H), 2.66–2.63 (m, 1H) and 2.58–2.47 (m, 1.70H), 2.61 (d, *J* = 4.6 Hz, 1H). ^13^C{^1^H} NMR (100 MHz, CDCl_3_) mixture of rotamers δ 174.4, 170.8, 156.4, 144.0, 141.3, 137.9, 129.1, 128.6, 127.6, 127.0, 126.7, 125.1, 119.9, 103.6, 66.7, 63.4, 55.2 and 54.6, 52.3, 52.3 and 52.1, 47.2, 36.7, 34.7. ESI-MS (*m*/*z*): 583.24 (100, [M + Na]^+^). Anal. Calcd for C_32_H_36_N_2_O_7_: C, 68.56; H, 6.47; N, 5.00. Found: C, 68.72; H, 6.59; N, 5.04.

#### (9*H*-Fluoren-9-yl)methyl (*S*)-4-(1-Methoxy-1-oxo-3-phenylpropan-2-yl)-5-oxo-1,4-diazepane-1-carboxylate (**11a**)

Compound **11a** was obtained following the general procedure **D** using **10** (1.10 g, 1.96 mmol) and a mixture of TFA (5.40 mL), TIPS (4.35 mL), and 1,2-DCE (1.08 mL). The crude product was purified by flash chromatography (hexane–EtOAc 1:1) to give **11a** (0.750 g) as a white oil in 76% yield. [α]_D_^24^ = −58.4 (*c* 1.0, CHCl_3_). ^1^H NMR (400 MHz, CDCl_3_) mixture of rotamers δ 7.74 (d, *J* = 7.5 Hz, 2H), 7.51 (d, *J* = 7.4 Hz, 2H), 7.42–7.35 (m, 2H), 7.34–7.29 (m, 4H), 7.26 (s, 1H), 7.17 (d, *J* = 7.0 Hz, 2H), 5.17 (s, 1H), 4.52 (d, *J* = 5.9 Hz, 2H), 4.19 (t, *J* = 5.8 Hz, 1H), 3.73 (s, 3H), 3.41 (d, *J* = 5.2 Hz, 1H), 3.37 (d, *J* = 5.3 Hz, 1H), 3.29 (s, 1H), 3.27–3.22 (m, 1H), 3.16 (s, 1H), 3.09 (d, *J* = 9.9 Hz, 1H), 3.05 (d, *J* = 3.5 Hz, 1H), 3.03–2.89 (m, 1H), 2.57–2.10 (m, 2H). ^13^C{^1^H} NMR (50 MHz, CDCl_3_) mixture of rotamers δ 174.4, 171.3, 155, 143.8, 141.4, 136.8, 128.8, 128.7, 127.8, 127.1, 127.0, 124.8, 120.0, 67.0, 60.2, 52.4, 48.9, 47.4, 46.8, 41.6, 39.1, 35.1. ESI-MS (*m*/*z*): 521.31 (100, [M + Na]^+^). Anal. Calcd for C_30_H_30_N_2_O_5_: C, 72.27; H, 6.07; N, 5.62. Found: C, 72.41; H, 6.13; N, 5.54.

#### (*S*)-2-(4-(((9*H*-Fluoren-9-yl)methoxy)carbonyl)-7-oxo-1,4-diazepan-1-yl)-3-phenylpropanoic Acid (**11b**)

Compound **11b** (0.320 g) was obtained following the general procedure E using **11a** (0.750 g, 1.50 mmol), dioxane (3.75 mL), and 5 M HCl (3.75 mL) as a spongy white solid in 44% yield. [α]_D_^24^ = −59.4 (*c* 1.0, CHCl_3_). ^1^H NMR (400 MHz, CDCl_3_) mixture of rotamers δ 7.73 (d, *J* = 6.4 Hz, 2H), 7.50 (d, *J* = 7.3 Hz, 2H), 7.41–7.35 (m, *J* = 7.2 Hz, 2H), 7.30 (d, *J* = 6.6 Hz, 5H), 7.17 (d, *J* = 7.3 Hz, 2H), 4.91 (s, 1H), 4.51 (s, 2H), 4.18 (s, 1H), 3.39 (d, *J* = 10.6 Hz, 2H), 3.33–2.84 (m, 6H), 2.50–2.18 (m, 2H). ^13^C{^1^H} NMR (50 MHz, CDCl_3_) mixture of rotamers δ 174.0, 170.0, 155.0, 143.7, 141.4, 136.9, 128.9, 128.7, 127.8, 127.1, 124.7, 120.0, 67.1, 61.8, 50.0, 47.4, 46.6, 41.5, 39.0, 34.8. ESI-MS (*m*/*z*): 507.22 (100, [M + Na]^+^). Anal. Calcd for C_29_H_28_N_2_O_5_: C, 71.88; H, 5.82; N, 5.78. Found: C, 71.99; H, 5.96; N, 5.68.

#### (9*H*-Fluoren-9-yl)methyl 4-((*S*)-1-(((*S*)-1-Methoxy-3-methyl-1-oxobutan-2-yl)amino)-1-oxo-3-phenylpropan-2-yl)-3-oxopiperazine-1-carboxylate (**12**)

Compound **12** was obtained following the general procedure F, using **4b** (0.250 g, 0.53 mmol), COMU (0.342 g, 1.5 mmol), l-valine methyl ester hydrochloride (0.090 g; 0.53 mmol), dry DIPEA (0.55 mL, 3.19 mmol), and dry DMF (21.2 mL). The crude product was purified by flash chromatography (hexane–EtOAc 1:1) to give **12** (0.103 g) as a white oil in 40% yield. [α]_D_^24^ = −62.3 (*c* 1.0, CHCl_3_). ^1^H NMR (400 MHz, CDCl_3_) δ 7.76 (d, *J* = 7.6 Hz, 2H), 7.51 (s, 2H), 7.43–7.37 (m, 2H), 7.29 (dd, *J* = 14.4, 7.2 Hz, 4H), 7.24–7.18 (m, 3H), 6.62 (d, *J* = 8.5 Hz, 1H), 5.34 (s, 1H), 4.42 (d, *J* = 23.3 Hz, 3H), 4.20 (d, *J* = 6.6 Hz, 1H), 4.13 (s, 1.30H) and 4.04 (s, 0.70), 4.00 (s, 0.30H) and 3.46–3.21 (m, 4.70H), 3.70 (s, 3H), 3.08 (s, 1H), 2.19–2.10 (m, 1H), 0.85 (dd, *J* = 14.2, 6.7 Hz, 6H). ^13^C{^1^H} NMR (100 MHz, CDCl_3_) δ 170.3, 169.2, 166.7, 154.7, 143.6, 141.3, 136.1, 128.8, 128.7, 127.8, 127.1, 127.0, 120.1, 68.0, 57.1, 52.2, 47.6, 47.1, 42.8, 38.6, 33.4, 31.0, 19.0, 17.6. ESI-MS (*m*/*z*): 606.23 (100, [M + Na]^+^). Anal. Calcd for C_34_H_37_N_3_O_6_: C, 69.96; H, 6.39; N, 7.20. Found: C, 70.08; H, 6.45; N, 7.05.

#### (9*H*-Fluoren-9-yl)methyl (*S*)-2-Benzyl-4-(2-(((*S*)-1-methoxy-3-methyl-1-oxobutan-2-yl)amino)-2-oxoethyl)-3-oxopiperazine-1-carboxylate (**13**)

Compound **13** was obtained following the general procedure F, using **9b** (0.250 g, 0.53 mmol), COMU (0.342 g, 1.5 mmol), l-valine methyl ester hydrochloride (0.090 g; 0.53 mmol), dry DIPEA (0.55 mL, 3.19 mmol), and dry DMF (21.2 mL). The crude product was purified by flash chromatography (hexane–EtOAc 1:2) to give **13** as a white spongy solid (0.117 g) in 38% yield. [α]_D_^24^ = −53.7 (*c* 1.0, CHCl_3_). ^1^H NMR (400 MHz, CDCl_3_) mixture of rotamers δ 7.75 (d, *J* = 7.1 Hz, 2H), 7.53–7.43 (m, 2H), 7.40 (dd, *J* = 16.6, 9.3 Hz, 2H), 7.35–7.18 (m, 4H), 7.14 (s, 2H), 6.99 (s, 1H), 6.76 (s, 1H), 4.92 (s, 0.4H) and 4.54 (s, 0.6H), 4.54–4.51 (m, 0.3H) and 4.34 (d, *J* = 11.1 Hz, 1.70H), 4.51–4.44 (m, 1H), 4.22 (s, 0.6H) and 4.05 (s, 0.4H), 4.15 (s, 0.4H) and 3.94 (s, 0.6H), 3.73 (s, 3H), 3.64 (s, 1H), 3.47 (s, 2H), 3.32–3.22 (m, 1H), 3.10 (d, *J* = 9.5 Hz, 1H), 2.98–2.84 (m, 2H), 2.24–2.14 (m, 1H), 0.97–0.87 (m, 6H). ^13^C{^1^H} NMR (100 MHz, CDCl_3_) mixture of rotamers δ 172.1, 170.3, 166.1, 154.6, 146.4, 141.3, 138.5, 129.6, 128.4, 127.7, 127.1, 124.8, 120.0, 67.3, 58.8, 57.2, 52.2, 51.5, 47.9, 47.2, 36.9, 30.9, 19.0, 17.7.ESI-MS (*m*/*z*): 606.24 (100, [M + Na]^+^). Anal. Calcd for C_34_H_37_N_3_O_6_: C, 69.96; H, 6.39; N, 7.20. Found: C, 69.56; H, 6.61; N, 6.96.

#### (9*H*-Fluoren-9-yl)methyl 4-((*S*)-1-(((*S*)-1-Methoxy-3-methyl-1-oxobutan-2-yl)amino)-1-oxo-3-phenylpropan-2-yl)-5-oxo-1,4-diazepane-1-carboxylate (**14**)

Compound **14** was obtained following the general procedure F, using **11b** (0.100 g, 0.206 mmol), l-valine methyl ester hydrochloride (0.035 g, 0.206 mmol), dry DIPEA (0.22 mL, 1,24 mmol), and dry DMF (8.24 mL). The crude product was purified by flash chromatography (hexane–EtOAc 1:2) to give **14** (0.103 g) as a white spongy solid in 33% yield. [α]_D_^24^ = −49.5 (*c* 1.0, CHCl_3_). ^1^H NMR (400 MHz, CDCl_3_) mixture of rotamers δ 7.73 (d, *J* = 7.4 Hz, 2H), 7.51 (d, *J* = 6.6 Hz, 2H), 7.42–7.34 (m, 2H), 7.30 (s, 4H), 7.24–7.21 (m, 1H), 7.18 (d, *J* = 7.1 Hz, 2H), 6.58 (s, 1H), 5.45–5.26 (m, 1H), 4.54–4.49 (m, 2H), 4.45 (d, *J* = 3.6 Hz, 1H), 4.18 (t, *J* = 5.6 Hz, 1H), 3.69 (d, *J* = 7.9 Hz, 3H), 3.45–3.06 (m, 8H), 3.02 (d, *J* = 14.9 Hz, 1H), 2.19–2.08 (m, 1H), 0.88 (d, *J* = 7.0 Hz, 3H), 0.85 (d, *J* = 6.9 Hz, 2H). ^13^C{^1^H} NMR (100 MHz, CDCl_3_): δ 171.7, 170.1, 158.1, 154.9, 143.7, 141.3, 136.3, 128.8, 128.7, 127.7, 127.0, 127.0, 124.7, 120.0, 67.0, 57.1, 52.2, 47.4, 47.1, 41.6, 39.1, 34.1, 30.3, 19.1, 17.7. ESI-MS (*m*/*z*): 620.33 (100, [M + Na]^+^). Anal. Calcd for C_35_H_39_N_3_O_6_: C, 70.33; H, 6.58; N, 7.03. Found: C, 70.58; H, 6.65; N, 7.00.

#### General Procedure (G) for Peptide Synthesis and HPLC Purification and Analysis

The peptides were synthesized on a ChemMatrix Rink Amide resin, with a 0.5 mmol/g loading capacity and bead size of 100–200 mesh. Peptide cleavage from the resin was achieved under acidic conditions. The resin was swelled in dichloromethane for 20 min, under magnetic stirring prior to peptide synthesis. Then, the solution was filtered and washed twice with DMF. Peptide coupling was carried out using 5 equiv Fmoc-amino acids as 0.2 M DMF solution and 10 equiv DIC/Oxyma, both 1 M in DMF as the activating mixture by heating at 90 °C for 3 min under microwave irradiation, followed by DMF washings (3×). 20% Morpholine was used as Fmoc deblocking agent by heating at 90 °C for 2 min (2×), each cycle followed by DMF washings (3×). Final resin washing before acidic cleavage was carried out with DMF (3×) and CH_2_Cl_2_ (2×). A mixture of 2.5% H_2_O and 2.5% TIPS in TFA was added into the peptidyl resin, and the suspension was shaken for 2 h followed by filtration. Cold diethyl ether (10 mL) was added to the filtrate, and the peptide was precipitated. The mixture was centrifuged for 3 min at 2500 rpm, and the ether layer was separated. The procedure was repeated three times, then a second treatment with TFA containing water and TIPS was repeated for 15 min, followed by peptide precipitation with cold ether. Then, the peptide was dried under vacuum overnight to give the crude peptide. Peptides were analyzed and purified using Dionex Ultimate 3000 system equipped with a reversed-phase analytical column Synergi 4 μm Fusion-RP 80 Å (150 × 4.6 mm^2^) or semipreparative column Synergi 10 μm Fusion-RP 80 Å (250 × 10.0 mm^2^) and using acetonitrile (0.1% TFA) in H_2_O (0.1% TFA) at room temperature, 5–95% linear gradient over 20 min for analytical and semipreparative runs. Flow rates of 1 and 5 mL/min were used for analytical and semipreparative runs, respectively, and peak detection was achieved at 223 nm. All crude peptides were obtained in >95% purity. The molecular weight of all peptides was confirmed by electrospray mass spectrometry. Analytical samples were prepared as 1 mg/mL conc. by dissolving the dry peptides in 0.1% H_2_O/HCOOH (v/v).

#### H-ED(4b)YQ-NH_2_ (**15**)

Peptide **15** was obtained as a white amorphous solid following general procedure **G**, using Fmoc-Gln(Trt)-OH (0.153 g, 0.25 mmol), Fmoc-Tyr(*t*Bu)-OH (0.115 g, 0.25 mmol), **4b** (0.118 g, 0.25 mmol), Fmoc-Asp(O*t*Bu)-OH (0.103 g, 0.25 mmol), and Fmoc-Glu(O*t*Bu)-OH (0.106 g, 0.25 mmol) in 24% yield, after HPLC purification using the semipreparative column Synergi 10 μm Fusion-RP 80 Å. ESI-MS (*m*/*z*): 783.12 (100, [M + Na]^+^). HPLC (Synergi 4 μm Fusion-RP, water/acetonitrile = 90/10 to 10/90, 25 min, flow rate 1.0 mL/min, λ = 223 nm) *t*_R_ = 9.31 min (purity: 100%).

#### H-ED(11b)YQ-NH_2_ (**16**)

Peptide **16** was obtained as a white amorphous solid following general procedure G, Fmoc-Gln(Trt)-OH (0.153 g, 0.25 mmol), Fmoc-Tyr(*t*Bu)-OH (0.115 g, 0.25 mmol), **11b** (0.121 g, 0.25 mmol), Fmoc-Asp(O*t*Bu)-OH (0.103 g, 0.25 mmol), and Fmoc-Glu(O*t*Bu)-OH (0.106 g, 0.25 mmol) in 30% yield, after HPLC purification using the semipreparative column Synergi 10 μm Fusion-RP 80 Å. ESI-MS (*m*/*z*): 797.12 (100, [M + Na]^+^). HPLC (Synergi 4 μm Fusion-RP, water/acetonitrile = 90/10 to 10/90, 25 min, flow rate 1.0 mL/min, λ = 223 nm) *t*_R_ = 9.30 min (purity: 100%).

## SARS-CoV-2 spike/ACE2 Inhibitor Screening Assays

The synthesized peptides were screened for inhibition of the SARS-CoV-2 spike/ACE2 complex formation using a commercially available SARS-CoV-2 spike inhibitor screening assay kit (AdipoGen Life Sciences, Inc., San Diego) based on the colorimetric ELISA assay, which measures the binding of the RBD of the Spike S protein from SARS-CoV-2 to its human receptor ACE2. All of the measurements were performed in triplicate in 96-well plates following the manufacturer’s instructions. Briefly, the wells were coated by adding 100 μL/well of diluted spike(1 μg/mL) to the 96-well ELISA microplate. After leaving the covered plate overnight at 4 °C, the liquid was removed by the wells by inverting the plate and blotting it against clean absorbent paper. Then, the plate was blocked by adding 200 μL of blocking buffer for 2 h at room temperature. Following liquid removal and washing of coated wells, 100 μL/well of diluted compounds to be tested were added to the wells and the plate was covered and incubated for 1 h at 37 °C. Inhibitory control ACE2 (human) mAb (100 μL/well) was used as a positive control. Then, 100 μL/well of diluted horseradish peroxidase (HRP) labeled streptavidin was added and the plate was covered and incubated again for 1 h at room temperature. After liquid removal and subsequent washings, substrate development was conducted by addition of 100 μL of ready-to-use TMB to each well for 5 min at room temperature, then the reaction was stopped by adding 50 μL of stop solution, and the OD values were measured at 450 nm using a BMG Labtech Fluostar Optima microplate reader and the collected data were analyzed using GraphPad 5.0 Software Package (GraphPad Prism, San Diego, CA). All of the compounds were screened for inhibition at a single concentration (100 μM) in phosphate-buffered saline (PBS), and the IC_50_ value of the active compounds was obtained by dose–response measurements using inhibitor range of concentration 1 μM to 1 mM.

## 3CLPro SARS-CoV-2 Inhibitor Screening Assays

The synthesized peptides were assayed through a fluorometric assay using the fluorogenic substrate Hilyte Fluor-488-ESATLQSGLRKAK-(QXL-520)-NH_2_ (Anaspec). All of the measurements were performed in 96-well plates with a Fluostar Optima microplate reader (BMG Labtech, Ortenberg, Germany). Excitation and emission wavelengths were 490 and 520 nm, respectively. All incubations were performed at 30 °C in 25 mmol of *N*-(2-hydroxyethyl)piperazine-*N*′-ethanesulfonic acid (HEPES), 0.2% Tween-20 at pH 7.0. The inhibitors were preincubated with enzymes (60 nM) for 10 min at 30 °C before the reaction was started by the addition of the fluorogenic substrate (1 μM). The decrease of fluorescence was monitored over 30 min (λ_ex_ = 490 nm, λ_em_ = 520 nm) at 30 °C. The percentages of inhibition for the test compounds were determined through the equation (1 – *V*_s_/*V*_o_) × 100, where *V*_s_ is the initial velocity in the presence of the inhibitor and *V*_0_ is the initial velocity of the uninhibited reaction. The IC_50_ values were obtained by dose–response measurements using an inhibitor range of concentrations 1 μM to 1 mM. All of the experiments were performed in triplicate, and data collected were analyzed using GraphPad 5.0 Software Package (GraphPad Prism, Inc., San Diego, CA).

## Supporting Information Available

Copies of ^1^H and ^13^C NMR spectra for **2**–**14**, HPLC chromatograms of **15** and **16**, copies of ^1^H-ES and TOCSY-ES of **16**, and dose–response measurement of inhibition of **15** and **16** (PDF)

## Footnotes

a An initial version of this work was deposited in ChemRxiv on April 18th 2022, reference DOI: 10.26434/chemrxiv-2022-82s0k.
