# Supplementary material for: Peptidomimetic Small-Molecule Inhibitors of 3CLPro Activity and Spike–ACE2 Interaction: Toward Dual-Action Molecules against Coronavirus Infections
Source: J Org Chem. 2022 Aug 30;87(18):12041–51. doi: 10.1021/acs.joc.2c01047 (PMC9454270; doi:10.1021/acs.joc.2c01047)
Supplement: Supplementary file 1 — jo2c01047_si_001.pdf [file jo2c01047_si_001.pdf]

Peptidomimetic small-molecule inhibitors of 3CLPro activity and Spike-ACE2 interaction: towards dual-action molecules against Coronavirus infections

Filomena Tedesco, Lorenzo Calugi, Elena Lenci, Andrea Trabocchi*

Department of Chemistry "Ugo Schiff", University of Florence, via della Lastruccia 13, 50019 Sesto Fiorentino, Florence, Italy

Supporting Information

Copies of ^{1}H and ^{13}C NMR for **2-14** S2-S19

Figure S1: HPLC chromatogram of **15** S20

Figure S2: HPLC chromatogram of **16** S21

Figure S3: 1H-ES of **16** S22

Figure S4: TOCSY-ES of **16** S23

Figure S5: Inhibition curve of **15** on 3CLPro enzyme S24

Figure S6: Inhibition curve of **16** on 3CLPro enzyme S24

Figure S7: Inhibition curve of **16** on Spike-ACE2 interaction S24

^{1}H NMR (400 MHz, $CDCl_3$)

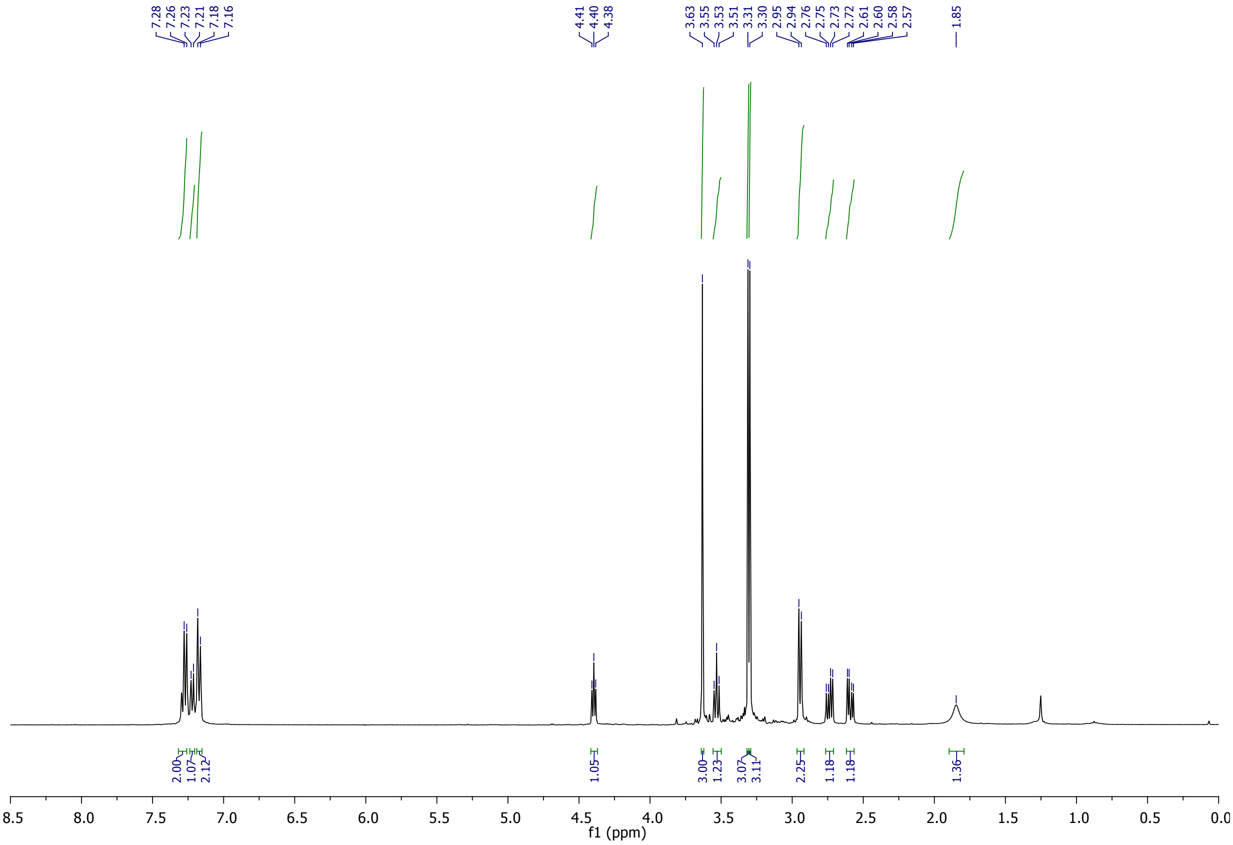

^{13}C{^{1}H} NMR (100 MHz, $CDCl_3$)

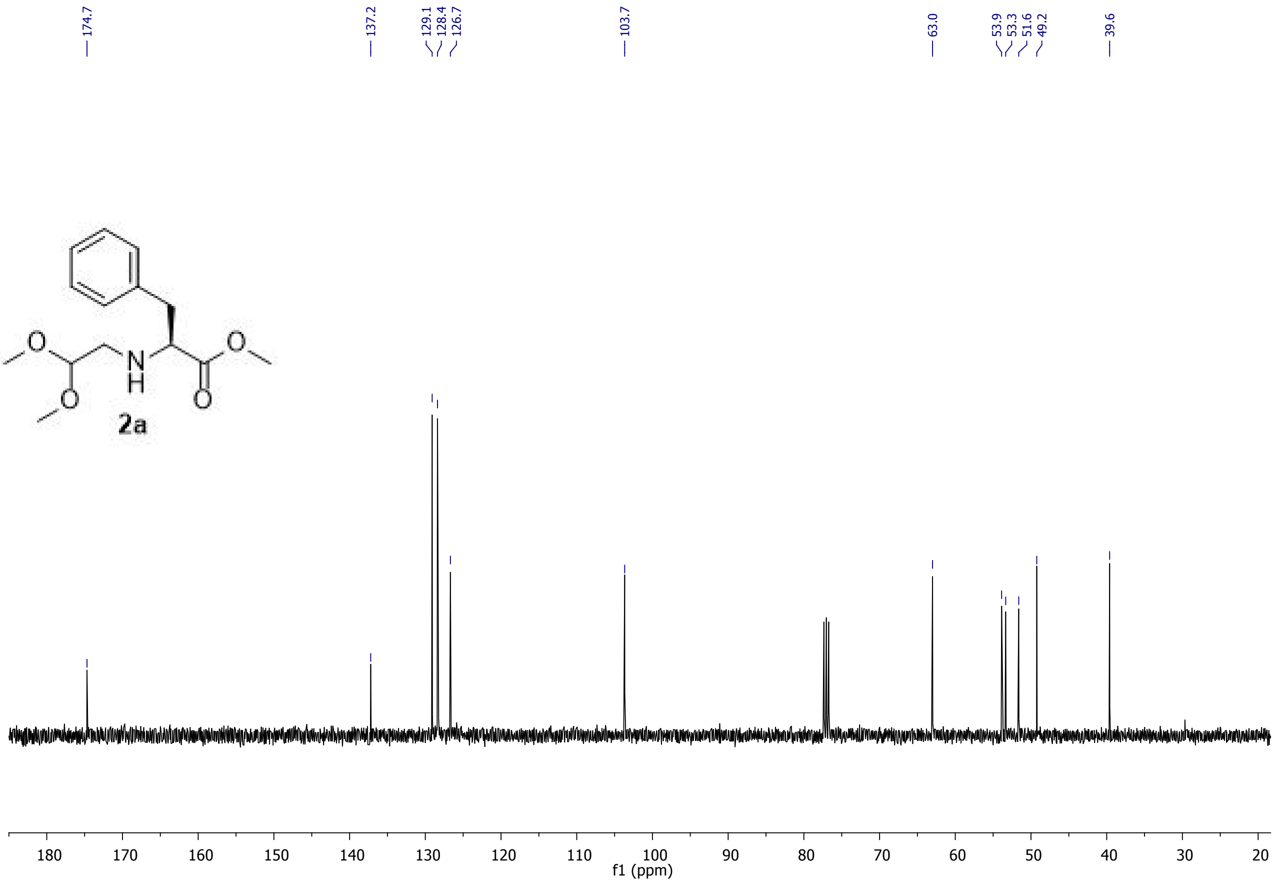

2a

^{1}H NMR (400 MHz, $CDCl_3$)

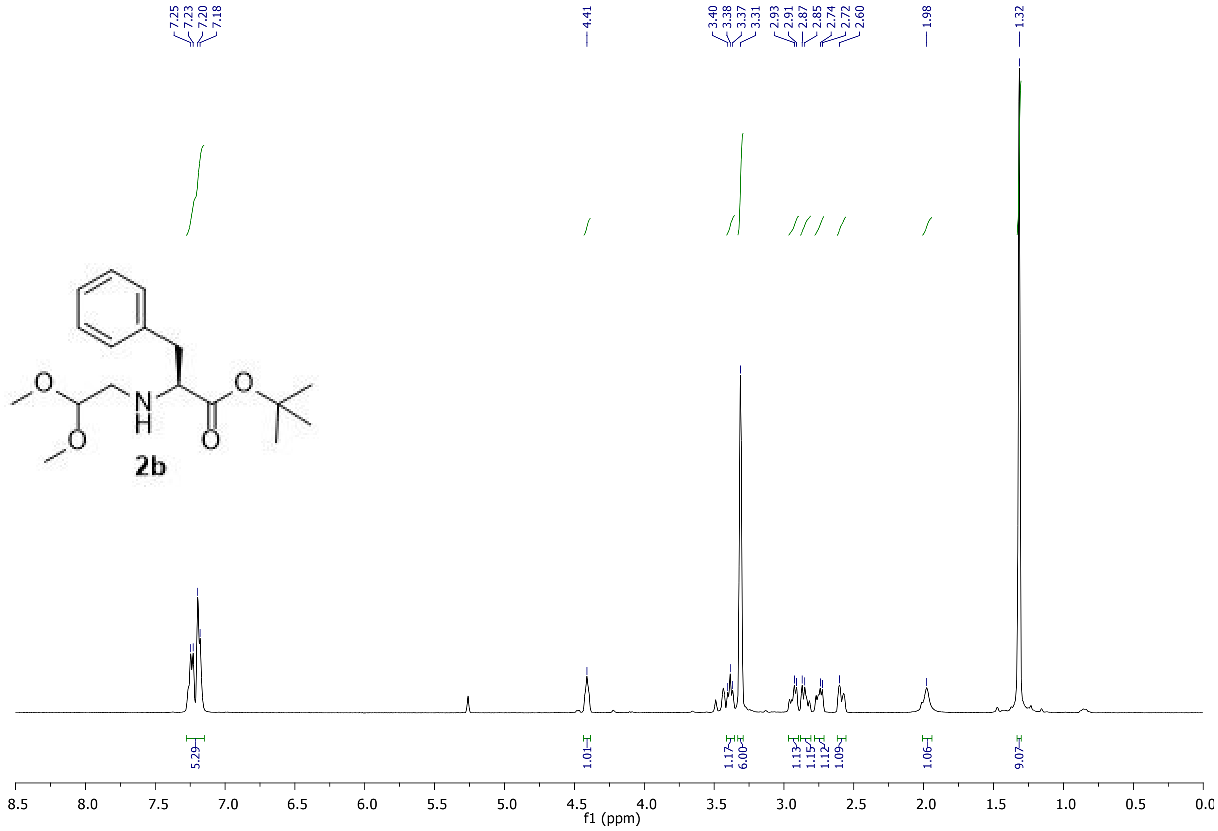

^{13}C{^{1}H} NMR (100 MHz, $CDCl_3$)

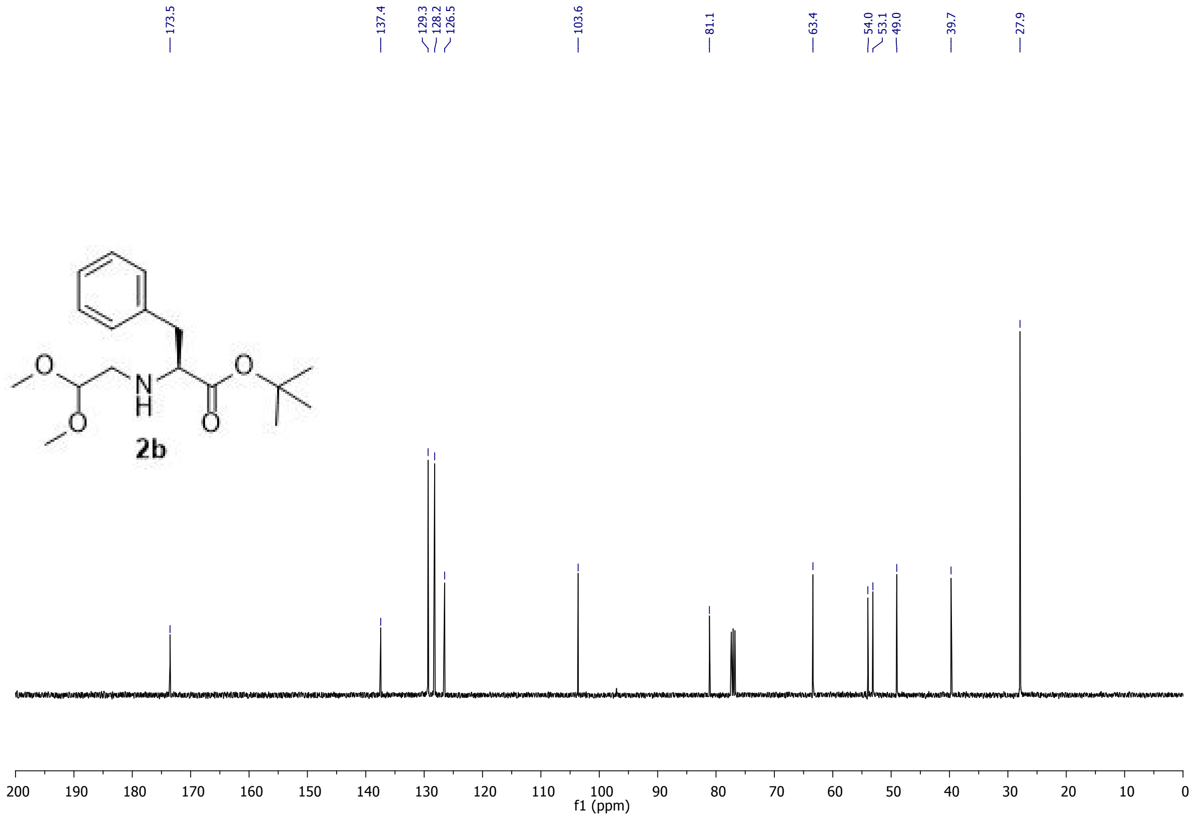

^1H NMR (200 MHz, $CDCl_3$)

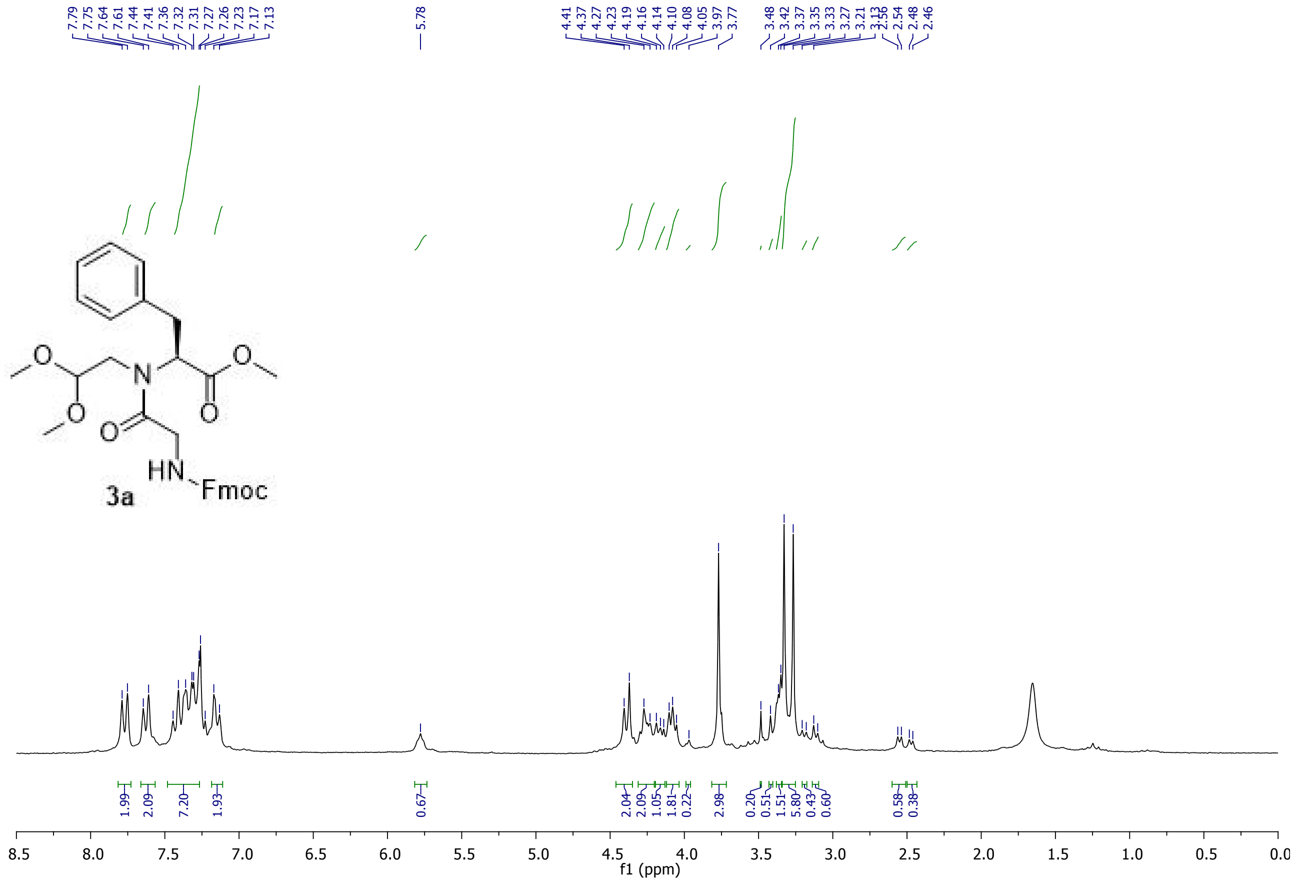

$^{13}C\{^1H\}$ NMR (50 MHz, $CDCl_3$)

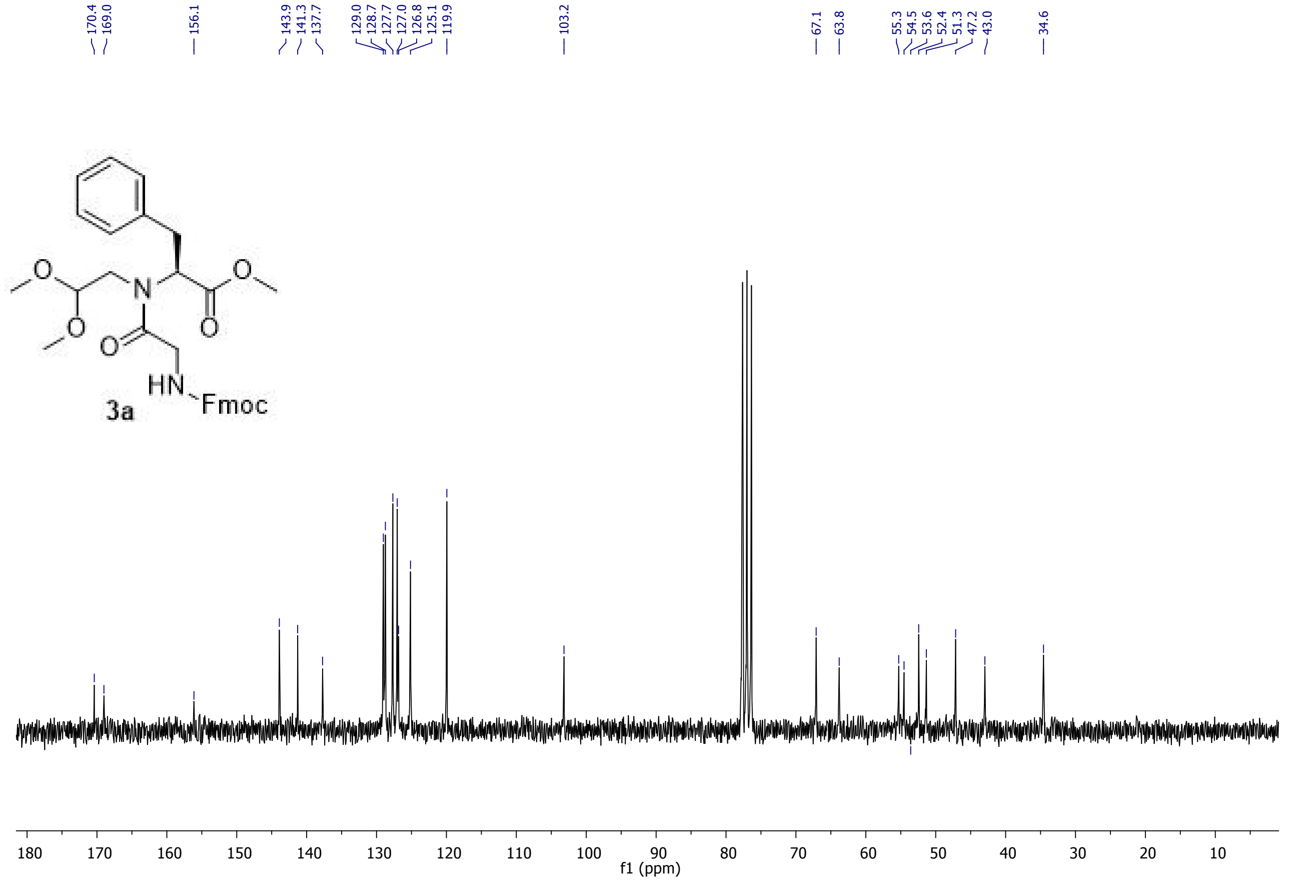

^{1}H NMR (400 MHz, $CDCl_3$)

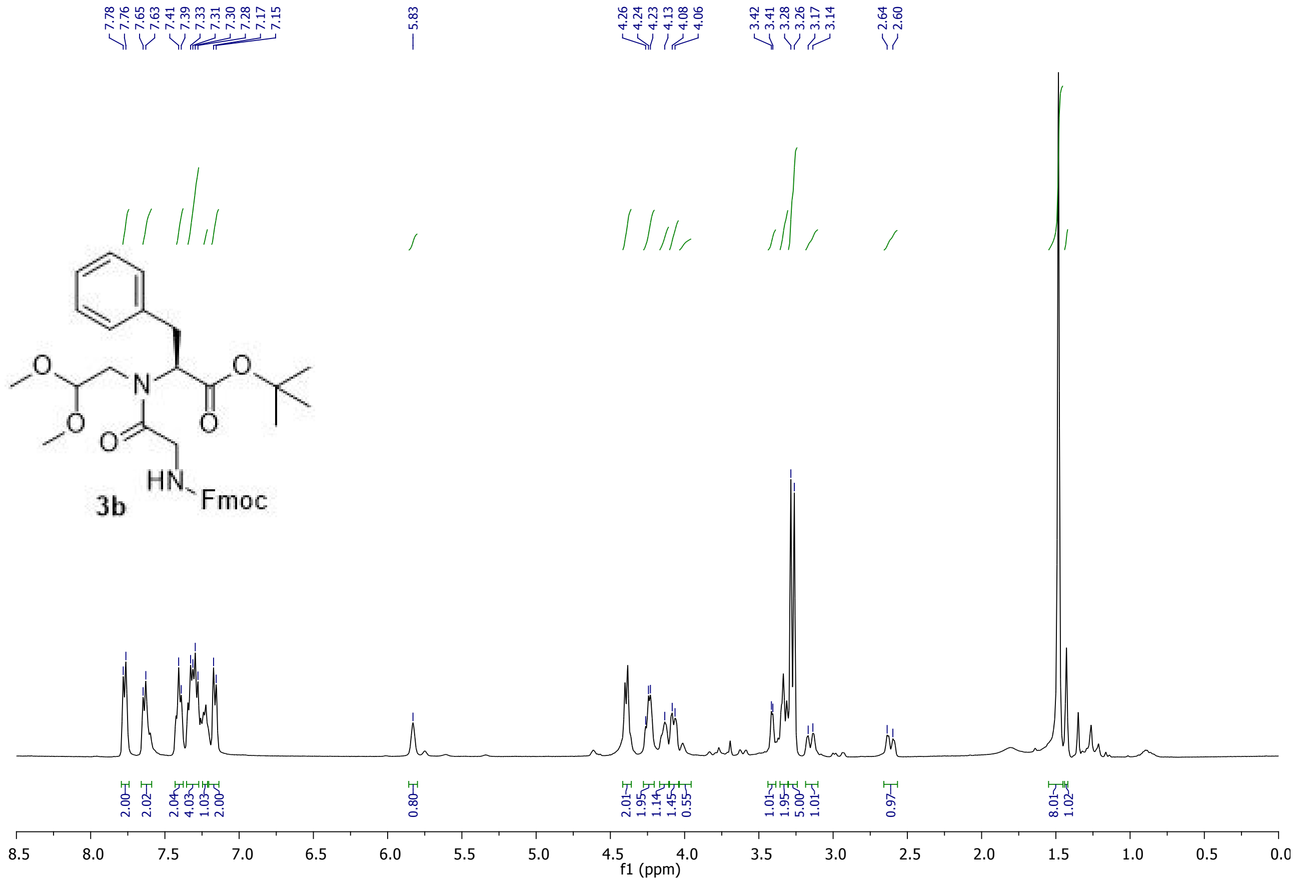

$^{13}C\{^{1}H\}$ NMR (100 MHz, $CDCl_3$)

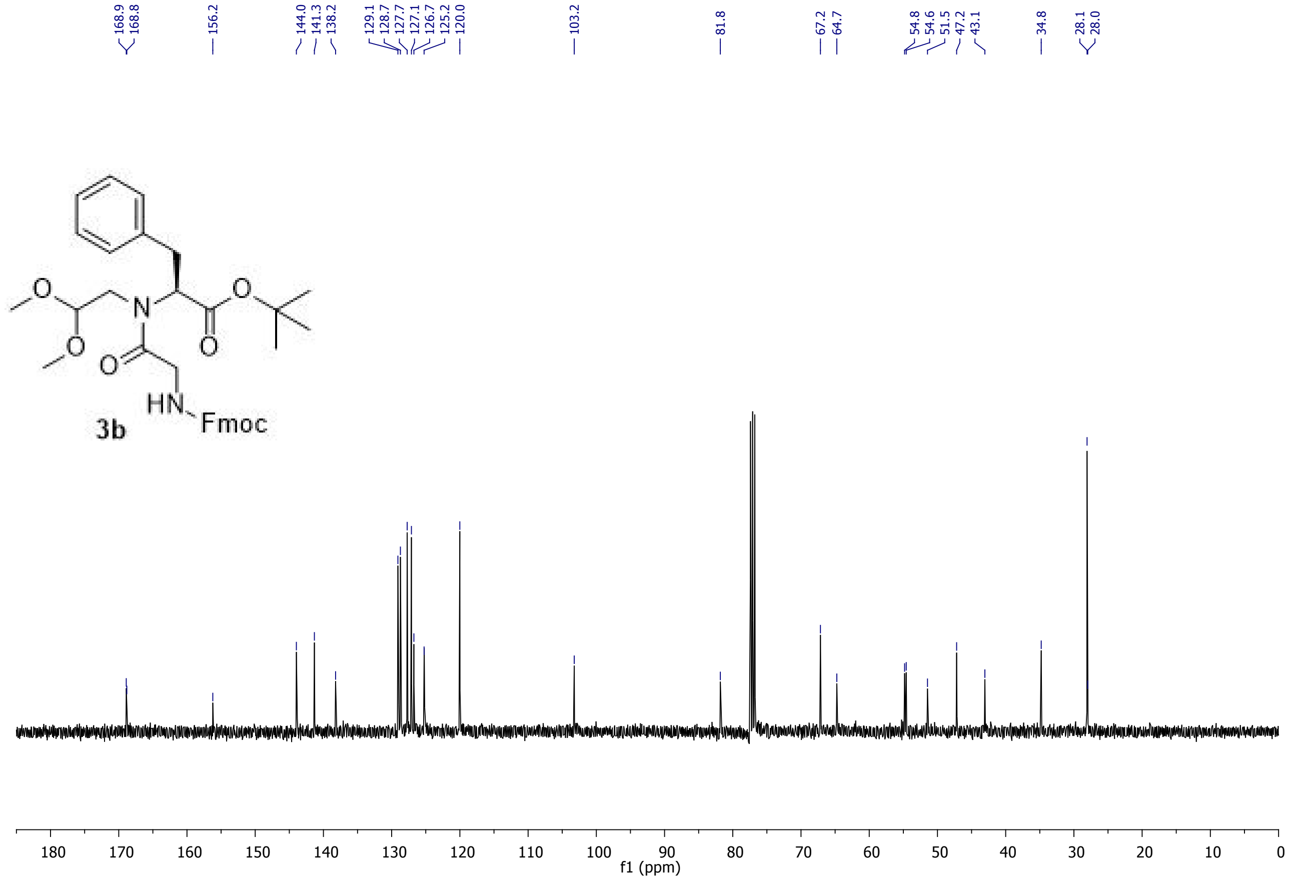

^{1}H NMR (200 MHz, $CDCl_3$)

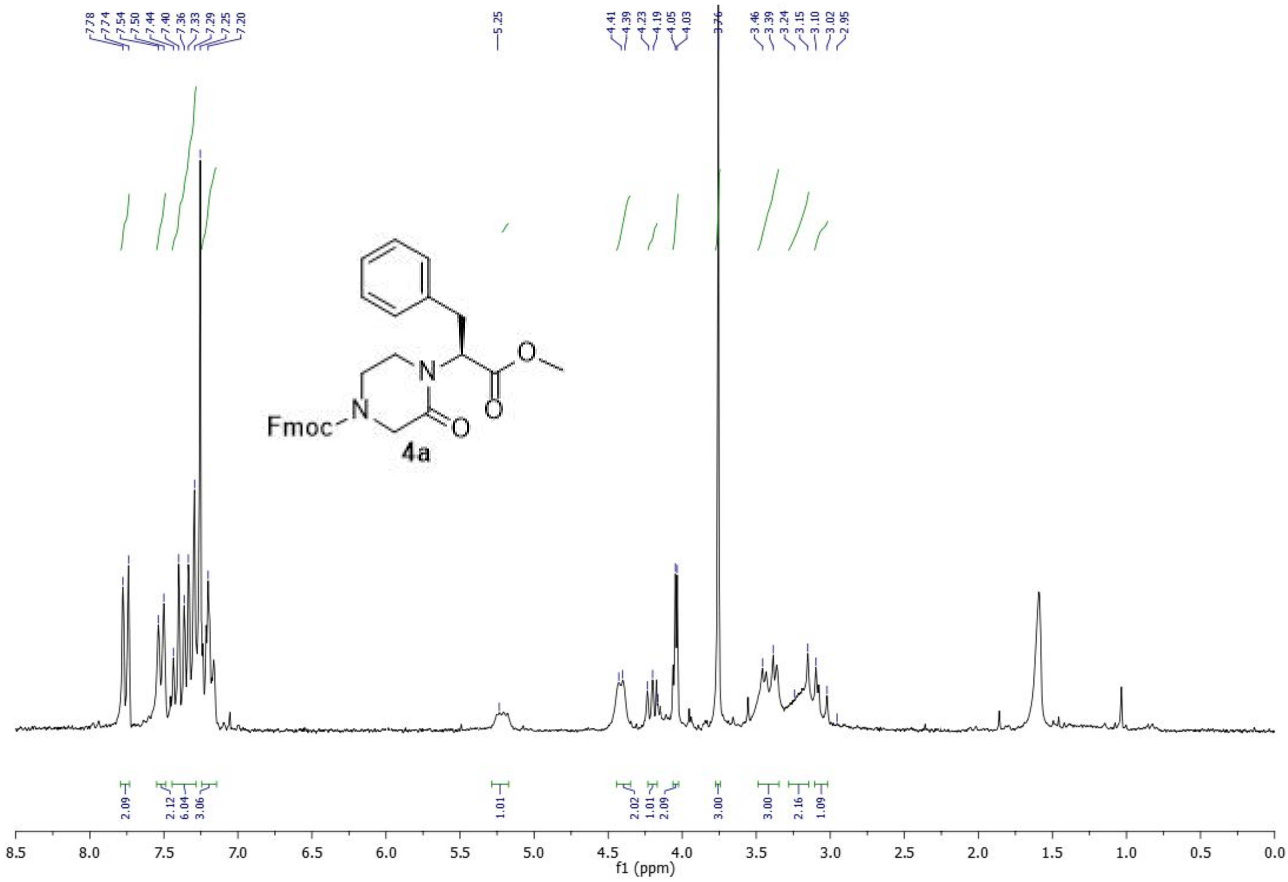

$^{13}C\{^{1}H\}$ NMR (50 MHz, $CDCl_3$)

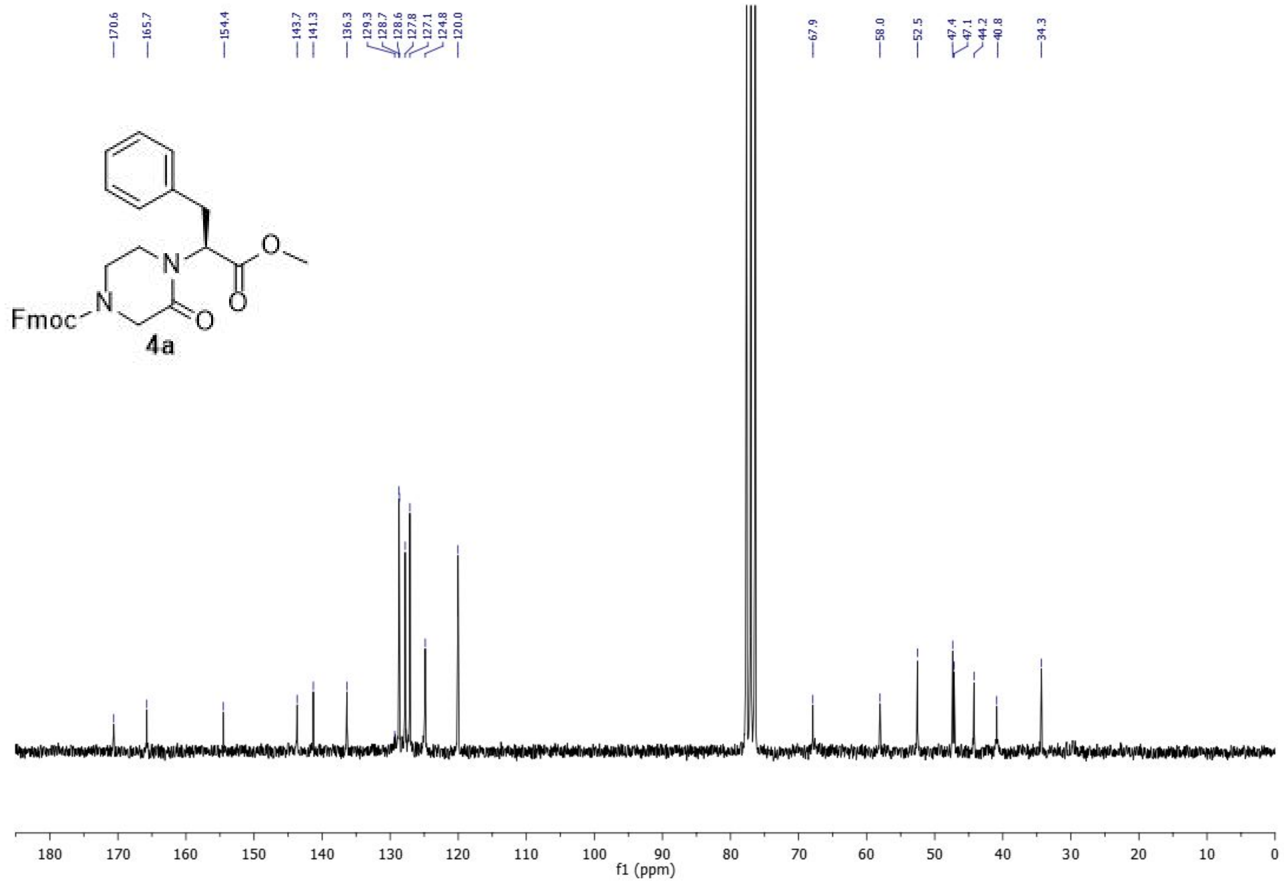

^{1}H NMR (200 MHz, $CDCl_3$)

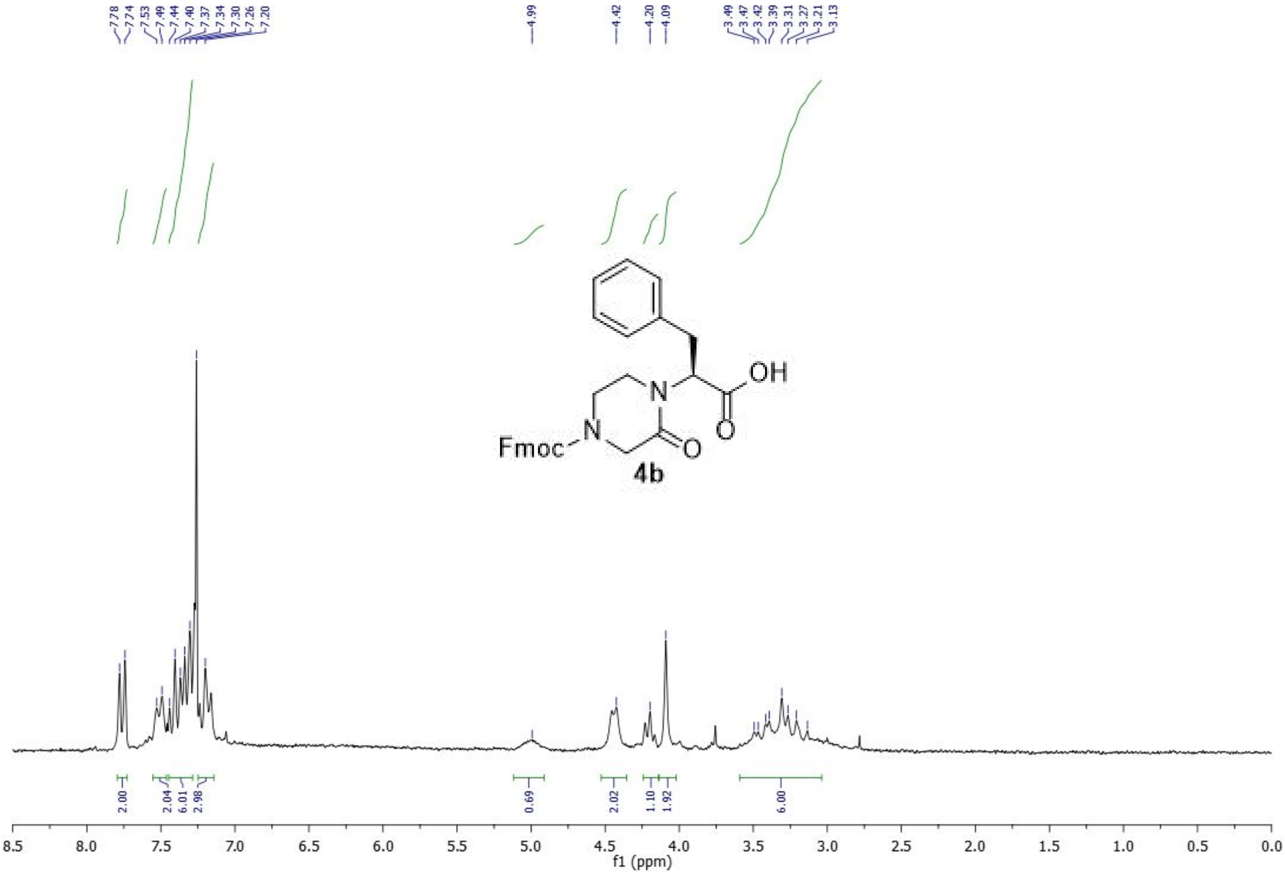

$^{13}C\{^{1}H\}$ NMR (100 MHz, $CDCl_3$)

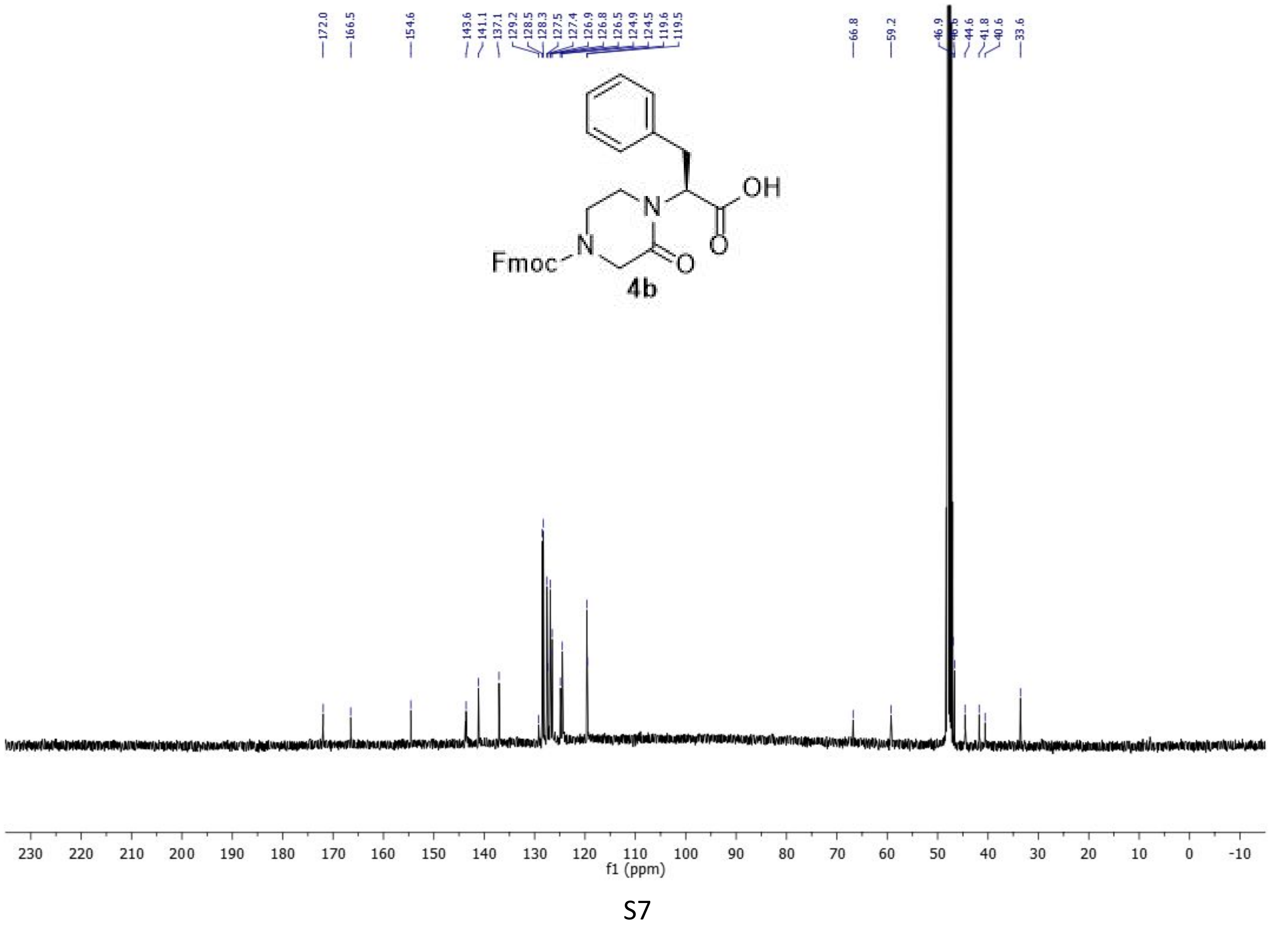

^{1}H NMR (400 MHz, $CDCl_3$)

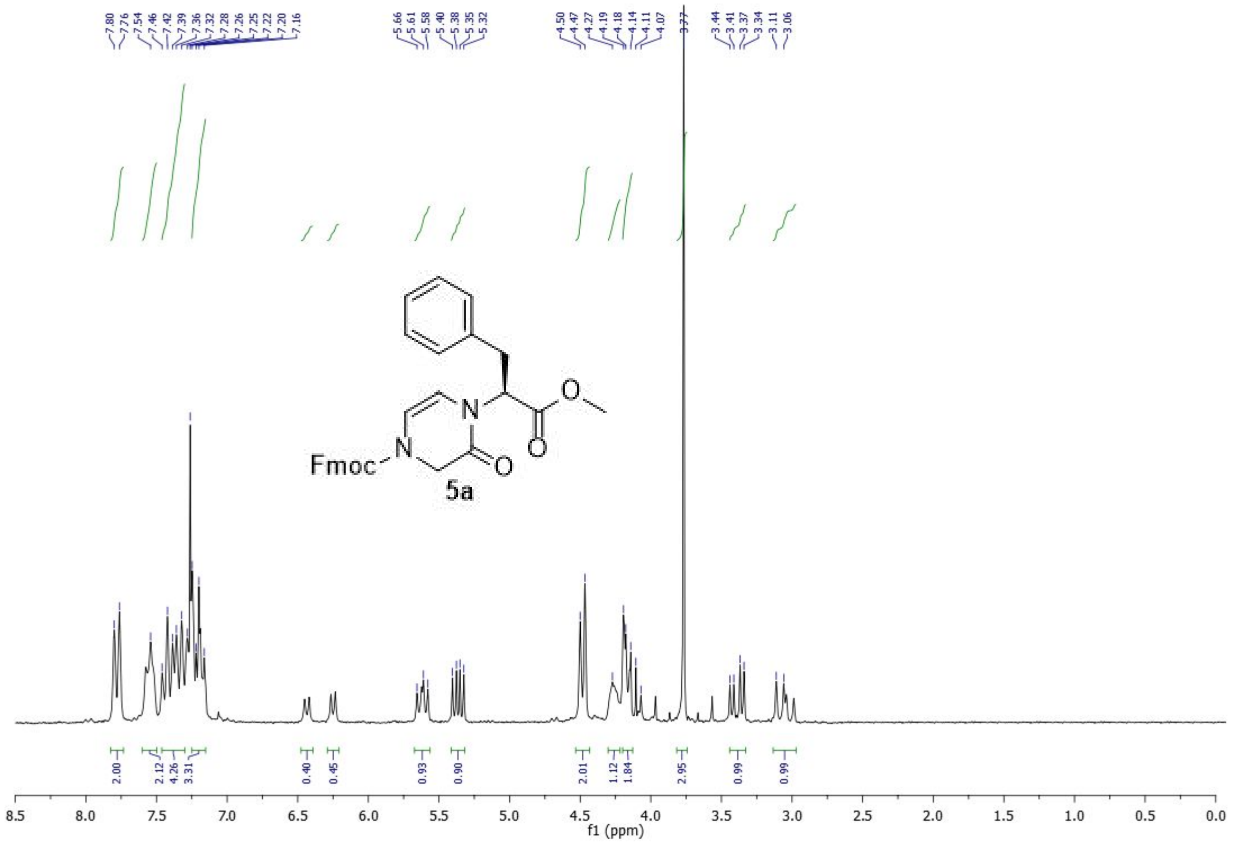

$^{13}C\{^{1}H\}$ NMR (100 MHz, $CDCl_3$)

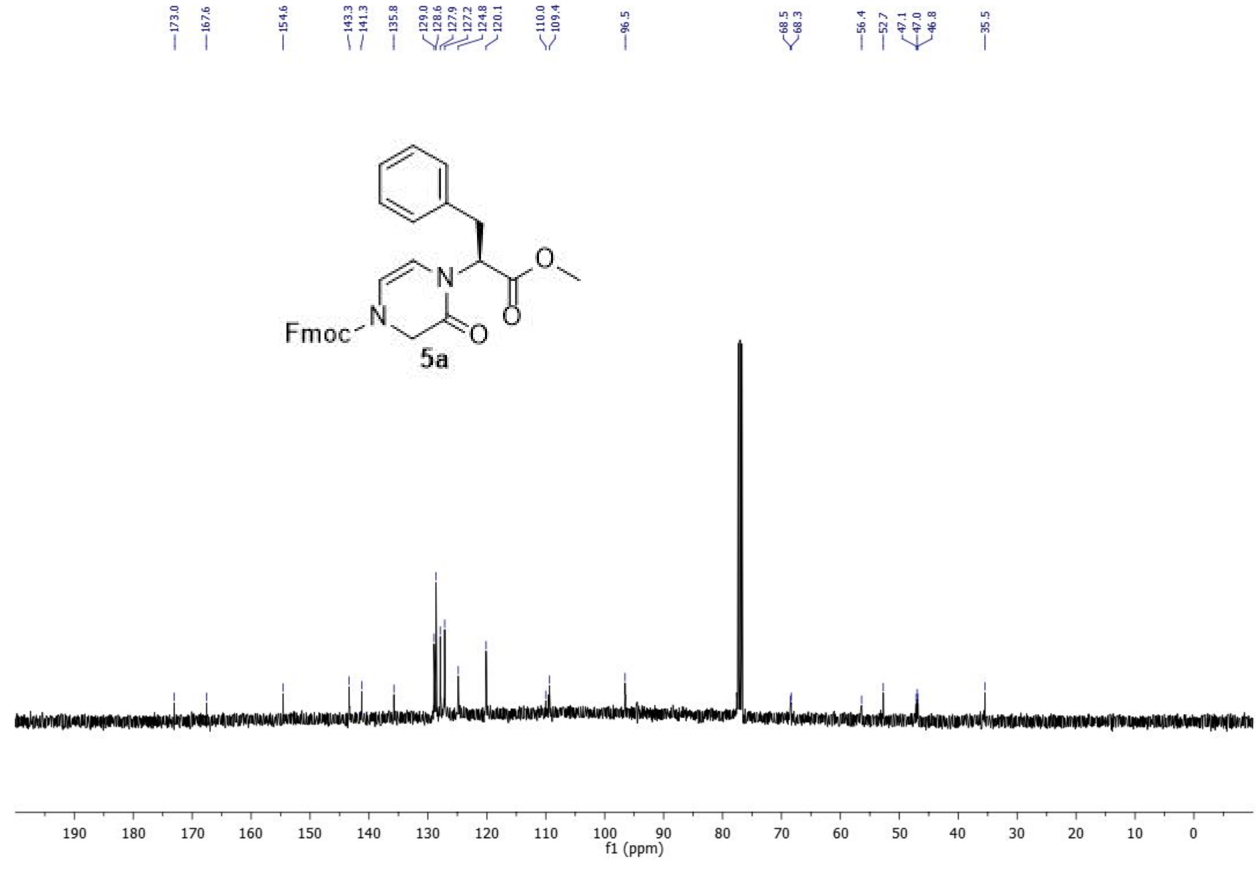

^{1}H NMR (400 MHz, $CDCl_3$)

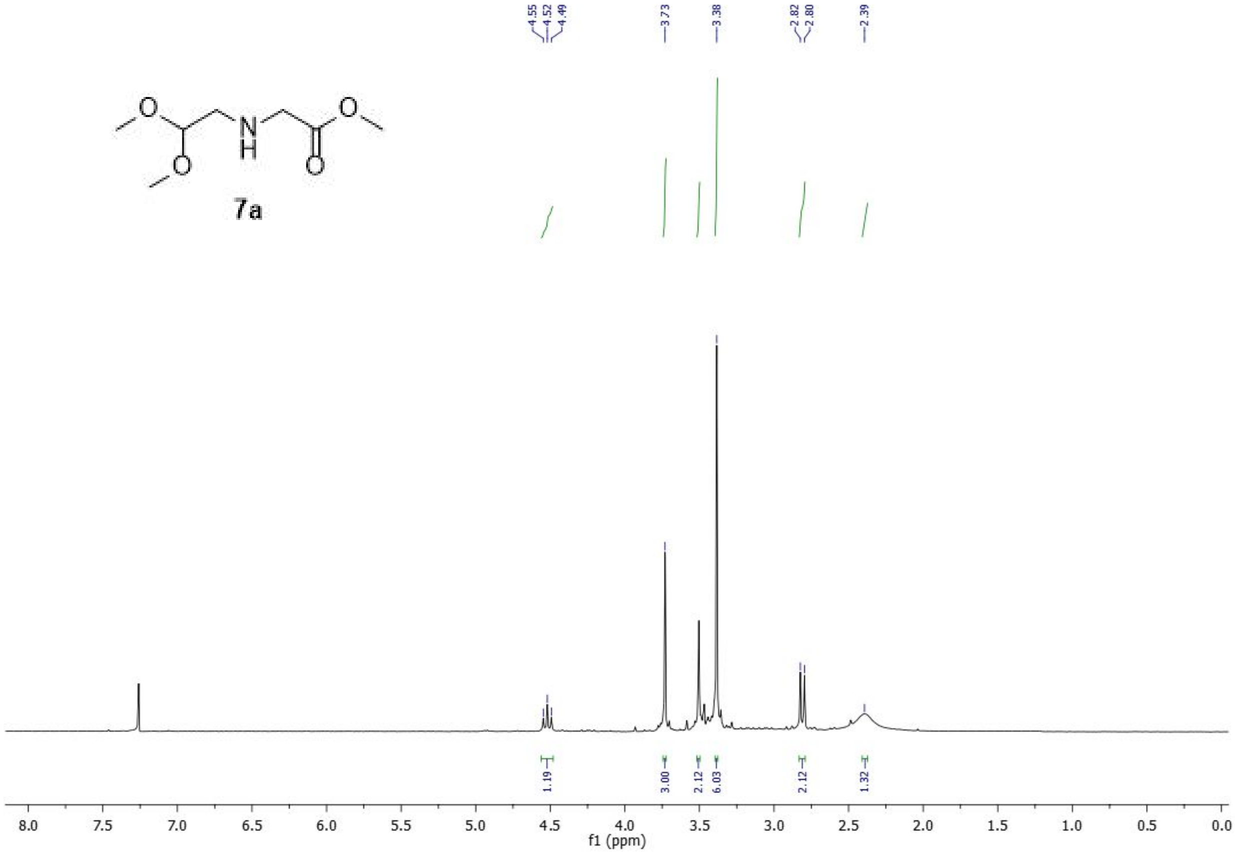

^{1}H NMR (400 MHz, $CDCl_3$)

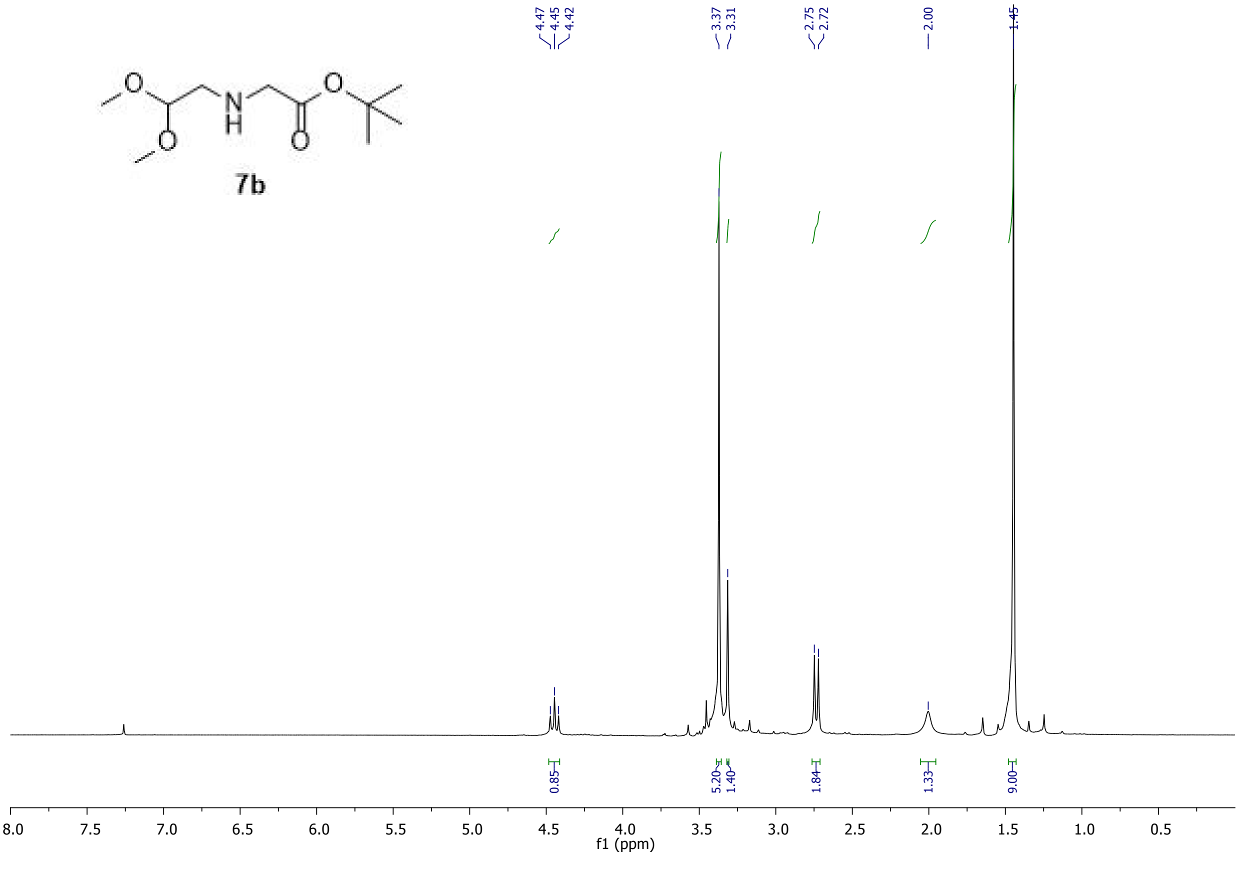

^{1}H NMR (400 MHz, $CDCl_3$)

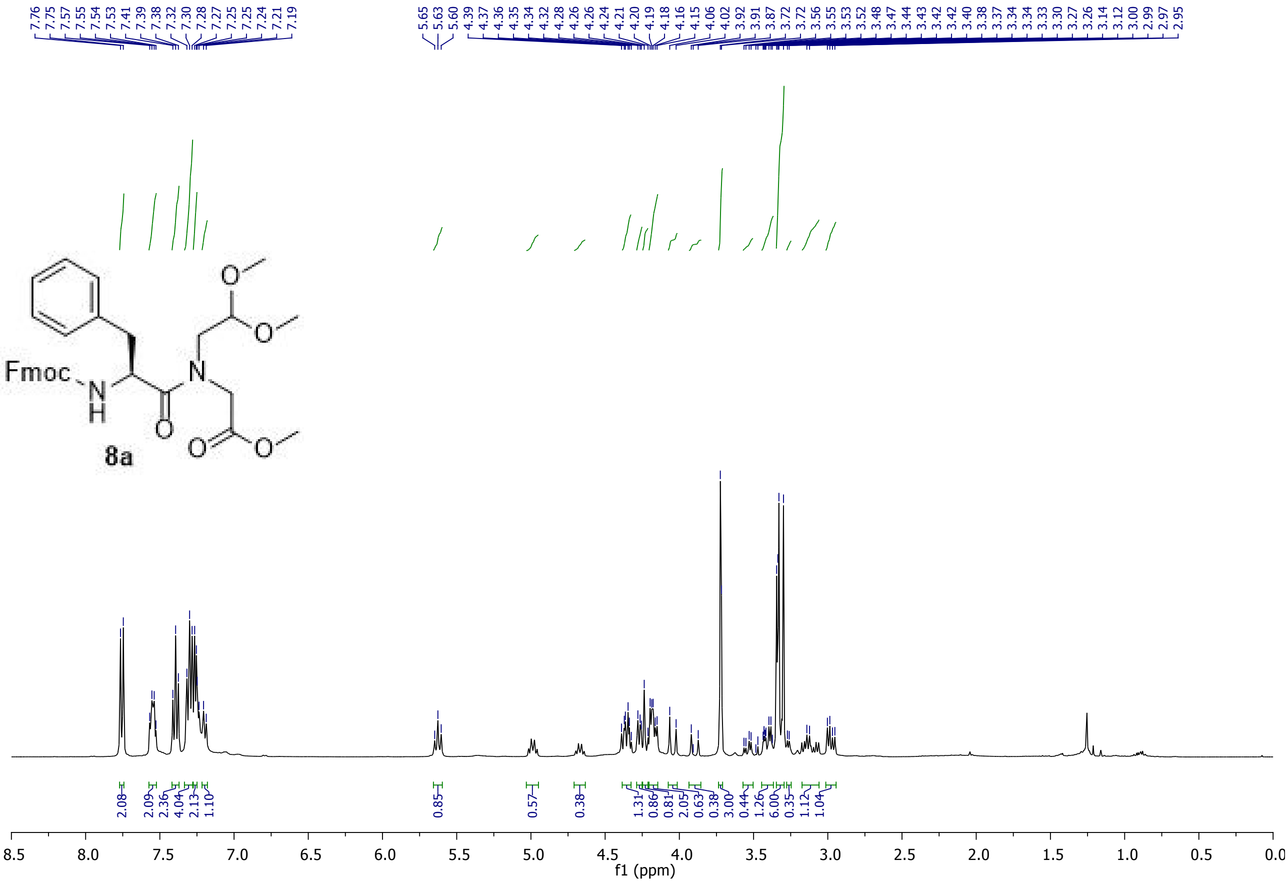

^{13}C{^{1}H} NMR (100 MHz, $CDCl_3$)

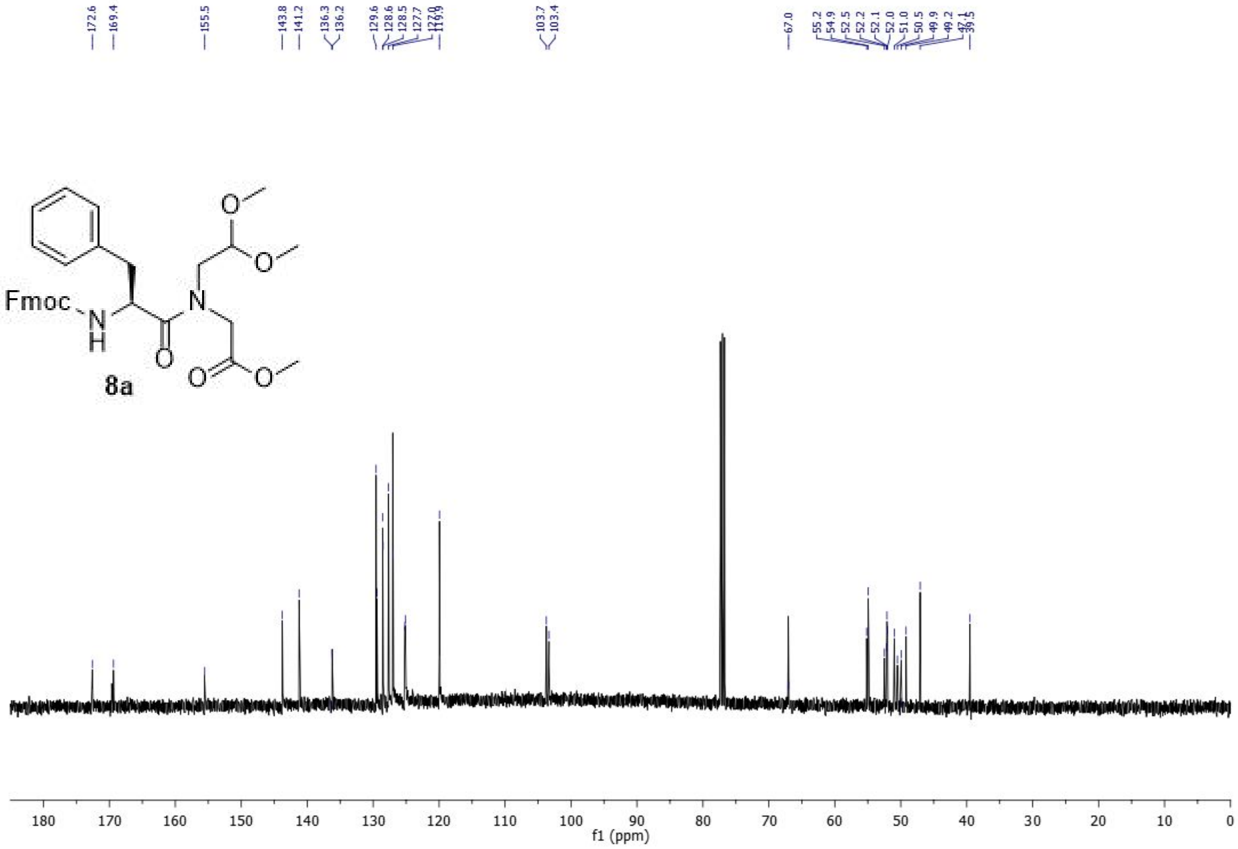

^{1}H NMR (400 MHz, $CDCl_3$)

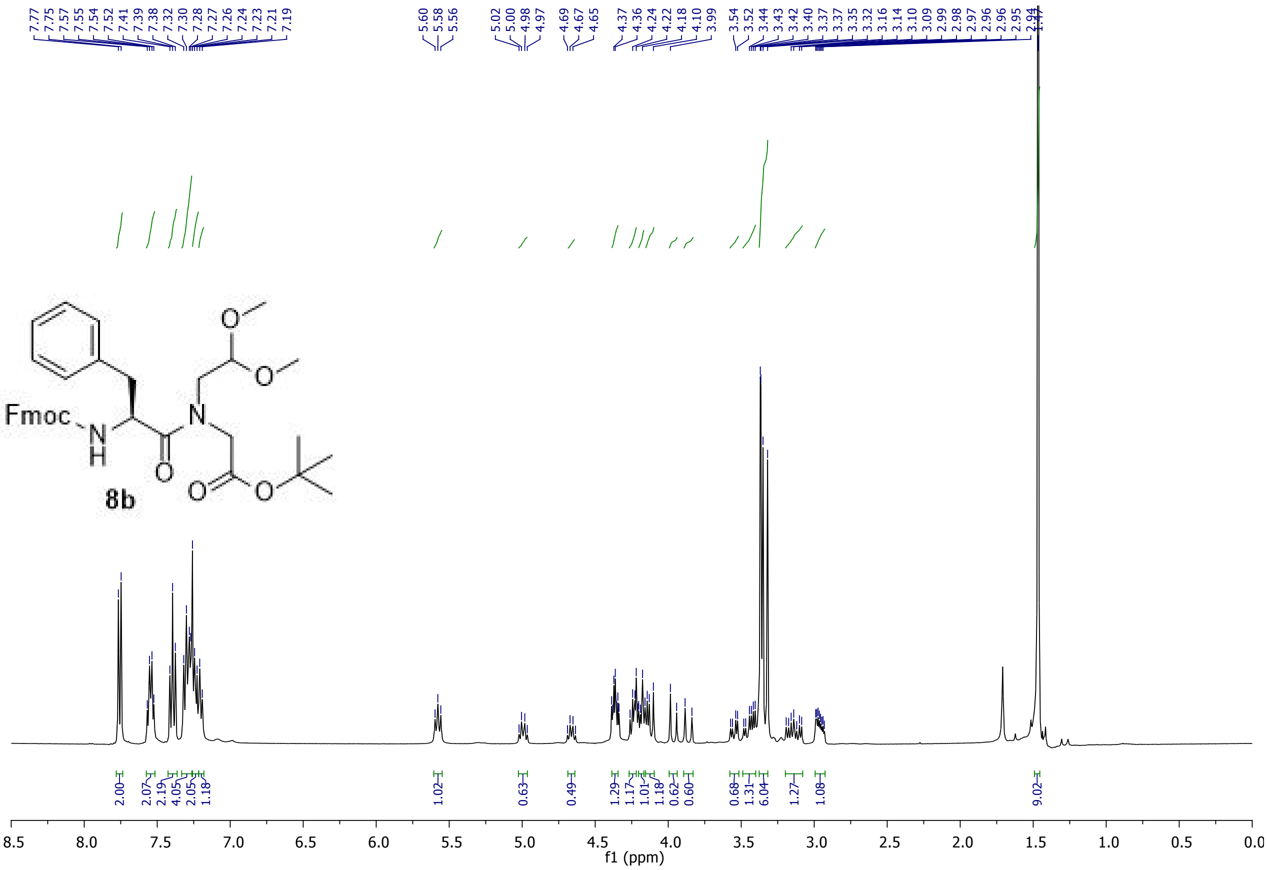

$^{13}C\{^{1}H\}$ NMR (100 MHz, $CDCl_3$)

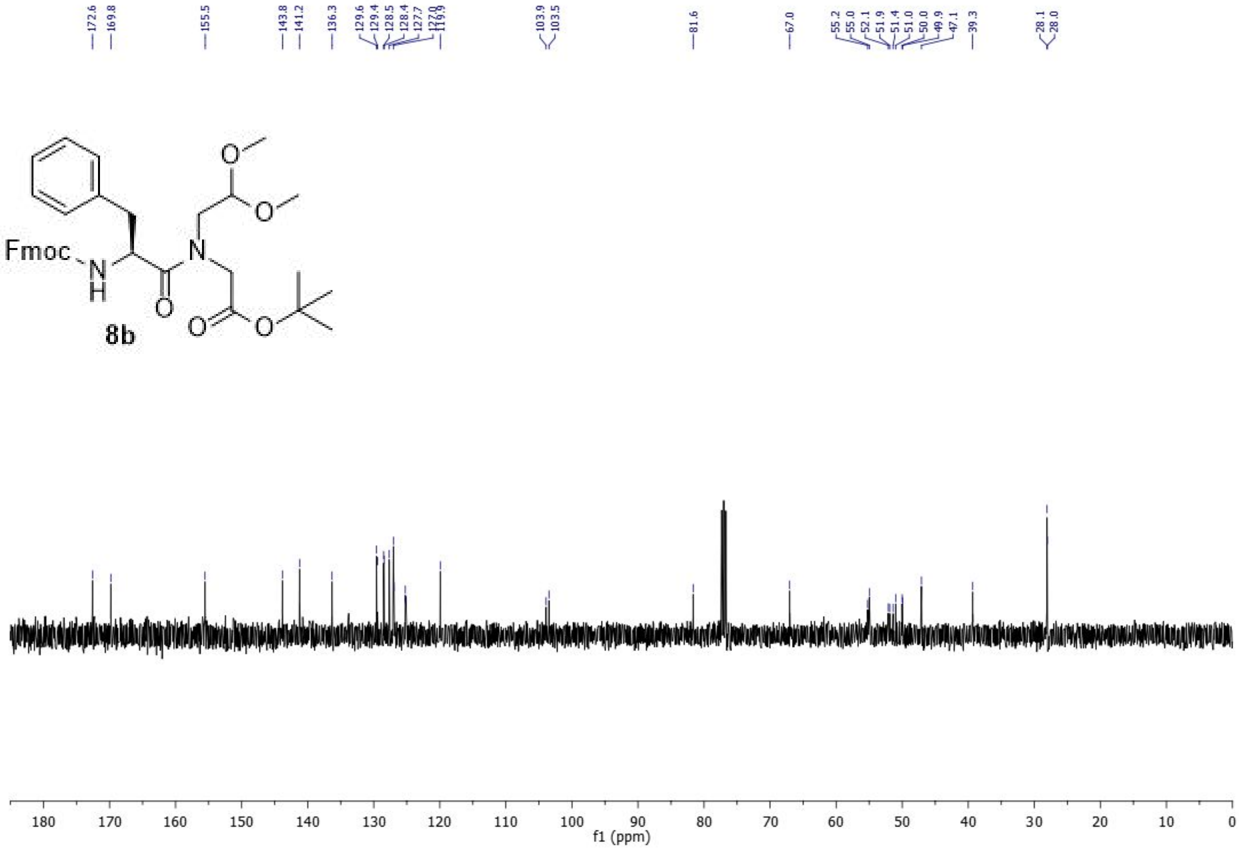

^{1}H NMR (400 MHz, $CDCl_3$)

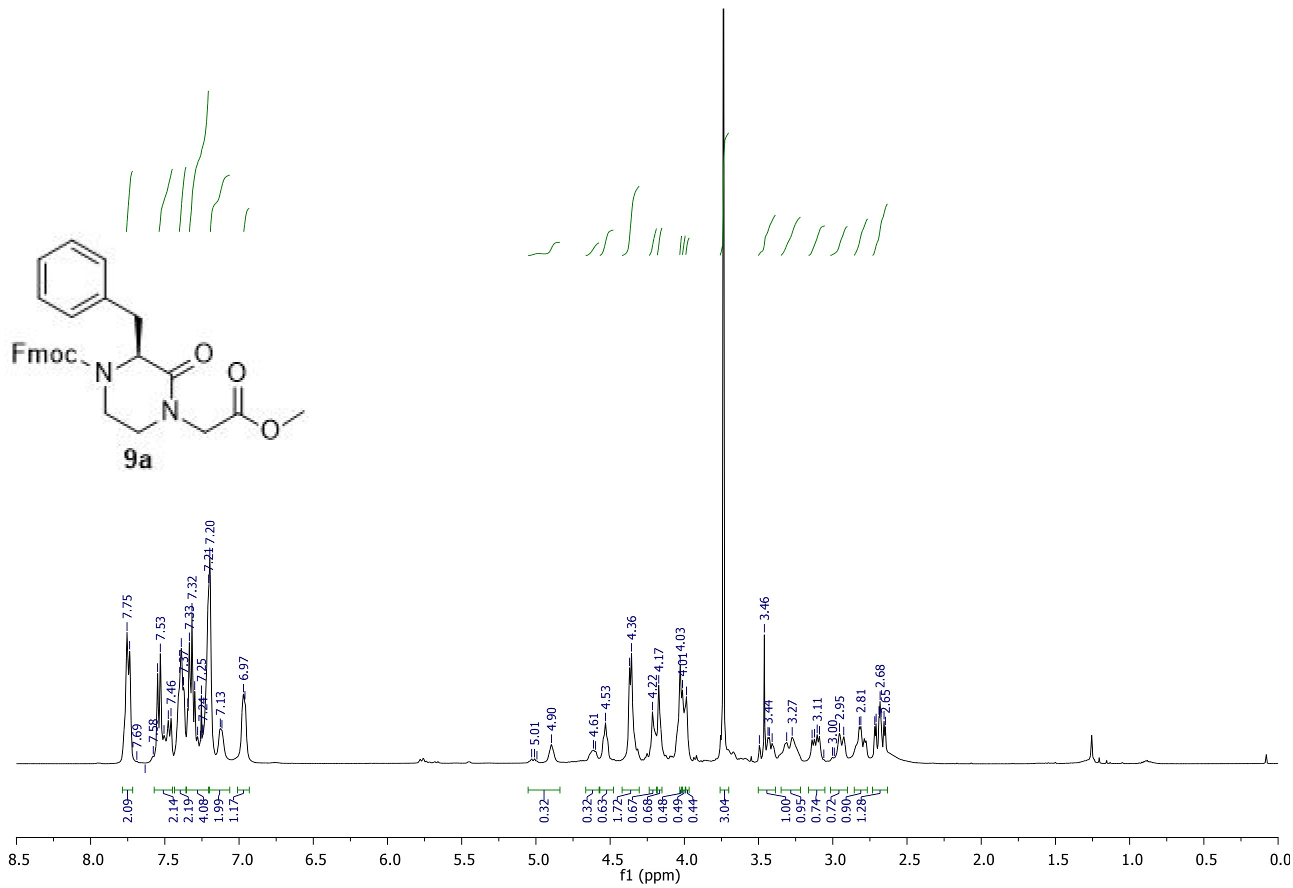

$^{13}C\{^{1}H\}$ NMR (100 MHz, $CDCl_3$)

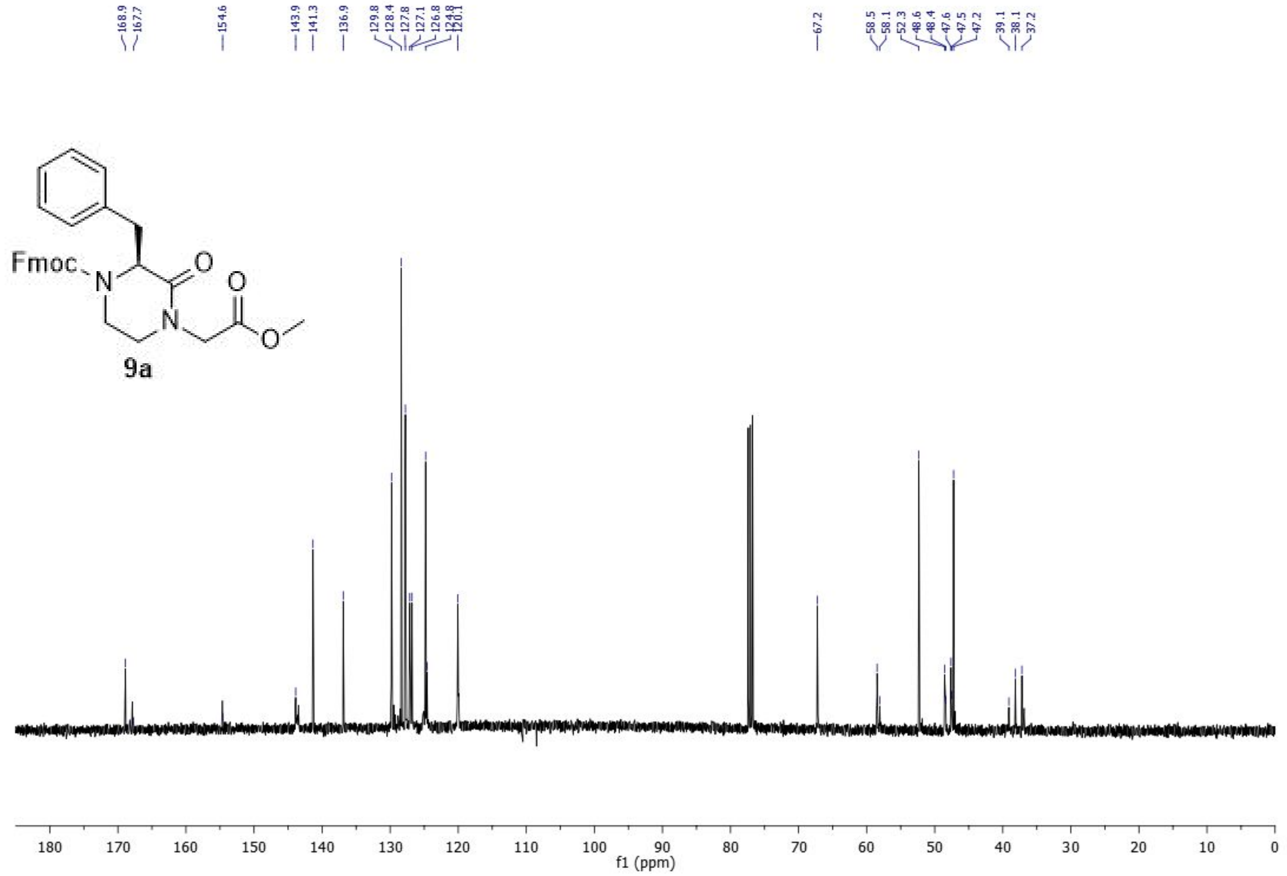

^{1}H NMR (400 MHz, $CDCl_3$)

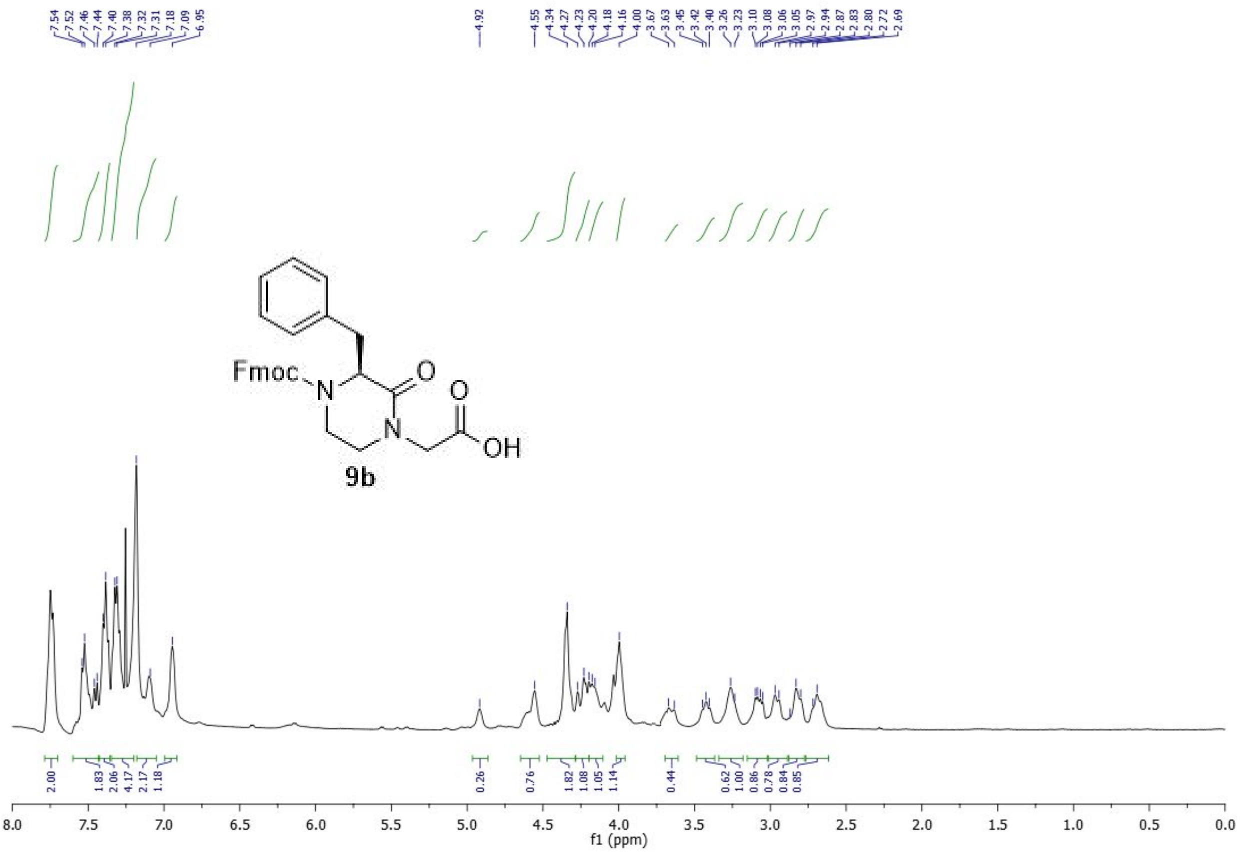

$^{13}C\{^{1}H\}$ NMR (100 MHz, $CDCl_3$)

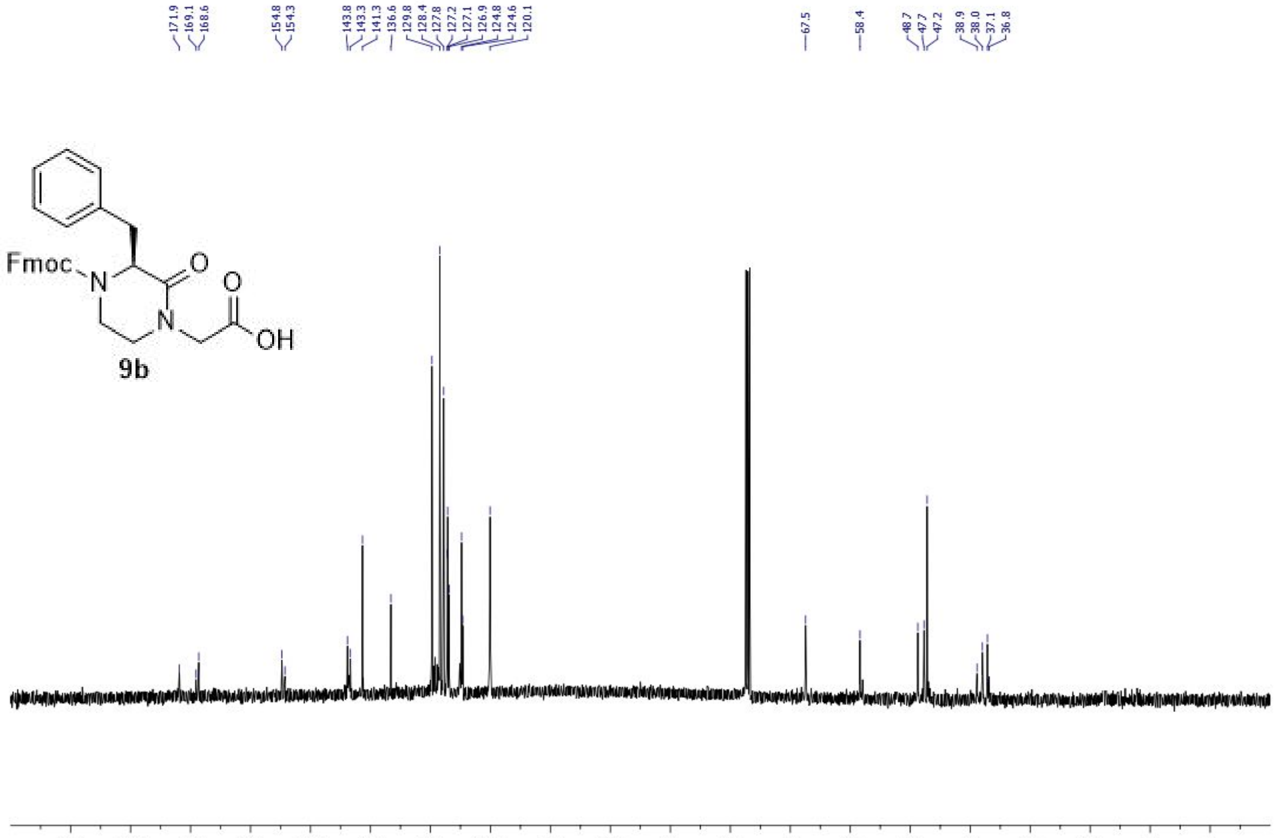

^{1}H NMR (400 MHz, $CDCl_3$)

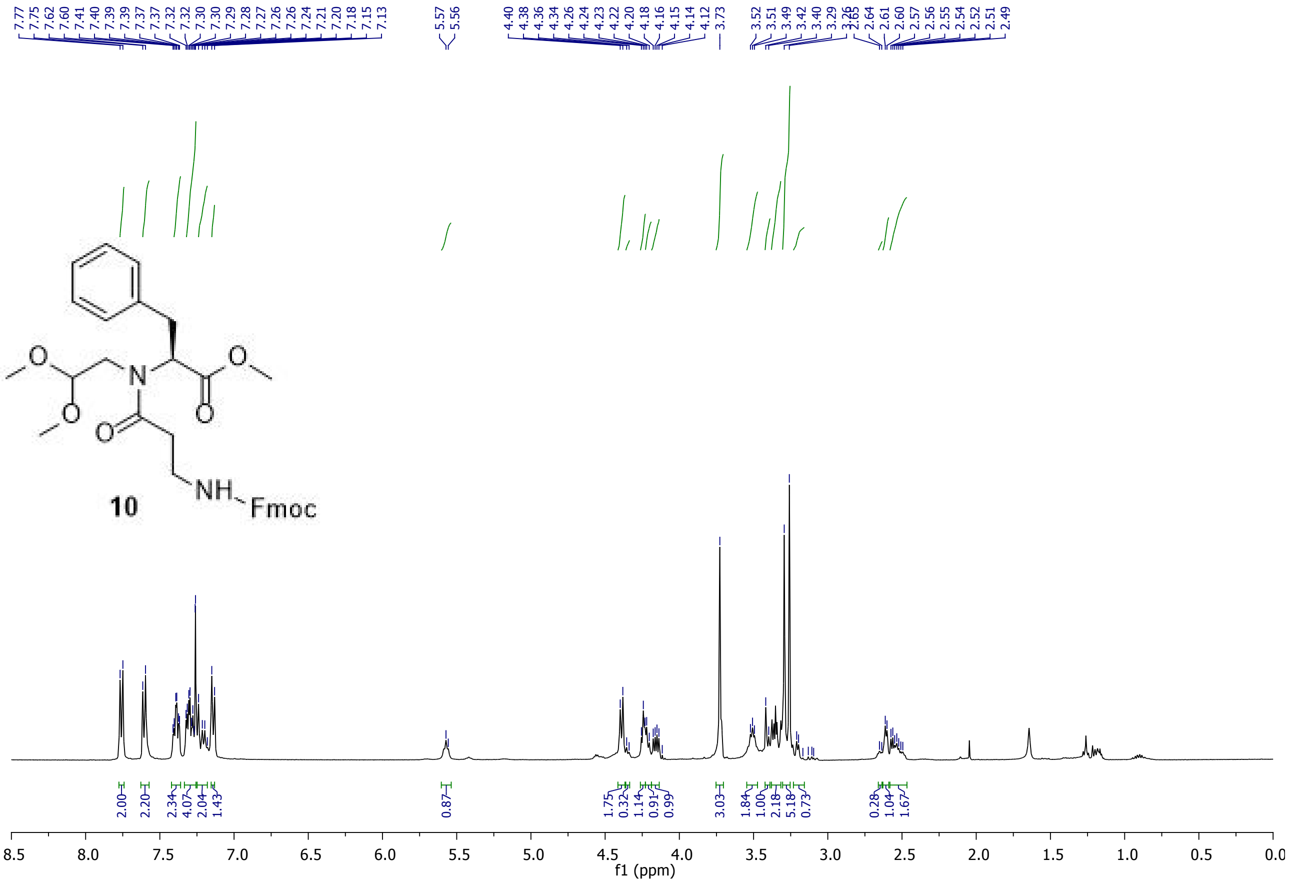

$^{13}C\{^{1}H\}$ NMR (100 MHz, $CDCl_3$)

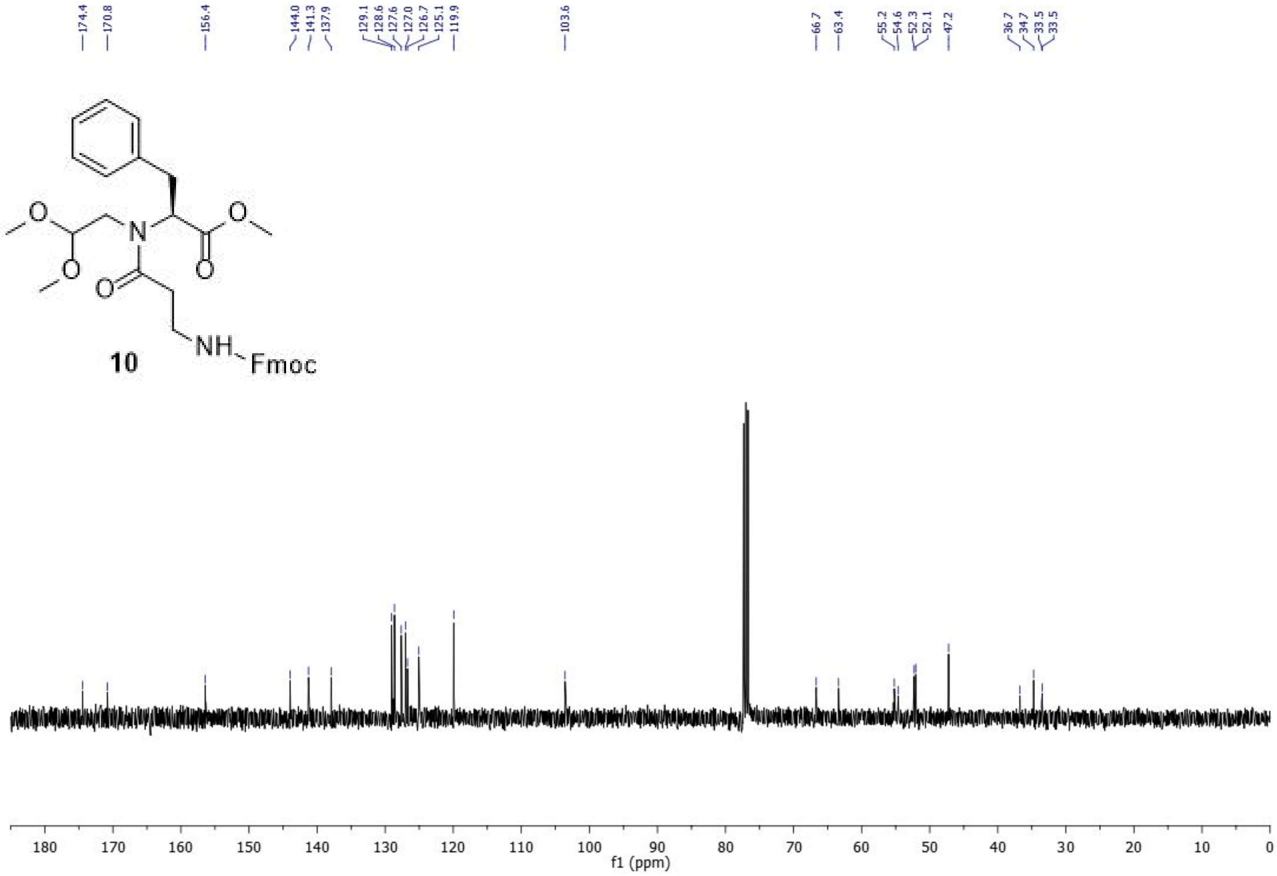

^{1}H NMR (400 MHz, $CDCl_3$)

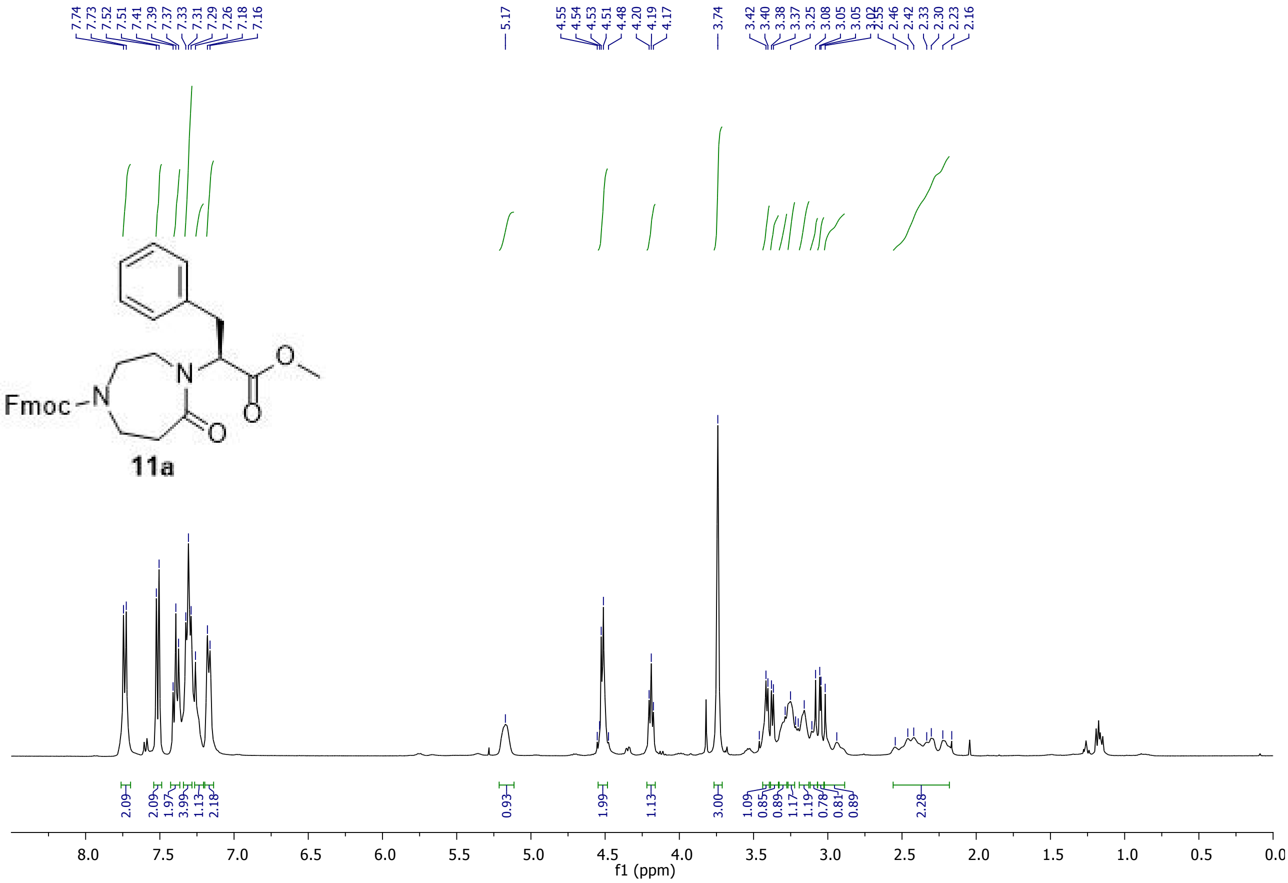

^{13}C{^{1}H} NMR (50 MHz, $CDCl_3$)

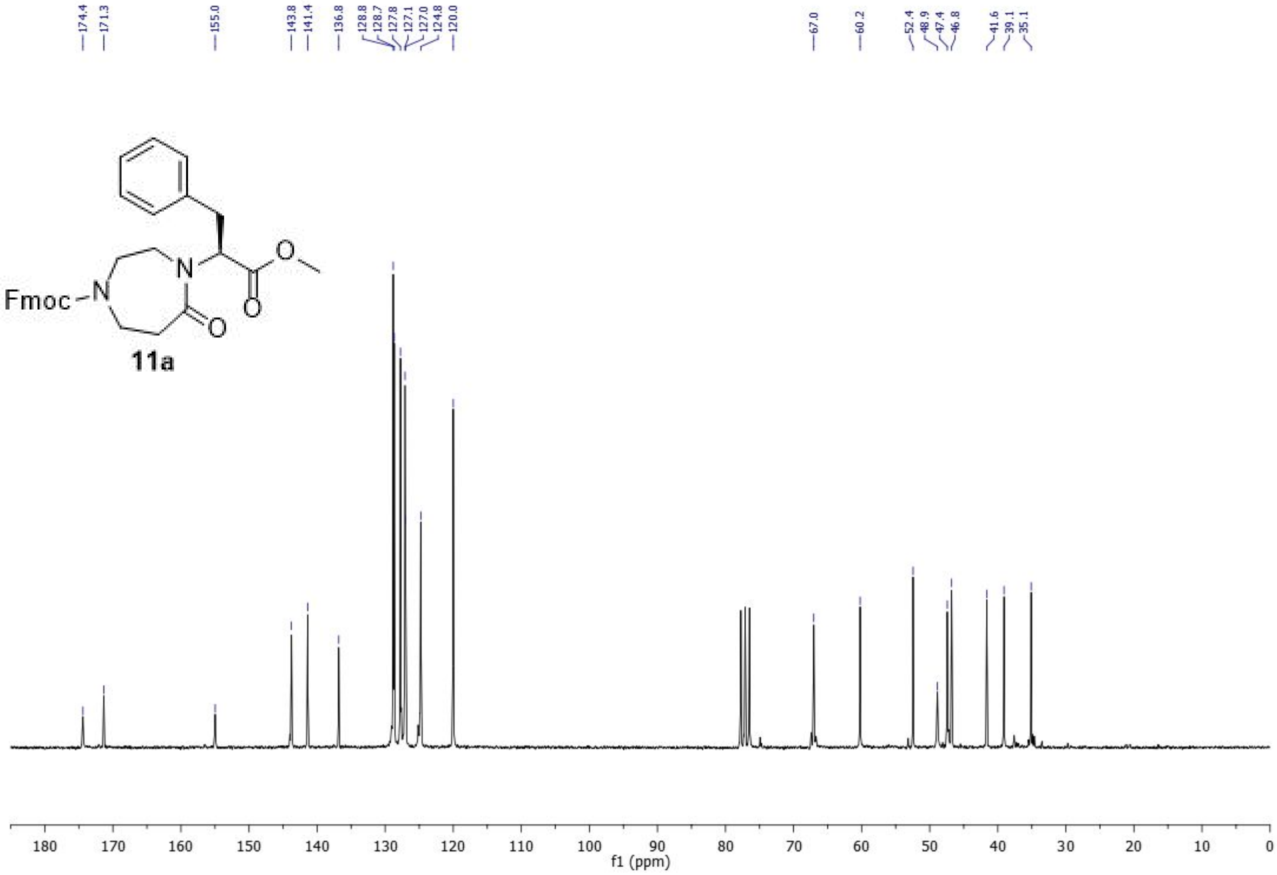

^{1}H NMR (400 MHz, $CDCl_3$)

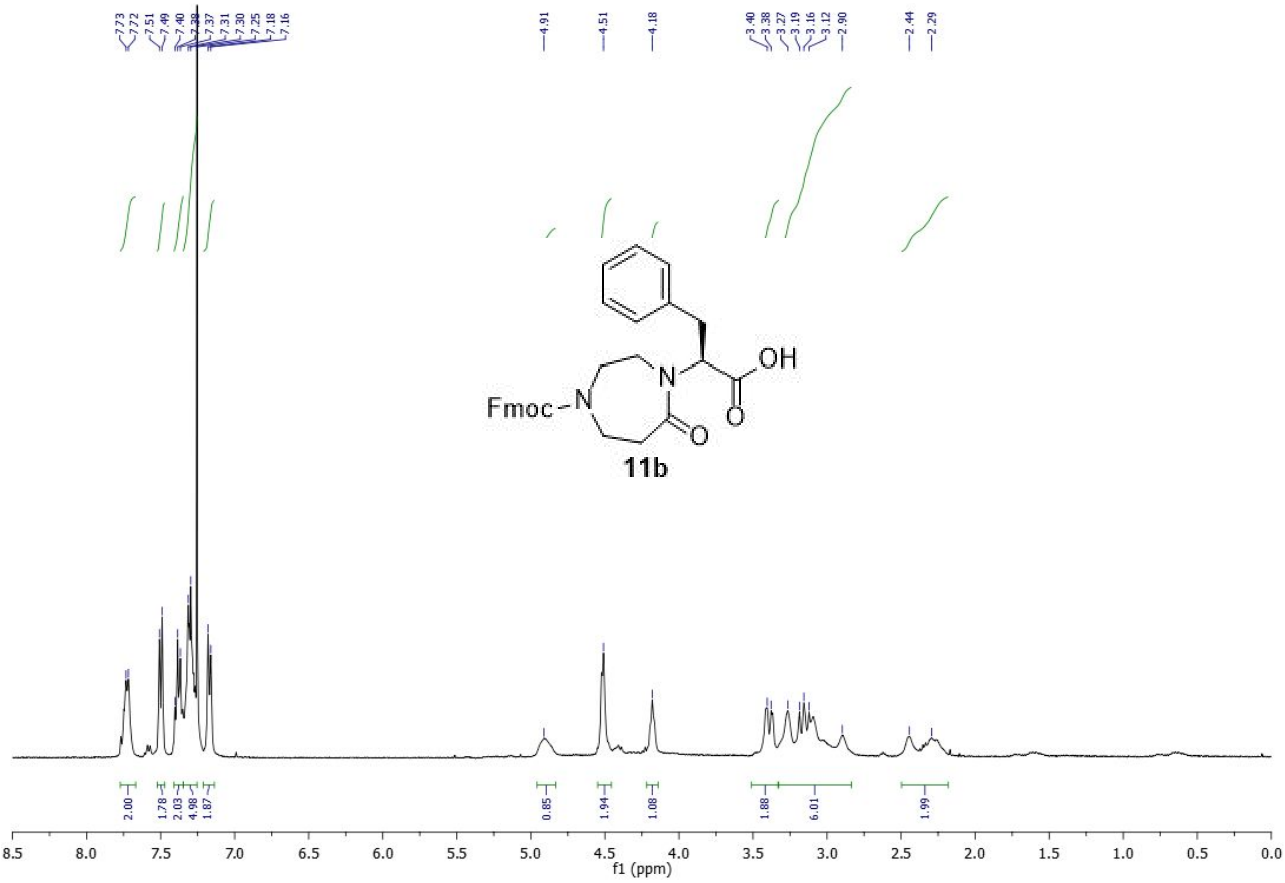

$^{13}C\{^{1}H\}$ NMR (50 MHz, $CDCl_3$)

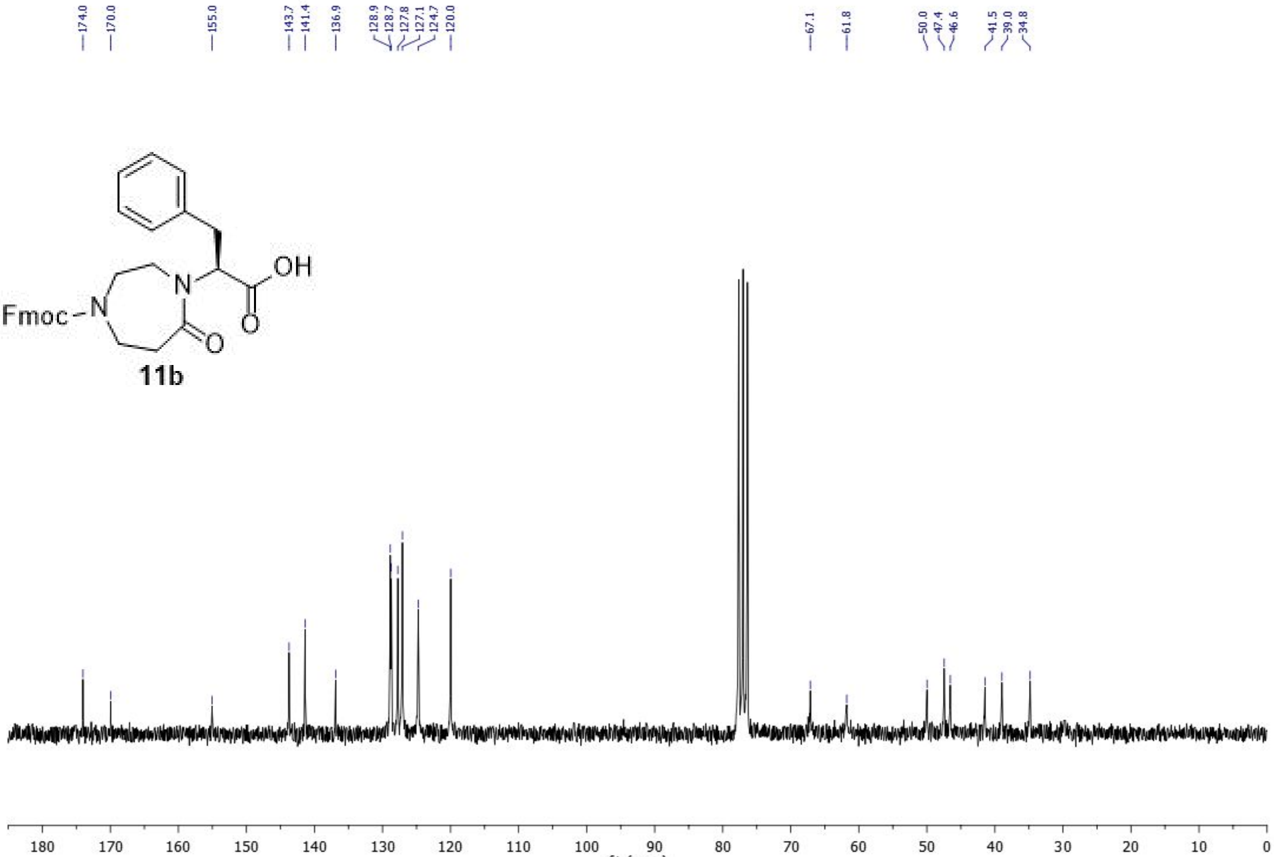

^{1}H NMR (400 MHz, $CDCl_3$)

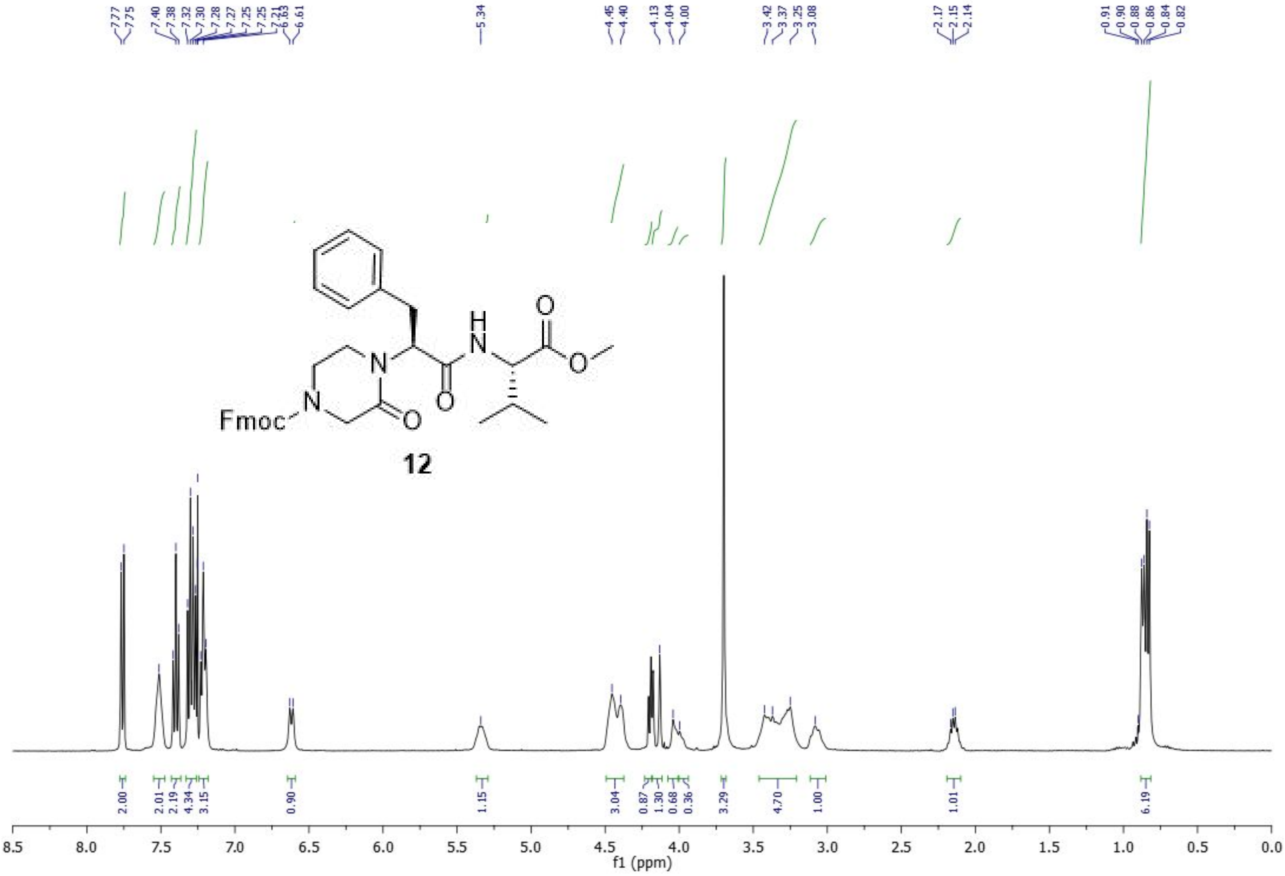

$^{13}C\{^{1}H\}$ NMR (100 MHz, $CDCl_3$)

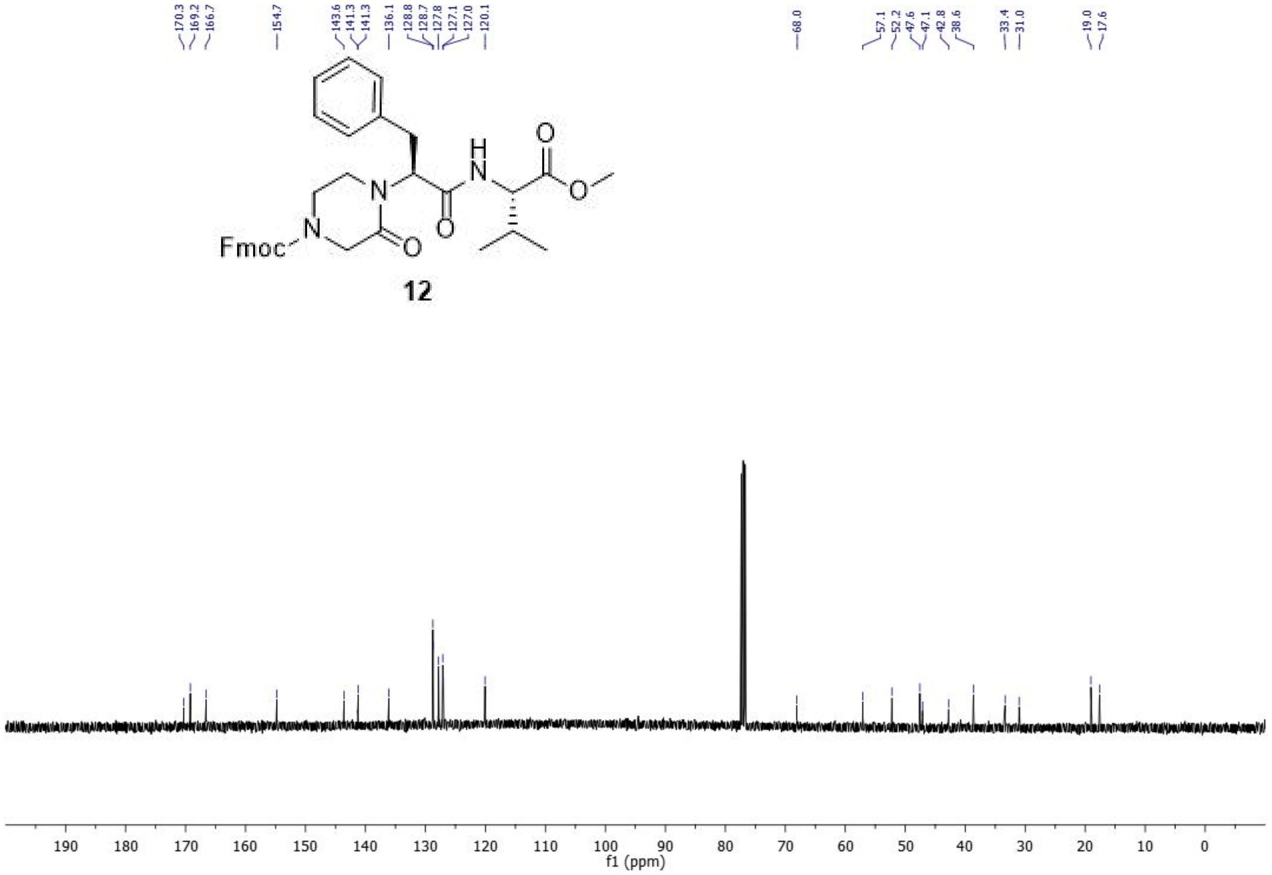

^{1}H NMR (400 MHz, $CDCl_3$)

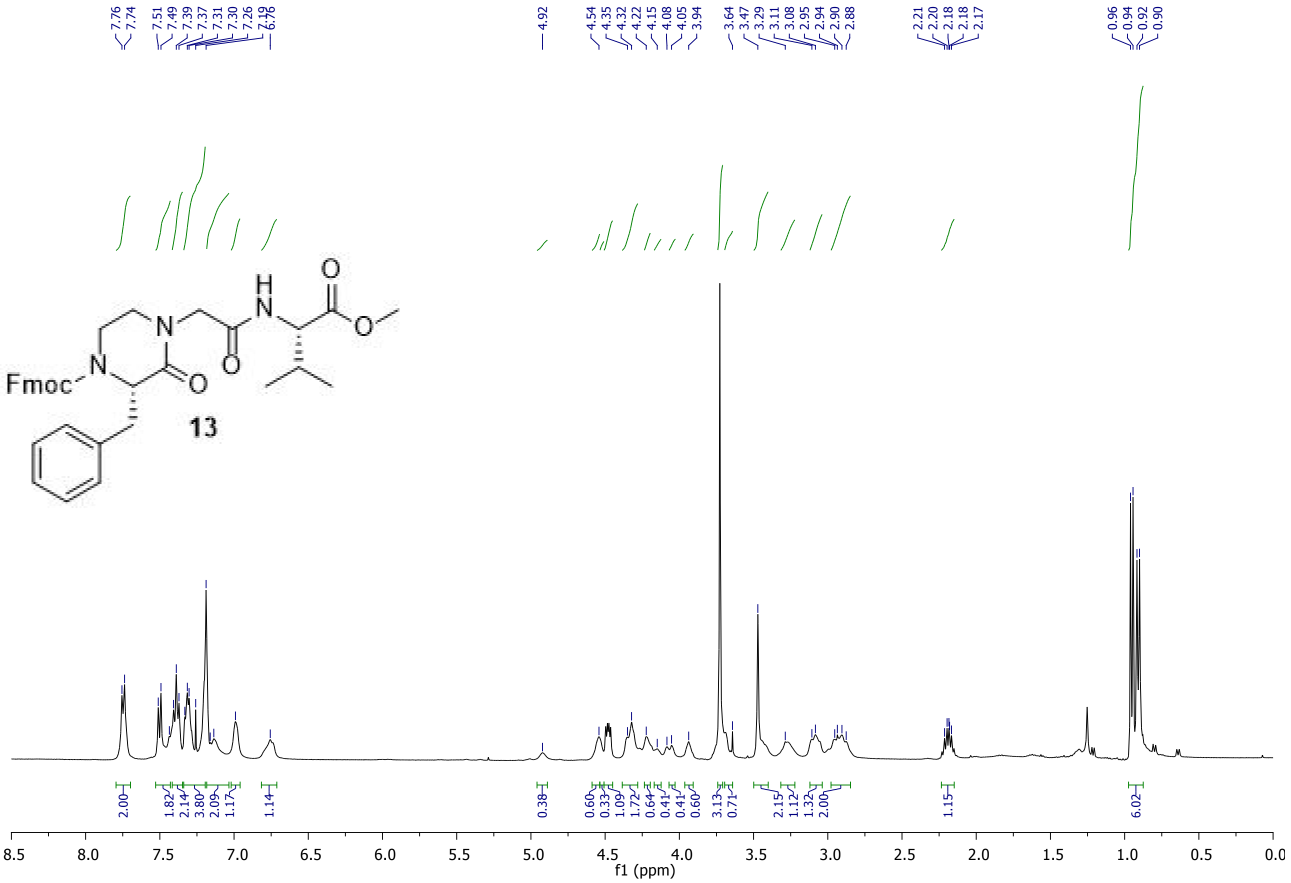

^{13}C{^{1}H} NMR (100 MHz, $CDCl_3$)

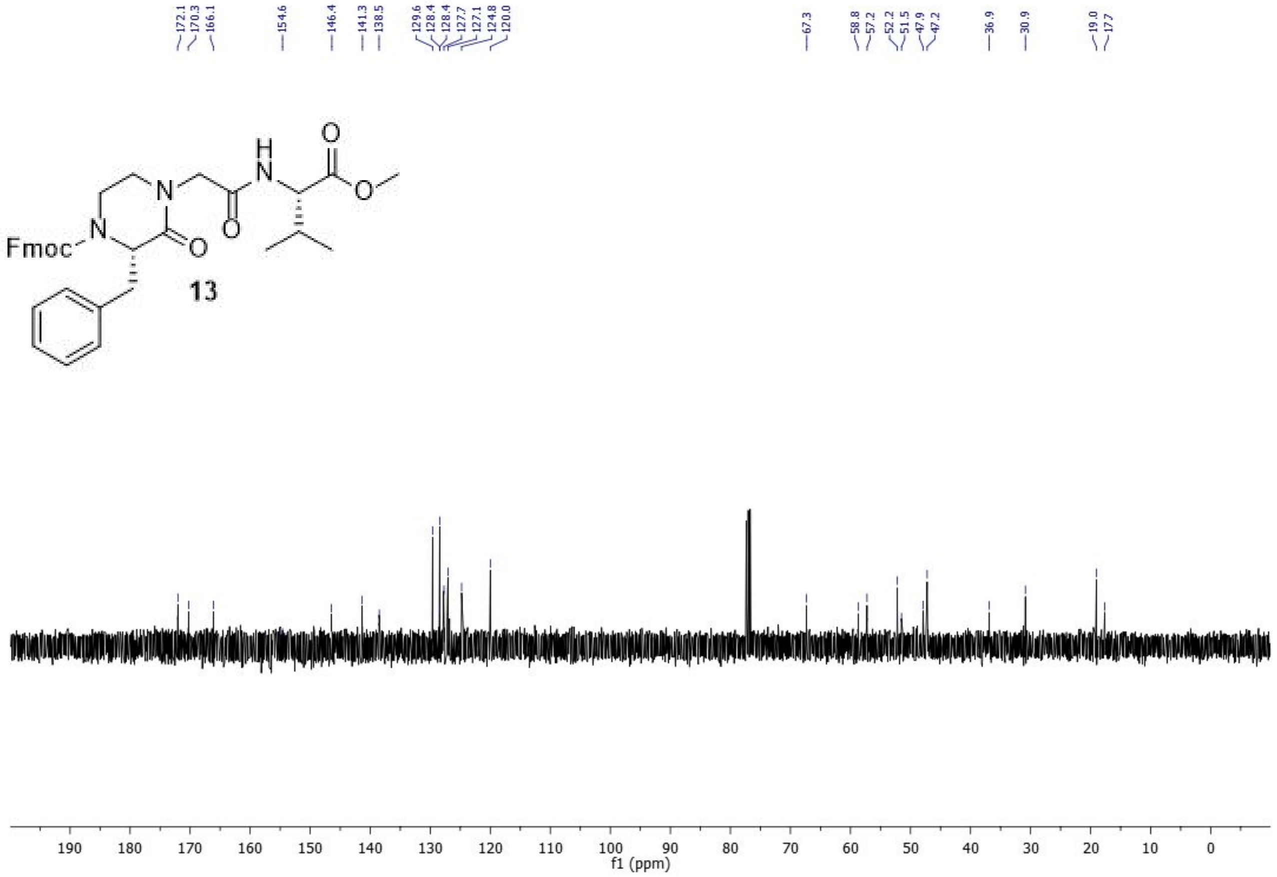

^{1}H NMR (400 MHz, $CDCl_3$)

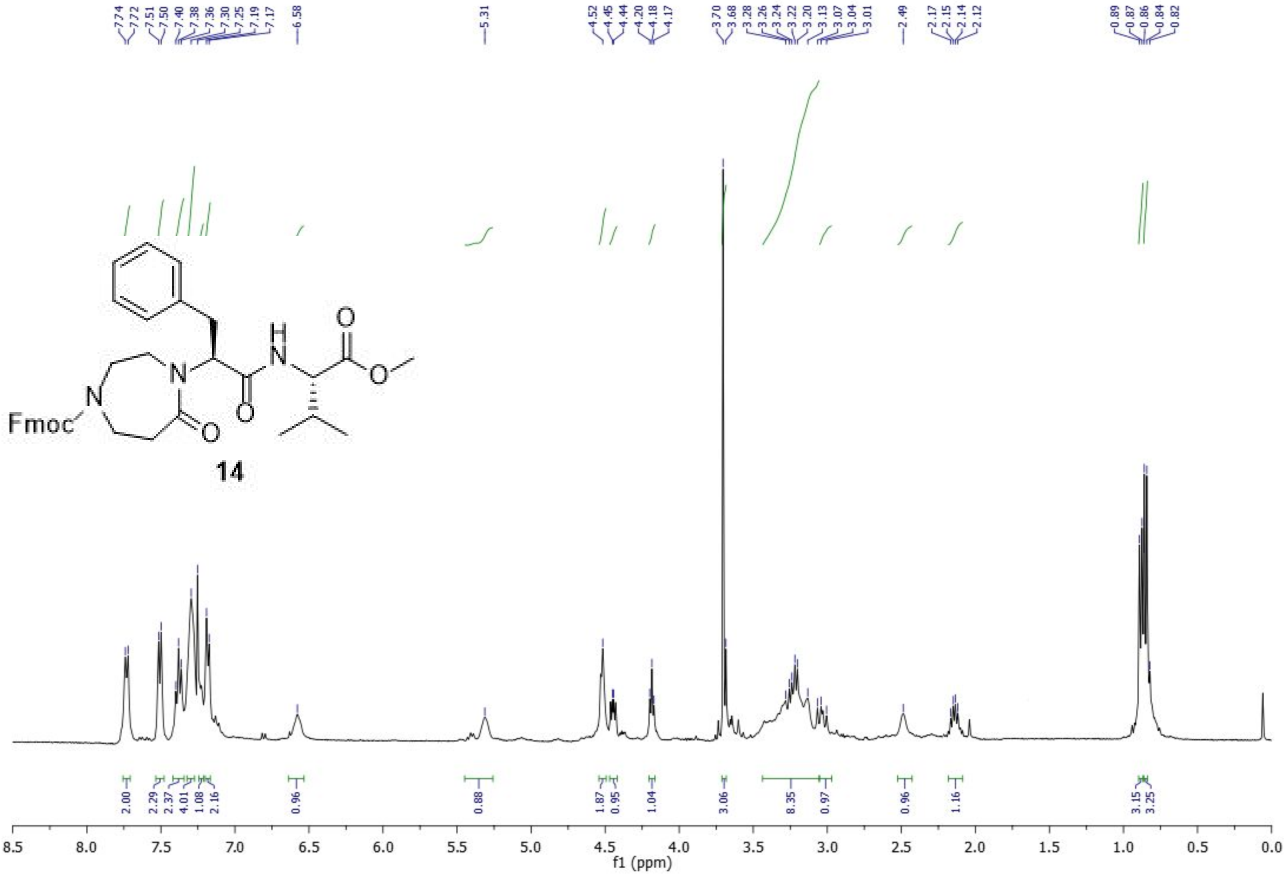

$^{13}C\{^{1}H\}$ NMR (100 MHz, $CDCl_3$)

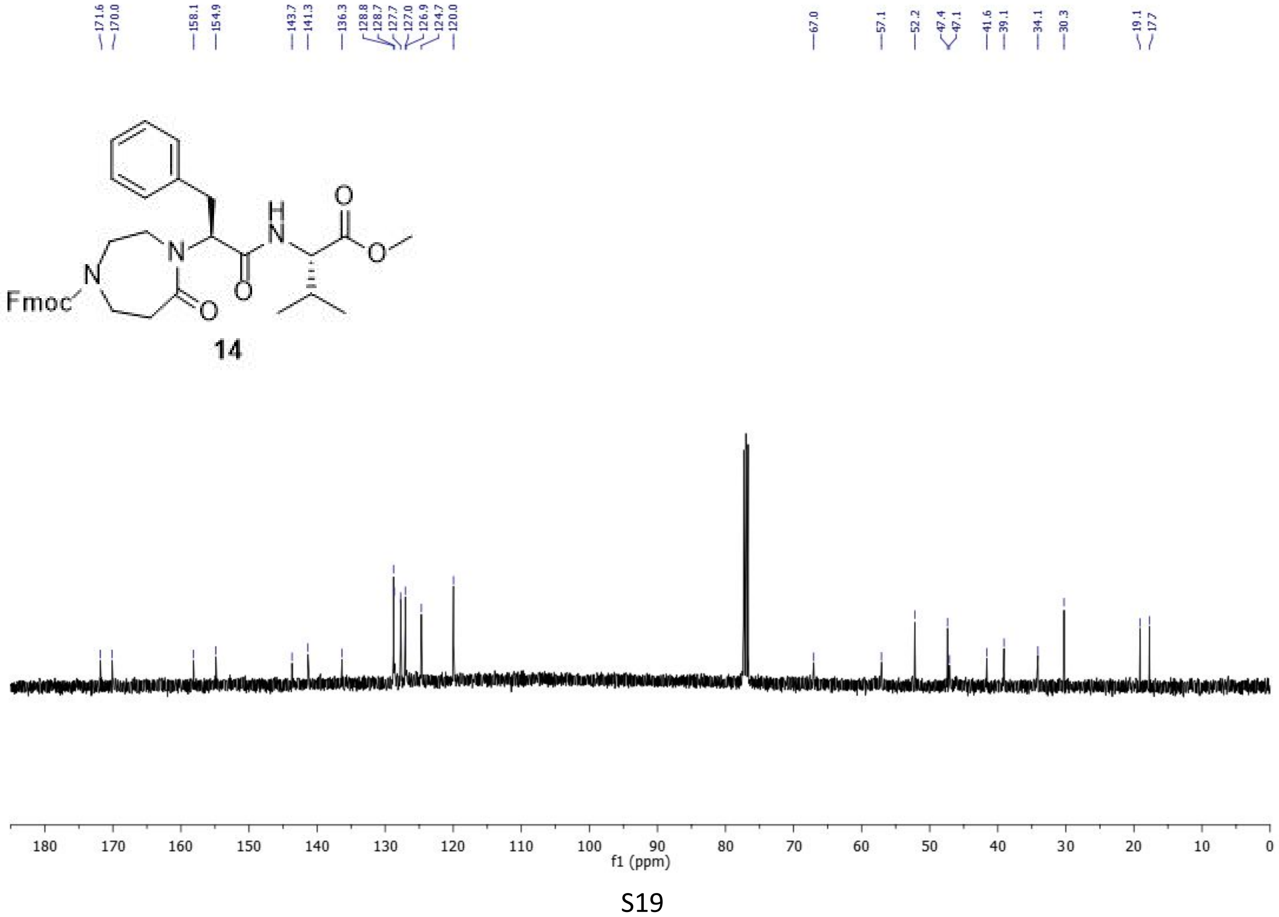

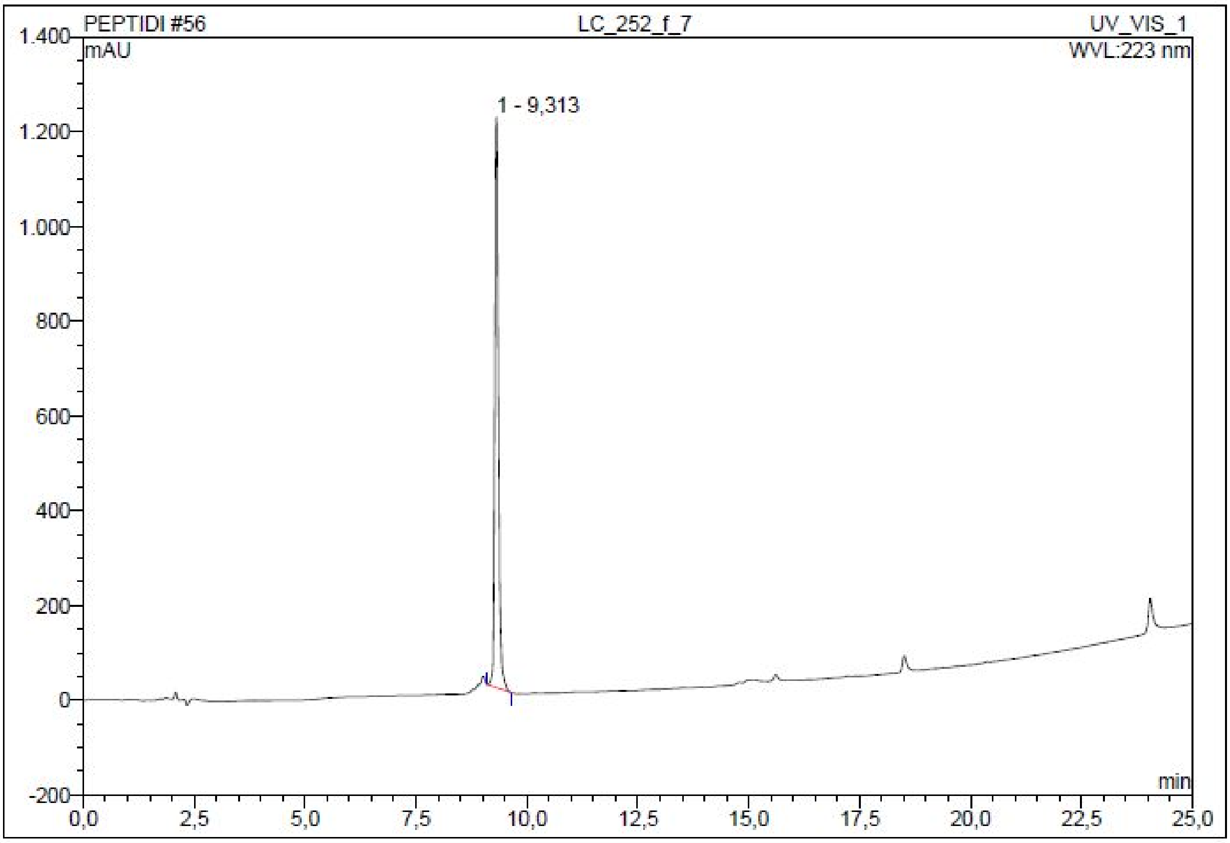

No.	Ret.Time min	Peak Name	Height mAU	Area mAU*min	Rel.Area %	Amount	Type
1	9,31	n.a.	1203,637	116,137	100,00	n.a.	BMB
Total:			1203,637	116,137	100,00	0,000	

Figure S1: HPLC chromatogram of peptide **15**.

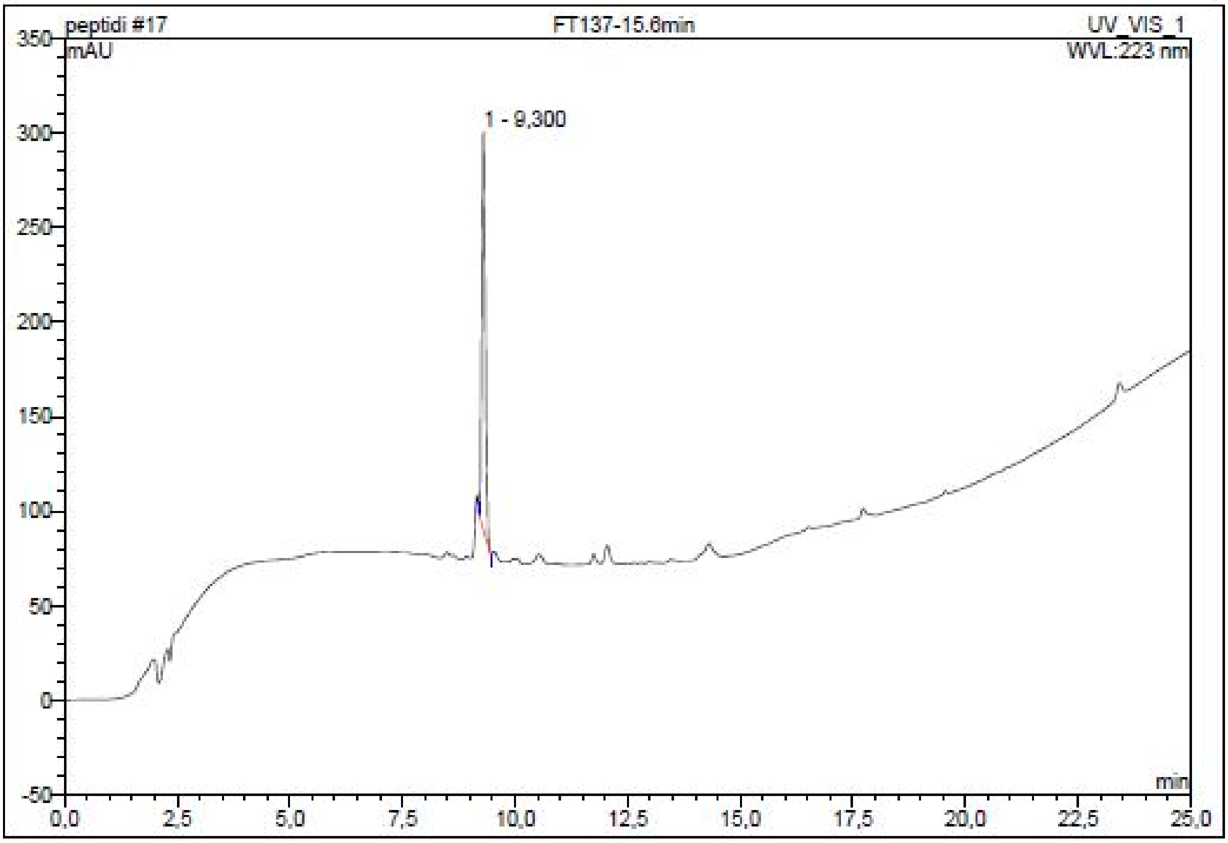

No.	Ret.Time min	Peak Name	Height mAU	Area mAU*min	Rel.Area %	Amount	Type
1	9,30	n.a.	210,440	18,988	100,00	n.a.	BMB
Total:			210,440	18,988	100,00	0,000	

Figure S2: HPLC chromatogram of peptide **16**

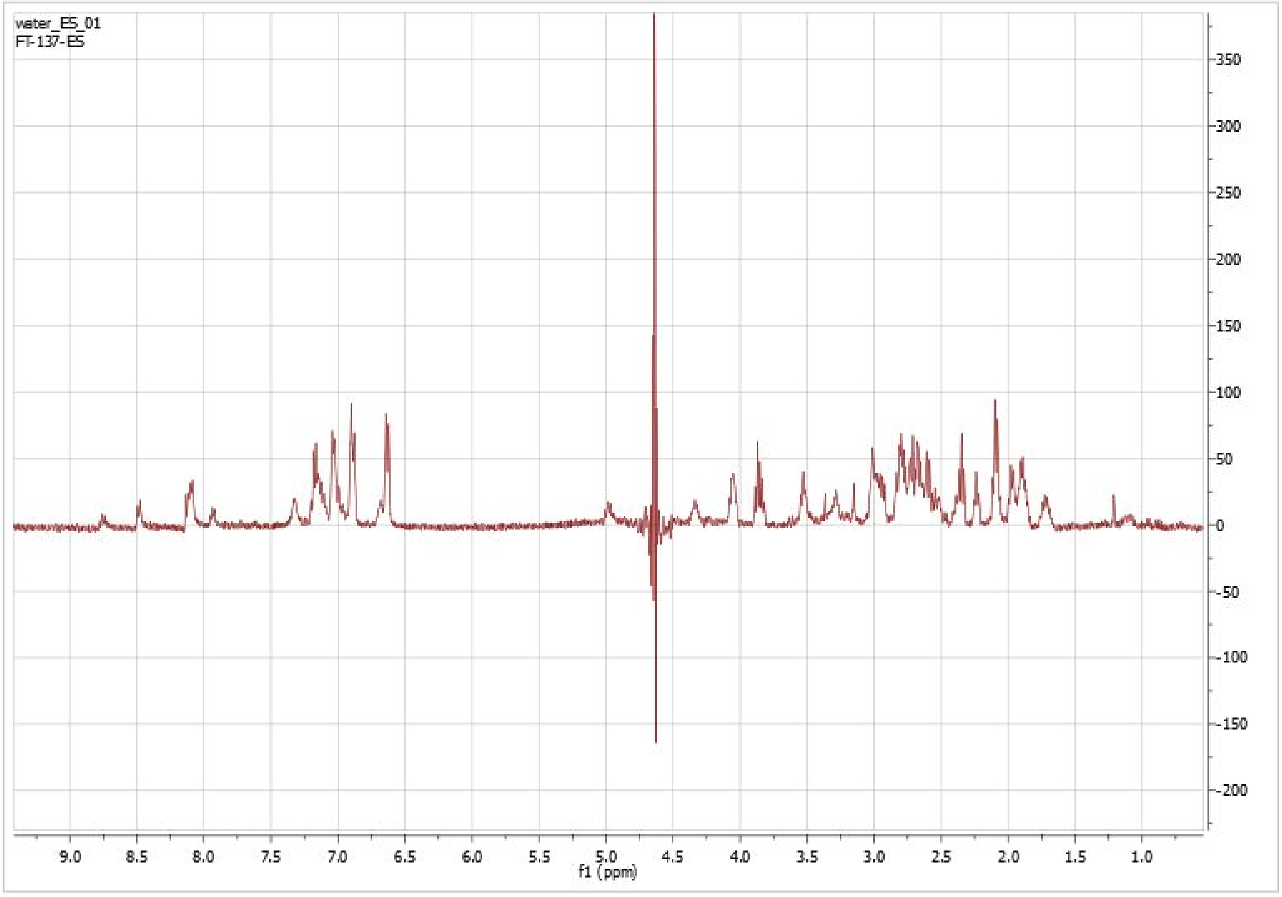

Figure S3: 1H-ES of peptide **16**

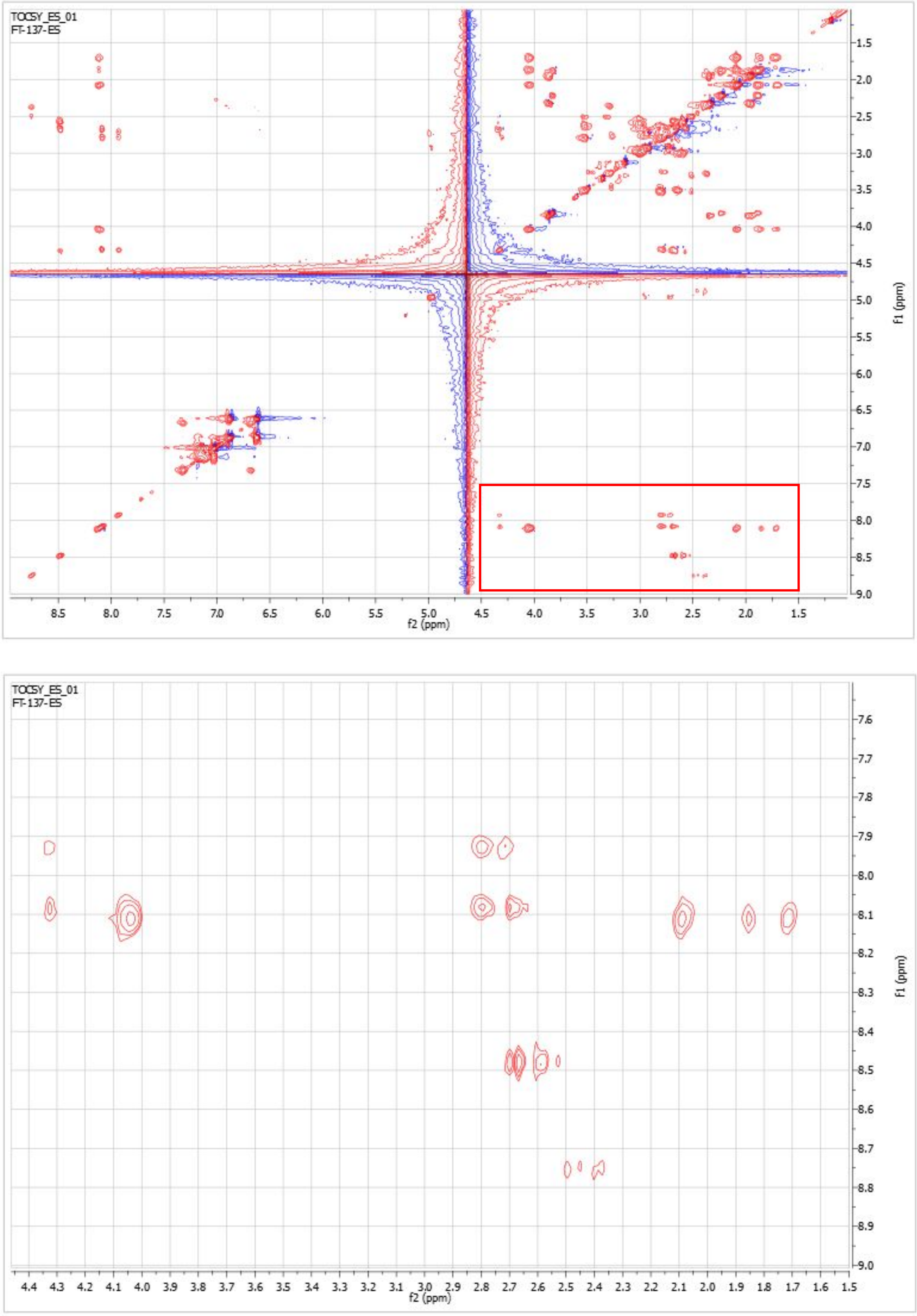

Figure S4: TOCSY-ES of peptide **16**

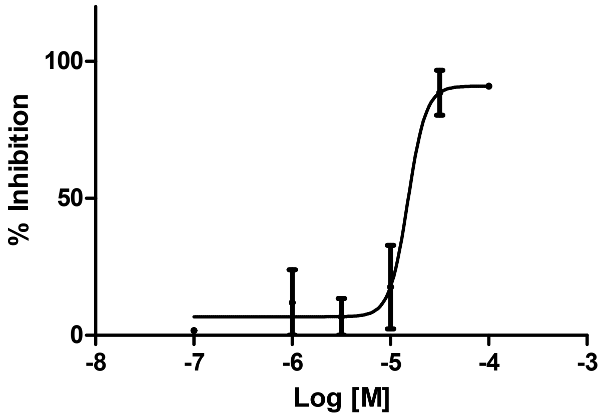

Figure S5: Inhibition curve of peptide **15** on 3CLPro enzyme. IC_{50} = 15 ± 6 µM. Experiments were conducted in triplicate.

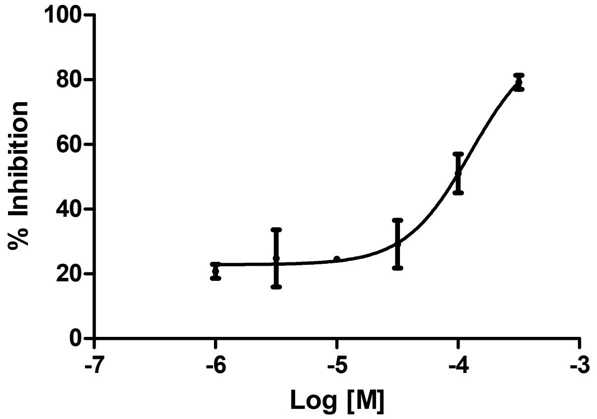

Figure S6: Inhibition curve of peptide **16** on 3CLPro enzyme. IC_{50} = 127 ± 76 µM. Experiments were conducted in triplicate.

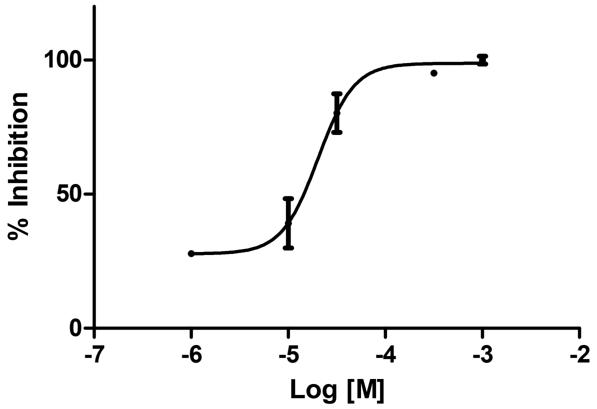

Figure S7: Inhibition curve of peptide **16** of Spike/ACE2 interaction. IC_{50} = 20 ± 5 µM. Experiments were conducted in triplicate.
